# Partners or passengers? Revisiting the association between diatoms and aquatic animals

**DOI:** 10.1002/brv.70191

**Published:** 2026-06-05

**Authors:** Gianluca Vacca, Elisa Falasco, Francesca Bona

**Affiliations:** ^1^ Department of Life Sciences and Systems Biology (DBIOS) University of Turin Via Accademia Albertina, 13 Turin 10123 Italy; ^2^ Alpstream Research Centre Frazione Sant'Antonio, 17 Ostana 12030 Cuneo Italy

**Keywords:** Bacillariophyceae, epizoic, animals, epibionts, commensalism, biological interactions

## Abstract

Numerous studies have revealed the importance of research on the communities capable of colonizing animal surfaces (epibionts) and the animals on which they live (basibionts). Very few studies have considered epizoic diatoms, and there are gaps and biases in our knowledge, including the choice of basibionts, the methods used, and the habitats in which they live. This literature review aims to (*i*) identify the main basibionts that host diatom communities and the diatom taxa associated with them, in both marine and freshwater environments; (*ii*) explore the key ecological factors shaping these diatom assemblages; and (*iii*) reassess critically the commonly applied concept of commensalism in epibiont–host relationships. Our literature review revealed that most research has been focused on charismatic basibiont groups, especially Chordata (notably Testudines and Cetacea), followed by Arthropoda and Mollusca, while taxa such as Bryozoa, Porifera, Cnidaria, Echinodermata, and Annelida have received far less attention. Most studies were carried out in marine settings, particularly in the Americas, and the number of publications has increased since the early 2000s. Taxonomic data on diatom communities shows that the genera *Navicula*, *Nitzschia*, *Cocconeis* and *Amphora* are the most frequently found as epizoic diatoms. In some cases, highly host‐specific diatoms have been documented, for example *Pseudohimantidium pacificum*, *Protoraphis atlantica*, *Falcula hyalina*, and *Sceptronema orientale* on copepods. From a functional point of view, ‘motile’ diatoms were dominant on Chordata, Mollusca, and Arthropoda, followed by the ‘high‐profile’ ecological guild. This dominance suggests active interaction between the organisms, supporting the development of well‐structured communities on the hosts. There are several types of interaction, depending on the host and epibiont considered. We propose the concepts of *triggers* and *drivers* as a framework to describe the initial diversity and development of the epibiont community on the host, and we interpret the nature of these relationships as falling along a continuum from commensalism to parasitism and mutualism. Although diatoms are considered valuable tools for environmental monitoring, we suggest that host displacement and interactions with substrates may be considered as limiting factors for the use of epizoic diatoms in environmental monitoring. However, they may provide useful insights into host ecology, such as the range of host displacement and habits, feeding behaviour, and ecological traits, helping to clarify the trophic and behavioural interactions among basibionts and epibionts. This integrative perspective enhances our understanding of both diatom dispersal and their ecological tolerance and adaptability across diverse environmental contexts.

## I. INTRODUCTION

A growing number of studies have emphasized the importance of thoroughly investigating the communities capable of colonizing animal surfaces, an effort that has become increasingly feasible with the advent of innovative techniques such as metabarcoding and DNA analysis. Despite this progress, the colonization dynamics of epizoic diatoms on living hosts remain poorly understood. Nevertheless, the occurrence of diverse diatom species on a wide range of animal taxa offers valuable insights into host behaviour, daily activity patterns, ecological interactions, and spatial distribution (Fayolle *et al*., 2016; Majewska *et al*., 2017*a*; Donato‐Rondón *et al*., 2018; Rivera *et al*., 2018; Vassal *et al*., 2021; Falasco *et al*., 2025). These insights have potential applications in conservation biology (Majewska *et al*., 2017*a*,*b*) and could inform targeted interventions for host species management in the case of allochthonous and/or invasive species (Falasco *et al*., 2018, 2025). At the same time, studying epizoic diatoms contributes significantly to our understanding of diatom ecology, particularly in terms of dispersal mechanisms, climatic tolerance ranges, colonization strategies, and substrate specificity. Given these wide‐ranging implications, a comprehensive review of current knowledge on epizoic diatoms, their associated hosts (basibionts), and the environments in which they occur will be useful to guide future research efforts. Therefore, the objectives of this review are: (*i*) to identify the main basibionts that serve as substrates for diatom colonization and the diatom taxa associated with them in both marine and freshwater environments; (*ii*) to determine the key factors influencing the structure of diatom communities on animal hosts; and (*iii*) to reassess critically the commonly used concept of commensalism in the context of basibiont–epibiont interactions.

Several national laws regulate the conservation of animals, aiming to protect wildlife and their populations. In this context, analysing epizoic diatoms could be useful for understanding the conservation needs of their host organisms, highlighting characteristics that affect their colonization, as well as the dynamics of interaction between animals and their epibionts. This type of interaction usually is considered as commensalism. However, according to its accepted definition (Veiga, 2016, p. 322), commensalism is an ‘ecological relationship between two organisms in which one species benefits from the association without impacting the fitness of the other’ – a definition which sometimes does not fit with epibiont–basibiont interactions, especially in the less‐studied freshwater environment where the advantages and disadvantages for the organisms involved remain to be clarified.

## II. METHODS

To obtain a list of suitable studies, we used the scientific databases *Scopus* and *Web of Science* (Burnham, 2006). Our search string used the key words ‘diatoms’, ‘diatom*’, ‘microalgae’ and ‘biofilm’, coupled with ‘epizoic’, ‘epibiosis’ and ‘epibionts’, as well as ‘diatom*’ AND ‘animal’ AND ‘host’ AND ‘interaction’. Furthermore, to ensure a comprehensive representation of studies, we also considered the references cited in the selected studies [according to the ‘backward snowballing’ approach (Wohlin, 2014); the research was conducted between April and May 2025]. A total of 257 studies were found, 19 of which were not considered because they were unavailable in the national university libraries consulted. A total of 105 studies were rejected because they were out of scope for this review (they considered green or red algae, animals, parasites, or bacteria as epibionts, or plants as basibionts). Thus, a total of 133 studies were included in the analysis (see online Supporting Information, Data S1 for full list of included studies). From each publication, we extracted information on the year of publication, the habitat investigated, the studied basibiont (including, when available, the specific body part examined), the methodologies employed, and the identified epizoic diatom species (Data S1).

## III. CURRENT KNOWLEDGE ON EPIZOIC DIATOMS

### (1) Literature overview

The first study on epizoic diatoms was published by Giesbrecht (1892), who observed the presence of epizoic diatoms on marine copepods in the Adriatic Sea. Subsequent early studies (see Fig. S1) include Bennett (1920), who analysed the formation of diatom biofilms on the skin of whales. Scientific publications on epizoic diatoms started to increase from the 1980s, when most studies were carried out in marine environments. From 1993 onwards, an increasing proportion of studies were conducted in freshwater and estuarine environments (e.g. Cupul‐Magaña & Cortés‐Lara, 2005; Radea *et al*., 2010) or on captive animals or dead organisms conserved in museums (e.g. Kanjer *et al*., 2020; Majewska & Goosen, 2020; Majewska *et al*., 2021). Most studies on epizoic diatoms have been conducted in North and South America (40.6% of the total studies; see Table S1 and Fig. S2), followed by Europe, Asia, Africa, Antarctica and Oceania. The highest number of studies were conducted in the USA (24 studies), representing 18% of the total studies on this topic, followed by South Africa (see Fig. S2). Freshwater ecosystems have been studied mainly in America and Europe, while marine environments were mostly the focus in Asia, Africa, Oceania and Antarctica. Currently, there are no records of studies on epizoic diatoms conducted in Russia, Canada, Greenland, Scandinavia, Poland, the Middle East, Central Africa and parts of South America (Fig. S2 and Table S1). Most studies (93%) used a morphological approach for diatom identification (of which 7% included DNA analyses; Table S1; Fig. S3); since around 2010 the use of DNA techniques to study epizoic diatoms has been increasingly common (e.g. Martínez‐García *et al*., 2011; Hooper *et al*., 2019; Ashworth *et al*., 2022*b*).

### (2) Epizoic diatoms across aquatic environments

Epizoic diatoms have mostly been studied in the marine environment (with 99 out of 133 studies; Table S1, Fig. 1), followed by the freshwater environment (22 studies; Fig. 1) and ‘marine and culture’ with laboratory experiments (7 studies; Fig. 1). Although museum collections may offer a valuable opportunity to study a wide range of biotic substrates, this approach was used in only one study in the reviewed literature (Kanjer *et al*., 2020).

**Fig. 1:**
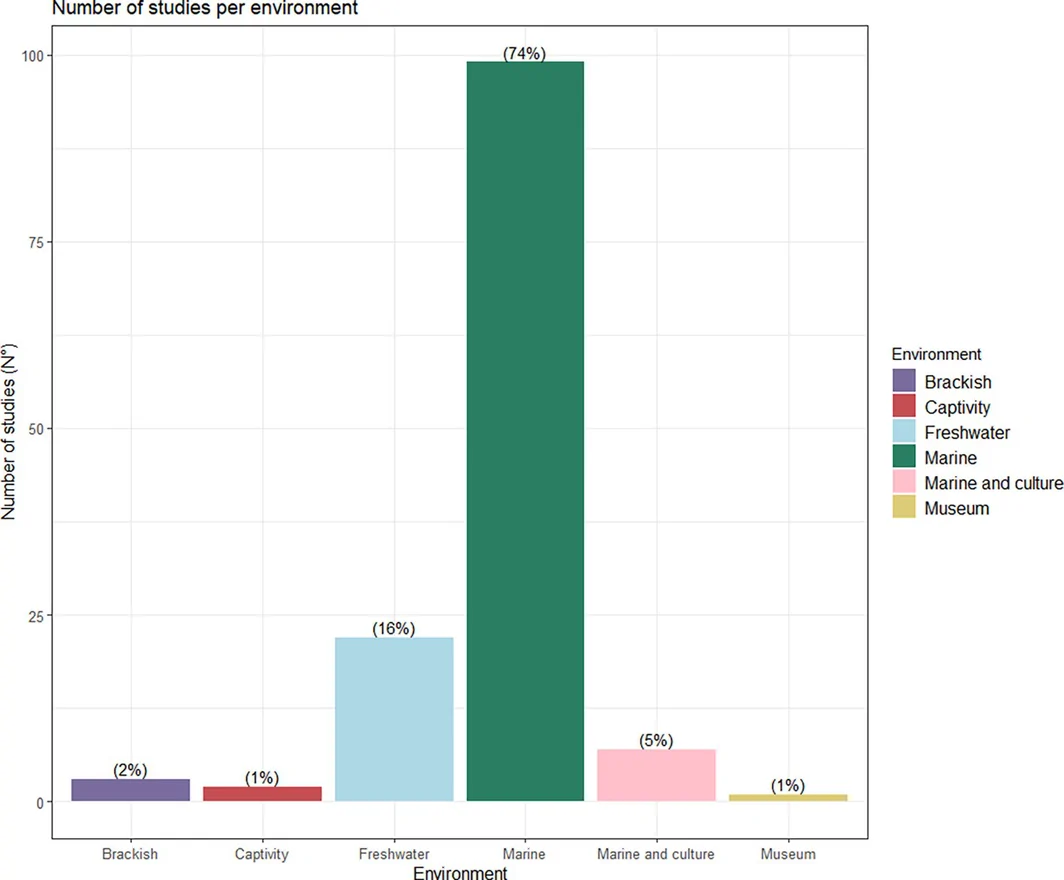
Graphical representation of the environments examined in published literature on epizoic diatoms, along with the corresponding number of studies (134 study areas in 133 papers. Figure created in ‘ggplot’ and ‘tidyverse’ (Wickham *et al*., 2019, 2023). Both studies classified as ‘captivity’ were conducted on marine organisms, specifically on sea turtle‐associated diatom communities.

A complete list of the epizoic diatoms described in the literature and their basibionts (at the phylum level), including those species first formally described as epizoic because they were found on animals, is provided in Table S2 and Fig. 2 (see also Section III.3). Epizoic diatoms belonging to the genera *Navicula*, *Amphora*, *Nitzschia* and *Cocconeis* appear to be common in marine environments. In freshwater ecosystems, the most frequently recorded species in literature were *Achnanthidium minutissimum*, *Cocconeis placentula*, *Navicula* spp., *Ulnaria ulna* and *Gomphonema parvulum* (Table S2). At least five genera have been described as new to the science and as strictly epizoic (i.e. *Bennettella*, *Epipellis*, *Epiphalaina*, *Falcula* and *Protoraphis*), along with additional genera represented by species recorded and described for the first time as epizoic (*Chelonicola*, *Medlinella*, *Tursiocola*, *Plumosigma*, *Sceptronema*, *Pseudohimantidium* and *Chelonicola*; see Table S2). Moreover, a total of 49 epizoic diatom species (and varieties) have been first formally described from animal substrates. In addition, two taxonomic reassignments have been made: *Cocconeis ceticola* was transferred to *Bennettella ceticola* by Holmes (1985) and *Stauroneis omurai* to *Tursiocola omurai* by Denys (1997), the latter representing an obligate epizoic species. Notably, more than half of the new identifications and species descriptions have been published in the past 25 years, reflecting the growing interest in this field of research (see Fig. S1).

**Fig. 2:**
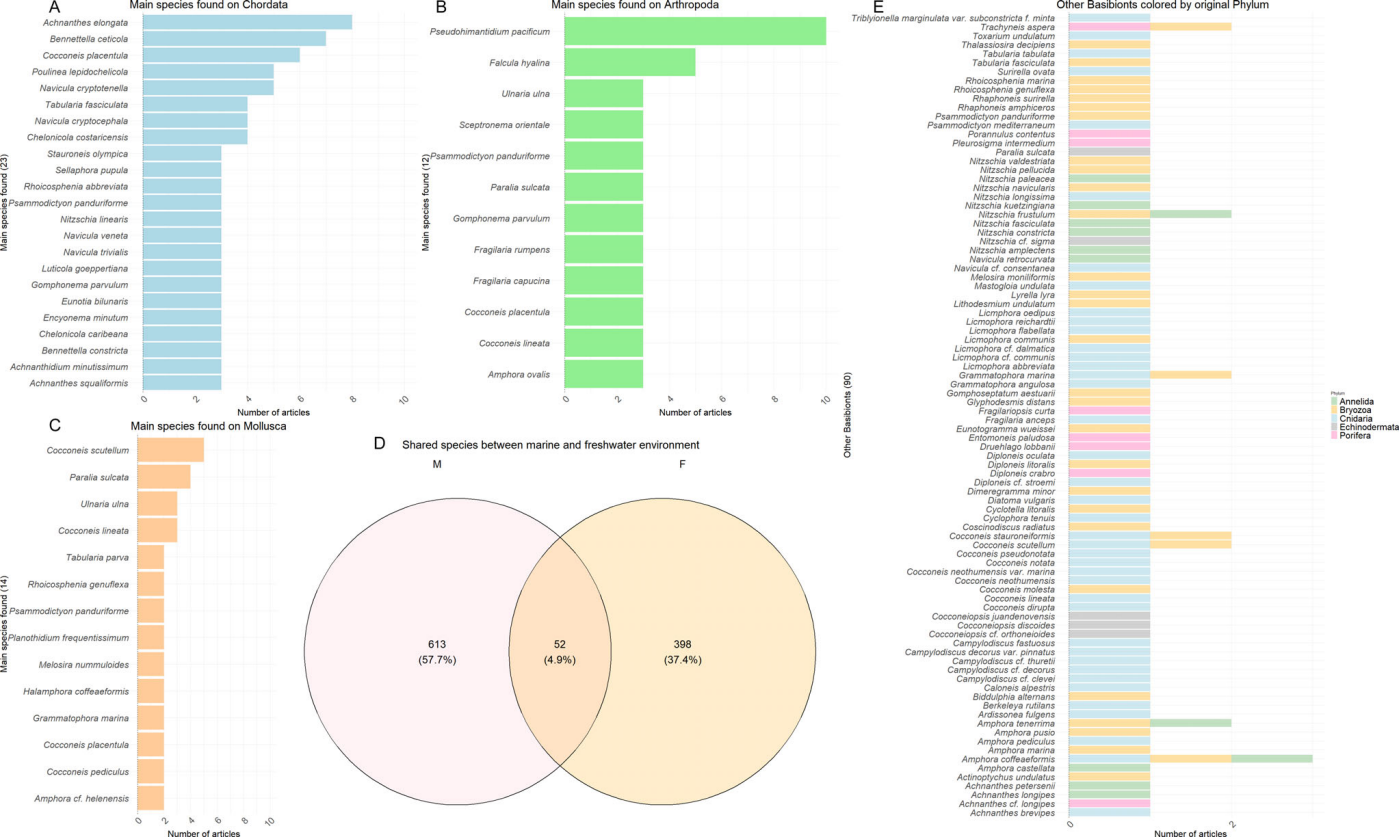
Main diatom taxa found on Chordata (A), Arthropoda (B), and Mollusca (C). Additionally, the number of diatom species shared between marine (= M) and freshwater (= F) environments (D) and the main diatom taxa found on other basibionts, coloured according to the basibiont phylum on which the species was originally recorded (E). The *x*‐axis indicates the number of articles reporting each species. Note that ‘main taxa’ refers to taxa reported in at least three articles for Chordata and Arthropoda, at least two articles for Mollusca, and all taxa recorded for ‘other basibionts’. Species reported as ‘spp.’ were removed from the figures to improve graphical clarity, but are retained in the final species list and used in all analyses (see Table S2 for the complete species list). Diatom species and synonyms were updated according to the most recent taxonomy using OMNIDIA software (6.1.7; Lecointe *et al*., 1993). Figure created using ‘gridExtra’, ‘Venn’ and ‘NetworkD3’ (Allaire *et al*., 2017; Auguie & Antonov, 2017; Dusa & Dusa, 2018).

Most descriptions of obligate epizoic diatoms were conducted in marine environments (45 species), with only two new epizoic species and two varieties reported from freshwater habitats to date (*Mastogloia sterijovskii* and *Synedra cyclopum –* var. *incisa* and var. *2*; see Table S2). In addition, in freshwater environments, most taxa found on animals also occur in the surrounding environment and are capable of colonizing abiotic substrates (see Table S2). Conversely, marine environments have a higher number of obligate epizoic diatoms (e.g. species of *Bennettella*, *Craspedostauros*, *Chelonicola*, *Poulinea*, *Medlinella*, and *Plumosigma*, and the species *Epiphalaina aleutica*, *Falcula hyalina*, *Sceptronema orientale*, *Pseudohimantidium pacificum*, *Achnanthes elongata* and *A. squaliformis*; see Table S2; Fig. 2). However, Van de Vijver *et al*. (2020) suggested that epizoic taxa may be more common in the marine habitat due to a lack of suitable abiotic substrates for the development of benthic communities in this environment, which therefore are replaced by biotic surfaces provided by living animals. This idea is also supported by Majewska *et al*. (2023). Moreover, much of the shallow marine benthic environment still remains unexplored with respect to epizoic diatoms and compiling an exhaustive marine diatom flora will be extremely challenging. Consequently, we cannot rule out the possibility that many taxa currently regarded as obligate epizoic may, in fact, also be capable of colonizing abiotic substrates. Future in‐depth studies on marine diatoms may help to clarify whether the epizoic lifestyle of these organisms is truly obligate.

### (3) Epizoic diatom–host associations

Chordata was the most studied phylum across all environments (66 studies; 50%; Fig. 3A), with equal representation in marine and freshwater environments (50%; Table S2).

**Fig. 3:**
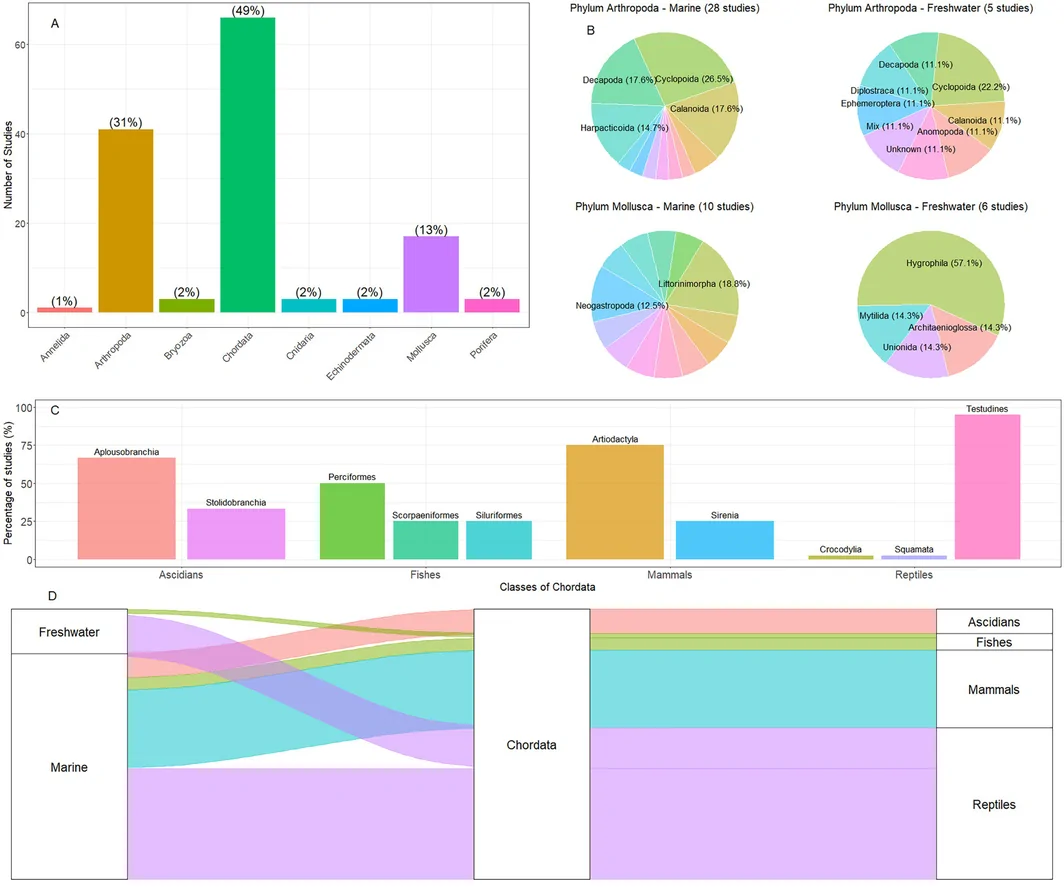
The basibionts studied in the epizoic diatom literature (138 taxa from 133 studies). For the order Artiodactyla (Prothero *et al*., 2022), records include only the infraorder Cetacea. (A) Number of studies reporting basibionts for each phylum. (B) Percentages of studies for different basibiont taxa within the phylum Arthropoda (top row, marine *N* = 28 studies; freshwater *N* = 5) and Mollusca (bottom row, marine *N* = 10; freshwater *N* = 6); only the percentage of the orders studied a large number of times (i.e. representing more than 10% of the total studies) are shown in the figure for improved graphical clarity (note: ‘mix’ refers to a group of organism orders that could not be identified from the individual study). (C) Percentage of basibiont taxa recorded for each class of Chordata (*N* = 66 studies). (D). Graph showing the number of studies conducted for each habitat (freshwater and marine) and each class of Chordata.

Among chordates, epizoic diatoms were reported most often for the order Testudines (Fig. 3C), for example from the green turtle (*Chelonia mydas*). Regarding the order Artiodactyla, the most frequently reported basibionts were from the Balaenopteridae, Delphinidae, and Phocoenidae. Several studies reported fish species as basibionts from the families Perciformes, Scorpaeniformes (e.g. *Scorpaena* spp.) and Siluriformes. No studies have been conducted on amphibians, perhaps due to the delicate nature of amphibian skin, which makes removing biofilms challenging. The phylum Arthropoda was investigated in 41 studies, corresponding to 31% of the analysed literature. The most common orders of arthropods included in the marine studies were the copepod orders Cyclopoida, Calanoida and Harpacticoida and the decapods (Decapoda), which together were named in more than 70% of all the studies carried out on this phylum (Fig. 3B). In freshwater ecosystems, the main arthropod order for which basibionts were described was Cyclopoida. Some epizoic diatom species have been recorded on all three major basibiont taxa studied (Chordata, Arthropoda, and Mollusca; Fig. 2A–C), mainly belonging to the generalist genus *Cocconeis*. However, notable differences emerge in the species most frequently observed on each host group. *Achnanthes elongata*, *Bennettella ceticola*, and *Poulinea lepidochelicola* dominate on Chordata (Fig. 2A), while *Pseudohimantidium pacificum* and *Falcula hyalina* are the most common species found on Arthropoda (Fig. 2B). Lastly, *Cocconeis scutellum* and *Paralia sulcata* were found especially on Mollusca (Fig. 2C). These differences are certainly related, although not exclusively, to the environment considered (Fig. 2D).

## IV. FUNCTIONAL DYNAMICS OF EPIZOIC DIATOM–HOST INTERACTIONS

### (1) Functional ecology of epizoic diatoms

Diatom epibionts living in freshwater environments were allocated to ecological guilds according to Passy (2007) and Rimet & Bouchez (2012). There was a clear dominance of ‘motile’ (defined as ‘free of both resource limitation and disturbance stress, because it has the physical capability of selecting the most suitable habitat’; Passy, 2007, p. 172). and ‘high‐profile’ diatoms (defined as ‘comparatively resource‐unlimited but disturbance‐stressed, i.e. it has easy access to nutrients and light but is subject to high current or grazer disturbance’; Passy, 2007, p. 172) on all the three main basibiont taxa in freshwater environments (Fig. 4). On Chordata and Mollusca, the dominant epibiont guild was ‘motile’ (about 45% of the total organisms), while on Arthropoda, the proportion classified as ‘motile’ was slightly lower (34%). It is plausible that the ability of diatoms to utilize nutrients and substances released by their host organisms will favour the formation of a highly structured periphytic community (Passy, 2007). For example, the ‘high‐profile’ guild is favoured by its strong competitiveness under conditions of high nutrient concentrations, and potentially outcompetes the ‘low‐profile’ group (defined as ‘likely to be resource‐stressed but disturbance‐free, i.e. it experiences resource limitations such as shading and nutrient depletion’; Passy, 2007, p. 172) through increased shading. Based on our findings, light availability and nutrient concentrations seem to be the main factors that shape functional communities on the hosts considered, more so than the flow speed.

**Fig. 4:**
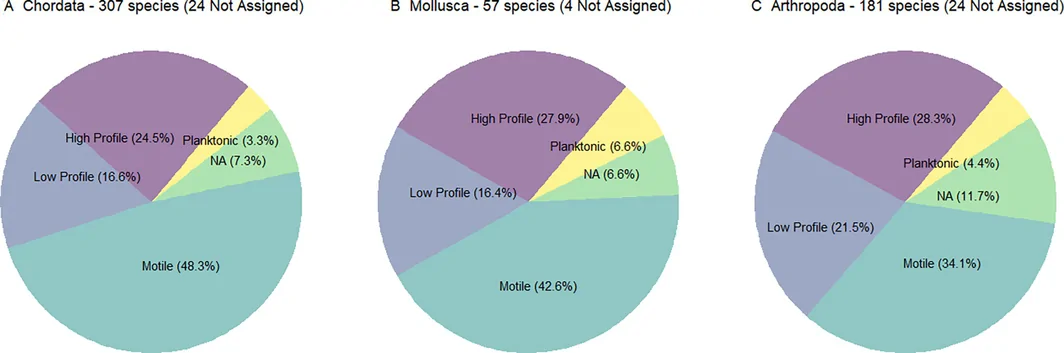
Ecological guilds of epibionts found on the three main basibiont phyla: (A) Chordata; (B) Mollusca; (C) Arthropoda. The number of epibiont diatom species used in each assessment is given above the charts. Diatom species were classified into ecological guilds according to Passy (2007) and Rimet & Bouchez (2012). Note: NA indicates diatoms not present in the OMNIDIA database updated to 2025. For more information on the meaning of individual ecological guilds, refer to Passy (2007) and Rimet & Bouchez (2012).

In general, the dominance of ‘motile’ diatoms (particularly on Chordata) could possibly be explained by the ability of this guild to move on the host to reach parts of the host body less exposed to hydrodynamic forces (Passy, 2007). By contrast, a greater number of planktonic diatoms might be expected on the shells of molluscs as their sedentary habits are more likely to allow diatom sedimentation. We cannot yet exclude the possibility that some ‘low‐profile’ diatom taxa such as *Cocconeis* spp., which are commonly epiphytes, may be correlated with the presence of macroalgae/filamentous algae on the basibiont surface. Finally, the functional composition of diatom communities on Arthropoda might be related to the size of these basibionts, with ‘high‐profile’ diatoms possibly undergoing positive selection due to the smaller area for attachment available on these hosts (especially copepods).

### (2) ‘Triggers’ and ‘drivers’

Diatom colonization dynamics on biotic substrates could depend on aspects of basibiont behaviour/characteristics or on environmental variables/surface features that we define as ‘triggers’ and ‘drivers’, respectively (Fig. 5). The term ‘trigger’ refers to behavioural and ecological factors of the basibiont, such as daily activity patterns, feeding and mating behaviour, contact with the surrounding environment, movement, and interspecific interactions, that facilitate initial contact between diatoms and the basibiont surface, i.e. the conditions that enable diatoms to reach and potentially colonize the host's body (Fayolle *et al*., 2016; Majewska *et al*., 2017*b*; Donato‐Rondón *et al*., 2018; Rivera *et al*., 2018; Vassal *et al*., 2021; Falasco *et al*., 2025). For example, direct contact between host organisms may facilitate host colonization by epibionts (Holmes, Nagasawa & Takano, 1993; Denys & de Smet, 2010; Frankovich, Sullivan & Stacy, 2015*a*; Frankovich *et al*., 2016; Majewska *et al*., 2017*b*, 2021; Majewska, 2021). This contact may allow diatoms to spread from one individual to another, contributing to the formation of epizoic communities across host populations. A better understanding of the mechanisms involved in epizoic diatom colonization dynamics thus could indirectly provide information on host behaviour and spatial distribution (Denys & de Smet, 2010; Frankovich *et al*., 2015*a*; Hooper *et al*., 2019; Majewska, 2021; Majewska *et al*., 2021; Roubeix *et al*., 2021; Falasco *et al*., 2025). Interestingly, Chordata and Arthropoda show a much higher number of shared species in freshwater environments (22.2%) than do these taxa in marine sites (3.3%; Fig. S4), perhaps reflecting a greater similarity of occupied niches by basibionts in freshwater environments. For example, chordates and arthropods studied in freshwater environments, particularly species of the orders Testudines, Siluriformes (both Chordata) and Decapoda (Arthropoda), show comparable benthic habits, which may result in a more similar epibiont diatom community (Rivera *et al*., 2018; Falasco *et al*., 2018). Conversely, in the marine environment, chordates are more commonly large nektonic organisms (including large mammals, such as whales; Santos *et al*., 2019), whereas representatives of Arthropoda are mostly planktonic (especially copepods), making shared epibiota less likely.

**Fig. 5:**
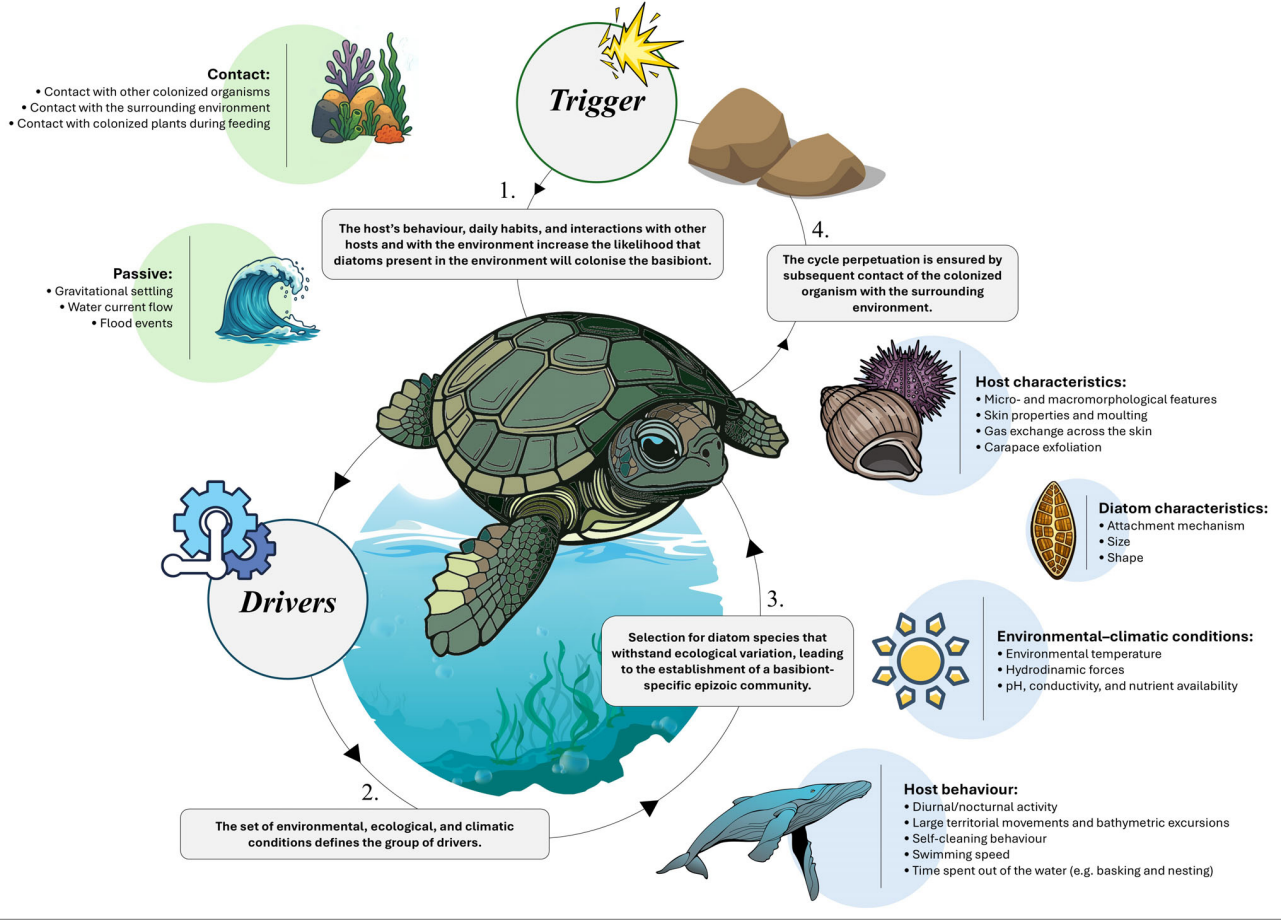
Illustration of our concepts of ‘triggers’ and ‘drivers’ responsible for the formation of epizoic diatom communities on basibionts.

We use the term ‘drivers’ to refer to all the environmental conditions, the characteristics of the basibiont surface, as well as the morphological or physiological features of diatoms that act as ecological filters on diatom communities, determining epibiont species composition (Fig. 5) (Falasco *et al*., 2025; Rivera *et al*., 2018). These factors select for diatoms that are able to survive on the outer surface of the basibiont which represents a specific microhabitat (Croll & Holmes, 1982; Donato‐Rondón *et al*., 2018; Hooper *et al*., 2019; Azari *et al*., 2020; Majewska *et al*., 2021). For example, the hosts' biological surface will have specific and complex characteristics such as roughness (Majewska *et al*., 2017*b*; Donato‐Rondón *et al*., 2018; Roubeix *et al*., 2021) and stickiness (Azari *et al*., 2020), which are likely important aspects in shaping diatom communities. Other selective agents include exposure to hydrodynamic forces, sunlight, and temperature changes (Riaux‐Gobin *et al*., 2021; Roubeix *et al*., 2021; Vassal *et al*., 2021), the composition of the surface (e.g. skin, chitin), as well as the presence of chemicals or gas exchange of the host (Vassal *et al*., 2021; Falasco *et al*., 2025). All these conditions can result in the selection of specific physiological and morphological features of the epibionts (Wu & Bergey, 2017). Epizoic species have a variety of mechanisms to allow attachment to the host's surface (Majewska *et al*., 2017*a*,*b*; Donato‐Rondón *et al*., 2018; Azari *et al*., 2020; Shen *et al*., 2024), for example some diatoms have stalks with a large basal area for attachment to smooth surfaces (Donato‐Rondón *et al*., 2018).

Diatom size can also be considered a driver (Kaleli *et al*., 2018; Majewska *et al*., 2021). Some authors have argued that smaller‐sized diatom species seem to be favoured in epizoic communities (Kaleli *et al*., 2018; Riaux‐Gobin *et al*., 2020; Majewska *et al*., 2021), although others have rejected this hypothesis (Pavlov *et al*., 2016; Huang *et al*., 2025). For the species in our data set for which size information was available (79% of epibionts on Chordata; 70% on Arthropoda; 62% on Mollusca), we categorized them into size classes according to Rimet & Bouchez (2012). The results (Fig. S5) suggest that larger sizes may be more prevalent for all three basibiont groups. Over half of the epizoic diatom species belonged to size classes 4 and 5 for all three basibiont phyla. It is hypothesized that larger diatom cell size could provide a greater contact surface area, thereby enhancing the ability to attach to the host, despite the increased hydrodynamic forces that may result. In addition, a slender shape would enhance the ability of diatoms to resist hydrodynamic forces during host movements (Clarson *et al*., 2009). However, diatom shape could not be assessed for all the species considered in our analysis. Diatom survival will also be related to position on the host and attachment mechanism, but these require further investigations.

### (3) Diatoms on Chordata

The diatoms found most frequently on chordate basibionts were *Achnanthes elongata*, *Bennettella ceticola*, *Cocconeis placentula*, *Poulinea lepidochelicola*, *Navicula cryptotenella*, *Tabularia fasciculata*, *Navicula cryptocephala* and *Chelonicola costaricensis* (Fig. 2A; Table S2). Chordata hosts the highest number of diatom genera (126 genera; see Fig. S6), almost half of which were found exclusively on this group. For instance: *Tursiocola* was found almost exclusively on turtles, manatees and whales. *Epiphalaina* and *Bennettella* were found only on whales. The species *Poulinea lepidochelicola* was only found on the order Testudines (*Lepidochelys olivacea*), while the genera *Chelonicola* and *Olifantiella* were only found on the order Testudines (*Caretta caretta*, *Lepidochelys olivacea* and *Chelonia mydas*). Table S2 provides information on all species considered as strictly associated with a specific basibiont.

Many factors influence the presence of diatoms on Chordata. For example, grazing on phanerogams by marine turtles causes sediment suspension, from which diatoms can initially settle on the carapace surface (Rivera *et al*., 2018), and a benthic habit, such as that of the freshwater wels catfish (*Silurus glanis*), is also likely to favour colonization by benthic diatoms (Falasco *et al*., 2025). The diatom genus *Cocconeis* is typically epiphytic on aquatic plants (De Stefano, Marino & Mazella, 2000), suggesting that contact with macrophytes may allow initial colonization by epizoic communities.

Among the ‘drivers’ shaping epizoic diatom communities on turtles, basking behaviour outside water and nesting behaviour may be key determinants of their epibiont diatom communities, both in marine (Donato‐Rondón *et al*., 2018; Wu & Bergey, 2017) and freshwater environments (Roubeix *et al*., 2021; Vassal *et al*., 2021). This may explain the presence of genera such as *Luticola*, which are highly resistant to dry conditions (Wu & Bergey, 2017). Indeed, *Luticola* spp. can close pores on their valve surface when exposed to high temperatures, reducing water loss (Wu & Bergey, 2017). *Mastogloia sterijovskii* exhibits strong desiccation tolerance, aided by a protective extracellular mucilaginous matrix; this species is known only from the turtle *Emys orbicularis* (Pavlov *et al*., 2016). The scouring by sand that accompanies nesting activity in turtles may also be a ‘driver’, favouring diatom taxa that adhere more strongly to the surface of the basibiont. In marine environments, the high tolerance to changes in salinity of *Tursiocola ziemanii*, *T. varicopulifera* and *T. costata* promotes their survival during the long movements of host organisms (Frankovich *et al*., 2015*a*). Morphological features of diatoms can also influence their pattern of colonization on Chordata (Agudelo & Grzonka, 2019). For instance, *Tursiocola* and *Epiphalaina* have heteropolar valves, which probably favour cell attachment to the skin of their hosts, after cell division (Denys, 1997). Structures such as a well‐developed pseudosepta may enhance resistance to hydrodynamic forces during the movements of their hosts (Denys, 1997). In addition, a larger attachment area may reduce the likelihood of displacement from hosts such as sperm whales (*Physeter macrocephalus*), which can reach speeds of up to 45 km/h (Aoki *et al*., 2007). Some chordate orders, such as amphibians, remain unstudied as basibionts due to ecological, methodological, and historical factors. Amphibian skin is dynamic, mucus‐coated, antimicrobial, and is shed regularly, presumably making long‐term diatom colonization difficult (Larsen, 2021). Many amphibian species now are threatened or protected, requiring non‐invasive sampling and strict ethical approval. Research has historically focused on easily collectable invertebrates and macrophytes, and on charismatic vertebrates (e.g. turtles, dolphins, whales) (Metrick & Weitzman, 1996). Research on diatoms associated with birds has primarily focused on long‐distance dispersal, either *via* twigs transported during nest construction or through the digestive system following feeding activities (Foged, 1951, 1953), and there is no information available on strictly epizoic relationships in this taxon.

### (4) Diatoms on Arthropoda

Diatom epibionts on arthropods are mostly generalist species. Those found on several arthropod taxa include *Pseudohimantidium pacificum*, *Falcula hyalina*, *Ulnaria ulna*, *Sceptronema orientale*, *Psammodictyon panduriforme*, *Paralia sulcata*, *Gomphonema parvulum*, *Fragilaria rumpens*, *Fragilaria capucina*, *Cocconeis placentula*, *Cocconeis lineata* and *Amphora ovalis* (Fig. 2B). On copepods and cladocerans, diatoms have been found on different parts of the exoskeleton (Russell & Norris, 1971; González & Vergara, 1984; Sahu *et al*., 2015; Padmakumar *et al*., 2015; Li *et al*., 2020; Purushothaman *et al*., 2021; Sandra, Purushothaman & Padmakumar, 2021; Bhaumik, Fernandes & Biswas, 2022; Bhowal *et al*., 2022). Movement of arthropod appendages may promote initial colonization by contact with the substrate and may act as a driver by improving oxygen exchange. As in the Chordata, diatoms can be concentrated in the regions of greatest contact during mating (Russell & Norris, 1971; González & Vergara, 1984).

Diatoms on copepods seem to have intimate ecological interactions with their host (González & Vergara, 1984' Chiavelli, Mills & Threlked, 1993; Gárate‐Lizárraga & Esqueda‐Escárcega, 2016). Several species such as *Pseudohimantidium pacificum*, *Protoraphis atlantica*, *Sceptronema orientale*, *Falcula hyalina* and *Synedra cyclopum* were found exclusively on arthropods, suggesting a close ecological relationship with their hosts. *Synedra cyclopum* is considered highly adapted to an epizoic lifestyle (Gaiser & Bachmann, 1994). This species was first described by Brutschy (1922) from the body surfaces of *Cyclops* sp. and may be the only obligate epizoic diatom known to occur on Arthropoda in freshwater habitats. Freshwater *Synedra cyclopum* seems to be closely linked with the arthropod genus *Daphnia*; it is absent in its absence and present at high abundances when *Daphnia* populations are large (Chiavelli *et al*., 1993). Similarly, *Falcula hyalina* is found in strict association with *Acartia steueri* (Prasad, Livingston & Ray, 1989; Donadel & Torgan, 2016). *Pseudohimantidium pacificum* has a specific interaction with *Corycaeaus* sp. (González & Vergara, 1984; Padmakumar *et al*., 2015), one of the genera most affected by epizoic diatom colonization (Sahu *et al*., 2015). Similarly, *Protoraphis atlantica* seems to colonize preferentially the genus *Candacia* (Li *et al*., 2020; Purushothaman *et al*., 2021).

Regarding the specific conditions that facilitate initial colonization of the basibiont, behavioural features of the arthropod hosts are likely to have key roles. A more sedentary habit of the hosts and lower movement speeds may be important factors in the colonization dynamics of copepods (Pan *et al*., 2021). The behaviour of larger arthropods such as some crustaceans (D'Alelio *et al*., 2010; Falasco *et al*., 2018), including their daily activity as well as interactions with the benthic substrate, will facilitate initial contact between the basibiont and colonizing diatoms. In addition, environmental conditions can also act as drivers for diatom community development, which is strongly driven by light exposure (Falasco *et al*., 2018) and climatic conditions (Chiavelli *et al*., 1993; Patil & Anil, 2000), with seasonal patterns observed (Gaiser & Bachmann, 1993).

### (5) Diatoms on Mollusca

The phylum Mollusca has been less studied with respect to epibiont diatoms (Fig. 3; about 13% of all studies), with the main basibionts belonging to the taxa Neogastropoda and Littorinomorpha in marine environments and Hygrophila in freshwater ecosystems. The diatoms found on the widest host range were *Cocconeis scutellum*, *Paralia sulcata*, *Ulnaria ulna*, *Cocconeis lineata*, *Tabularia parva*, *Rhoicosphenia genuflexa*, *Psammodictyon panduriforme*, *Planothidium frequentissimum*, *Melosira nummuloides*, *Halamphora coffeaeformis*, *Grammatophora marina*, *Cocconeis placentula*, *Cocconeis pediculus* and *Amphora cf. helenensis* (Fig. 2C; Table S2).

Some studies suggest that the morphology and age of the host shell play key roles in the development of the diatom community of molluscs (Keating & Prezant, 1998; D'Alelio *et al*., 2010; Takada & Kanavillil, 2024). Molluscan growth dynamics affect the size and structure of their shells, both at micro‐ and macroscales (D'Alelio *et al*., 2010) which, in turn, affects diatom community composition and structure (Keating & Prezant, 1998; Aldana‐Aranda *et al*., 2022). The relationship between a host and the sediment or substrate, the surrounding environmental conditions and the morphological features of the shell surface will all influence the presence and pattern of colonization of epizoic diatoms. The main diatoms found on molluscs fall into the ‘motile’ guild (Fig. 4), possibly reflecting selection on diatom ability to move to the best position on the host's surface (D'Alelio *et al*., 2010).

### (6) Diatoms on other basibionts

Other organisms have been studied, but much less frequently, with regard to the epizoic diatom species living on them. Echinoderms host species such as *Cocconeiopsis juandenovensis*, *Cocconeiopsis discoides* and *Cocconeiopsis cf. orthoneioides*. Epizoic diatoms found on Bryozoa included *Thalassiosira decipiens*, *Tabularia fasciculata*, *Rhoicosphenia marina*, *Rhoicosphenia genuflexa*, *Rhaphoneis surirella*, *Nitzschia valdestriata* and *Nitzschia navicularis* (Fig. 2E). On Porifera, *Fragilariopsis curta*, *Entomoneis paludosa*, *Druehlago lobbanii* and *Diploneis crabro* were found. For Annelida, *Achnanthes petersenii* and *Achnanthes longipes* were the most found species, together with several species of the genera *Nitzschia*, *Navicula* and *Amphora* (Fig. 2E). Lastly, several species of the genera *Licmophora*, *Grammatophora*, *Cocconeis*, *Campylodiscus*, *Amphora* and *Nitzschia* were found on Cnidaria (Fig. 2E; Table S2).

Again, the behaviour, location and morphology of the host organism will be key aspects in the initial establishment of the epibiosis (Di Camillo *et al*., 2005; Bavestrello *et al*., 2008). Diatoms can exhibit differential distribution across various areas of a host's body, depending on its morphological and physiological characteristics. Specifically, in Cnidaria, the distribution of diatom species on the surface of the host appears to be highly specific and varies among different body regions, with more motile and adnate forms tending to occupy the basal parts of the organism, whereas stalked (pedunculate) forms are predominantly found on the upper regions of the body (Di Camillo *et al*., 2005). Similarly, the dominant diatom species found on Echinodermata are closely associated with the complex surface structures of their hosts (Queiroz & Sales, 2023), as observed above for the microstructure of molluscan shells. In the case of hydroids, climatic and environmental conditions are known to play a crucial role in shaping diatom assemblages (Bavestrello *et al*., 2008). The relationship between substrate and host organism defines a remarkably similar dynamic of diatom colonization among benthic hosts, and the set of drivers will again affect patterns in diatom communities (Copp *et al*., 2009; Liuzzo *et al*., 2023; Tripp‐Valdez *et al*., 2023).

## V. IS THIS ‘COMMENSALISM’?

Although not yet fully understood, the potential benefits for diatoms inhabiting the surfaces of other organisms have been widely discussed in the literature (Tumlison & Clarke, 1996; Ziglar & Anderson, 2005; Majewska *et al*., 2015*a*). The relationship between diatoms and their basibionts is generally regarded as a specific form of commensalism, particularly in marine organisms (Ziglar & Anderson, 2005; Riaux‐Gobin *et al*., 2020). For Chordata, several advantages have been suggested for both the organisms involved (Fig. 6). For example, Ballantine, Navarro & Hensley (2001) argued that diatom colonization on the skin of redfish (*Scorpaena mystes*) facilitates host mimicry. This has been suggested for other organisms, including turtles and fish (Ziglar & Anderson, 2005; Majewska *et al*., 2015*a*,*b*; Falasco *et al*., 2025). By contrast, diatom colonization can affect predation behaviour (Akgul *et al*., 2014) by increasing water friction during swimming (Majewska *et al*., 2015*b*). Some researchers argue that the presence of diatoms on manatee (*Trichecus manatus*) skin may reduce dehydration but potentially increases the spread of pathogens (Majewska & Goosen, 2020). Hooper *et al*. (2019) found a positive correlation between the abundance of diatoms on the skin of Antarctic killer whales (*Orcinus orca*) and the abundance of *Tenacibaculum dicentrarchi*, known to be a pathogen in several fish species and generally associated with skin lesions.

**Fig. 6:**
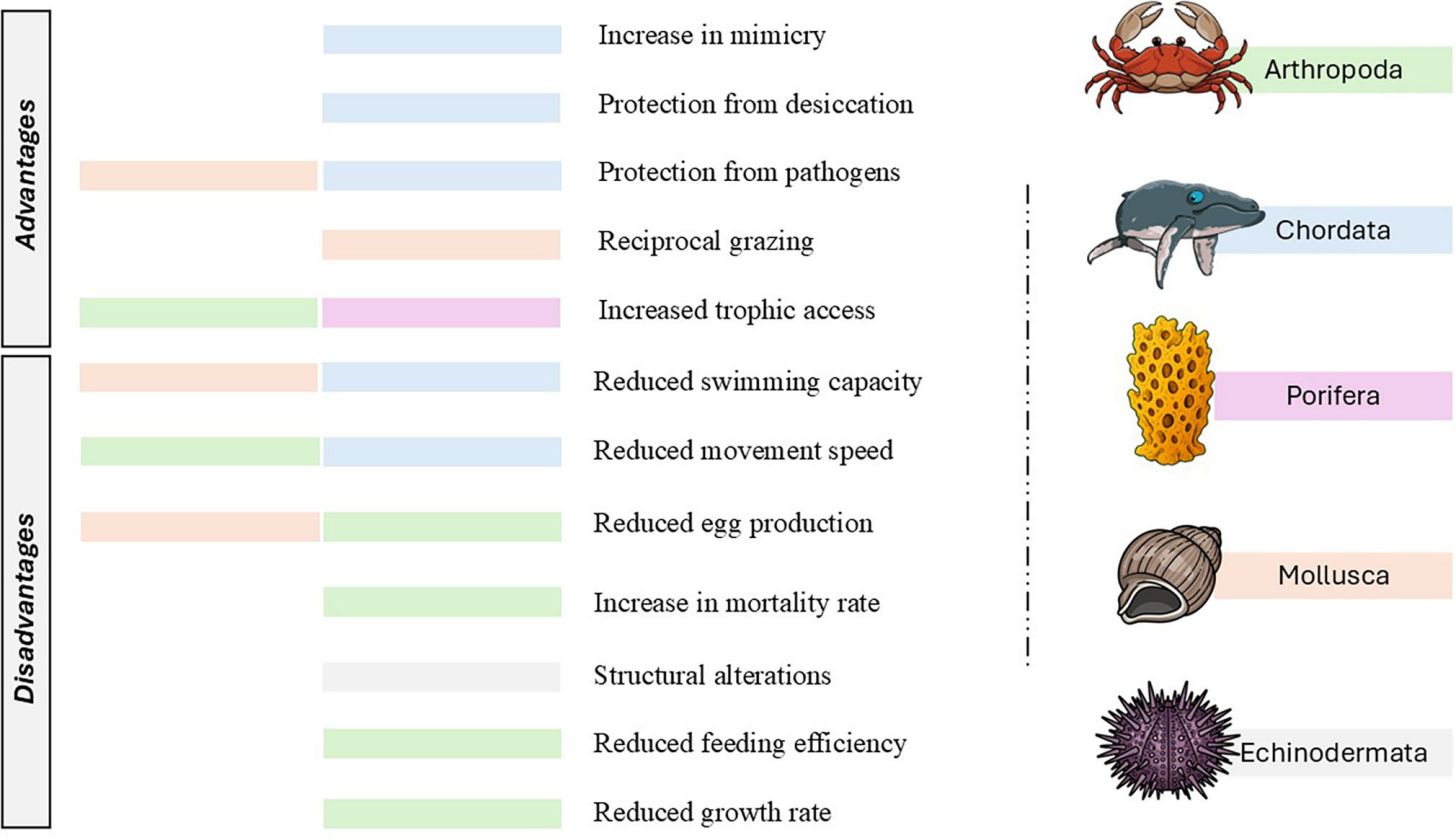
Advantages and disadvantages attributed in the literature to epizoic diatoms for each basibiont phylum.

Copepods are not believed to suffer disadvantages as a result of hosting epizoic diatoms (Lizárraga & Esqueda‐Escárcega, 2018). For instance, *Pseudodiaptomus annandalei* has been shown to benefit from the presence of diatoms (*Falcula hyalina*, *Haslea* sp. and *Tabularia* sp.) and can feed on them when necessary (Lin *et al*., 2025). In such cases, the presence of diatoms on the host's surface may be advantageous, providing reciprocal benefits. Conversely, other studies have shown that small arthropods heavily infested with diatoms may experience significant disadvantages (Pan *et al*., 2021). These include increased vulnerability to predators due to impaired mobility, altered feeding efficiency, changes in locomotion speed (Gárate‐Lizárraga & Muñetón‐Gómez, 2009), reduced egg production and growth rates, and elevated mortality. Notably, these negative effects often occur even at lower host population densities compared to control conditions without epibiosis and can affect the life cycle and survival rate of these organisms (Gaiser & Bachmann, 1993; Pan *et al*., 2021).

A high biomass of diatoms on mollusc shells can offer significant benefits, such as facilitating mutual grazing (Abbott & Bergey, 2007) and providing protection against pathogen attacks (Gillan & Cadée, 2000). Disadvantages also have been hypothesized for molluscs, particularly regarding reduced host mobility (Gillan & Cadée, 2000).

In the case of echinoderms, the presence of algae on their spines causes significant structural alterations, leading to decreased resistance and stability. These changes can directly impair the host's ability to defend itself and move effectively (Queiroz & Sales, 2023). For Cnidaria (Bavestrello *et al*., 2008; Romagnoli *et al*., 2014) and Porifera (Cerrano *et al*., 2004) some evidence suggests that the presence of microscopic algae on their surface could be advantageous. Some Porifera can incorporate them into the coanosome and reuse the silica of the frustules (Cerrano *et al*., 2004). Under specific conditions, Porifera can release specific chemical compounds to limit diatom growth, or to facilitate the growth of some species over others (Dobretsov, Dahms & Qian, 2005*a*; Dobretsov *et al*., 2005*b*). Similar findings have been reported for Antarctic ascidians (Koplovitz *et al*., 2011).

It is evident that diatoms benefit from their association with hosts, as this relationship offers a more effective means of dispersal compared to a free‐living lifestyle (Tumlison & Clarke, 1996; Ziglar & Anderson, 2005; Majewska *et al*., 2015*b*) or colonization of other substrates (Romagnoli *et al*., 2014). By colonizing living organisms, diatoms can access a wider range of environments and nutrient conditions, enhancing their ecological plasticity. From an evolutionary perspective, this strategy may offer selective advantages, promoting survival, colonization success, and resilience in fluctuating or competitive environments. Such advantages include a reduction in grazing pressure (González & Vergara, 1984; Abbott & Bergey, 2007; Gárate‐Lizárraga & Esqueda‐Escárcega, 2016), greater dispersal due to host displacement (Gárate‐Lizárraga & Esqueda‐Escárcega, 2016; Falasco *et al*., 2018), access to nutrients and CO_2_ derived from host metabolism (Abbott & Bergey, 2007; Padmakumar *et al*., 2015; Gárate‐Lizárraga & Esqueda‐Escárcega, 2016; Purushuothaman *et al*., 2021), and improved light exposure (Hanaoka, Ohtani & Ohtsuka, 2002; Cerrano *et al*., 2004; Abbott & Bergey, 2007; Torgan, Salomoni & Bicca, 2009; Lin *et al*., 2025).

It is plausible that viewing the relationship as commensalism is appropriate for larger organisms, where the size difference between host and epibiont minimizes any impacts on the basibiont. By contrast, in smaller organisms (e.g. planktonic taxa), the closer similarity in size may mean that diatoms significantly affect their host's life. Some authors regard diatom colonization on copepods as an ‘infestation’ (Li *et al*., 2020; Purushothaman *et al*., 2021; Bhowal *et al*., 2022; Pan *et al*., 2021), suggesting that there are no reciprocal advantages. In this case, the relationship could be considered as parasitism, defined by Solomon *et al*. (2015, p. 374) as ‘a relationship in which one of the participants (the parasite), either harms its host or in some sense lives at the expense of the host’. In conclusion, the relationship between diatoms and their hosts could be considered a complex mix of commensalism, parasitism and mutualism (‘a relationship in which two organisms of different species exist together with each one benefitting’; Solomon *et al*., 2015, p. 378) interactions, that may depend on the host, their physiological and behavioural responses to their epibionts, as well as on the climatic, ecological, and environmental conditions present during the establishment of the interaction.

## VI. ENVIRONMENTAL MONITORING USING EPIZOIC DIATOMS

The use of biotic substrates in environmental assessments was suggested by Vassal *et al*. (2021). However, as discussed above, many factors affect the diatom communities present on biotic surfaces, including the morphological and physiological features of the host (Majewska *et al*., 2017*a*; Donato‐Rondón *et al*., 2018; Roubeix *et al*., 2021), its behaviour (Rivera *et al*., 2018; Falasco *et al*., 2025) and the surrounding environmental conditions (Riaux‐Gobin *et al*., 2021; Roubeix *et al*., 2021; Vassal *et al*., 2021). We consider the following as limitations to the use of epibiont diatom communities in environmental assessment protocols.

First, basibionts show a wide range of dispersal and movement in water. For example, cetaceans can move tens of kilometres in a few days, and hundreds of metres in depth (Santos *et al*., 2019). This implies passage through a wide range of environmental conditions, in which their epizoic diatoms need to survive. Thus, epizoic diatoms exhibit different morphological and physiological adaptations in order to cope with adverse environmental conditions (Pavlov *et al*., 2016; Wu & Bergey, 2017). It is likely that the ability to overcome such conditions has played a stronger role in shaping diatom communities than the environmental conditions of one particular site.

Second, according to several studies (Denys & de Smet, 2010; Frankovich *et al*., 2015*a*; Majewska *et al*., 2017*b*; Majewska, 2021) diatom exchange between hosts requires direct contact between them, suggesting that there is a social component involved in the structure of epibiont diatom communities.

Third, a further aspect to consider is the presence of relationships between epibionts and basibionts. Although this aspect is still unclear (Ballantine *et al*., 2001; Ziglar & Anderson, 2005; Majewska *et al*., 2015*a*,*b*; Falasco *et al*., 2025), a mutualism between diatoms and their hosts could represent a significant factor shaping community structure that extends beyond purely environmental drivers. Such biotic interactions could play a crucial role in determining species composition, distribution patterns, and ecological dynamics within both marine and freshwater ecosystems.

Fourth, the presence of secretions and anti‐fouling substances released by basibionts may play a key role in shaping diatom community structure (Dobretsov *et al*., 2005*a*,*b*; Koplovitz *et al*., 2011; Majewska *et al*., 2023).

Finally, the use of organisms for environmental assessment may be challenging due to legal restrictions that impose regulatory requirements that necessitate additional permits or procedures. Such legal constraints can hinder the speed and efficiency of environmental analyses and limit the development of standardized sampling protocols, especially when protected or endangered species are involved.

## VII. CONCLUSIONS

(1) Most studies on epizoic diatoms have been conducted in the marine environment and on chordates, while freshwater sites and other taxa (e.g. Echinodermata, Annelida, Porifera, Bryozoa and Cnidaria) have been less represented. Future studies on epizoic diatoms should also focus on other basibionts, as well as on the freshwater environment, including by using molecular analyses.
(2) Diatoms observed on basibionts predominantly belong to the ‘motile’ and ‘high‐profile’ guilds, with ‘low‐profile’ and ‘planktonic’ guilds represented to a lesser extent. Initial diatom communities are related to their host's behaviour, ecological habits and interactions with both other organisms and substrates. Climatic and environmental conditions continue to shape community composition, acting as ‘drivers’.
(3) Interactions between diatoms and basibionts involve a complex mix of commensalism (especially between diatoms and Mollusca), mutualism (with Chordata) and parasitism (mainly in Arthropoda). The nature of the interaction will depend on the basibiont considered, and on its behaviour and ecological habits. The secretion of anti‐fouling substances by some basibionts could play a role in shaping diatom communities. The presence of consistent and stable communities among individuals strengthens the suggestion that morphological and behavioural features of hosts strongly influence the presence of epizoic taxa.
(4) Diatoms potentially represent useful organisms to explore aspects of host behaviour. In this context, the study of epizoic diatoms could shed light on host dispersal, social behaviour and habitat use at different scales (e.g. contact with the substrate and time spent outside the aquatic environment), as well as seasonal variations in these behaviours. In addition, evaluating colonization across different body regions can provide insights into the dynamics of host interactions, particularly in relation to community assembly in contact zones during mating events.
(5) The study of epizoic diatoms will enable a better understanding of their patterns of dispersal, which may be particularly useful regarding allochthonous diatom species and/or invasive species.
(6) Although still a preliminary hypothesis, the use of diatoms as a food source by copepods and sponges is worth investigating, potentially improving our understanding of the trophic chains involved.
(7) We outline several limitations regarding the use of epizoic diatoms in environmental monitoring and assessment, including movement of basibionts, contact between individuals and legislation constraints. These aspects currently limit the development of a single standardized sampling protocol.
(8) It is plausible that hosts facilitate the recovery of diatoms after extreme drought events, due to their use of refuge zones. The potential role of host animals in the resilience of their diatom communities remains to be investigated.

## Supporting information


**Data S1.** Dataset and complete list of references used for analyses.

**Fig. S1.** Graphical representation of the temporal distribution of studies conducted on epizoic diatoms (from 1920 to 2025) in all studied environments.
**Fig. S2**. Spatial distribution of the literature published on epizoic diatoms.
**Fig. S3**. Graphical representation of the number of studies conducted on the different phyla of basibiont, the epibiont diatom identification method employed, and the host environment considered.
**Fig. S4**. Number of diatom species found on different basibionts in (A) freshwater and (B) marine environments.
**Fig. S5**. Proportions of diatom size classes observed on the three main basibionts.
**Fig. S6**. Number of epibiont diatom genera found on different basibiont taxa, across all habitats.

**Table S1.** Summary of studies on epizoic diatoms included in this review.

**Table S2.** List of diatom taxa recorded on basibionts from 1920 to 2025.
