# Supplementary material for: Partners or passengers? Revisiting the association between diatoms and aquatic animals
Source: Biol Rev Camb Philos Soc. 2026 Jun 5;101(6):2597–612. doi: 10.1002/brv.70191 (PMC13736808; doi:10.1002/brv.70191)
Supplement: Supplementary file 1 [file BRV-101-2597-s002.docx]

**Table S2.** List of diatom taxa recorded on basibionts from 1920 to 2025. X = presence; – = absence. [1] indicates taxa explicitly considered as obligate epizoic in literature and the phylum with which they are strictly associated. [2] indicates diatom first described from epizoic samples, with the order and species of the basibiont on which an obligate (or presumed obligate) diatom was first recorded, with supporting references provided in the adjacent columns. N is the number of unique species present on that phylum. In the column “Other basibionts”, the basibiont(s) on which each diatom was found are indicated in parentheses. The first version of this table was created using ‘KableExtra’ and subsequently manually edited (‘KableExtra’; Zhu, 2021).

| Number |  | Environment | | Taxonomy | | |  |  |  |
| --- | --- | --- | --- | --- | --- | --- | --- | --- | --- |
|  | Species present | **Marine**  (*N* = 668) | **Freshwater**  (*N* = 450) | **Chordata**  (*N* = 356) | **Mollusca** (*N* = 159) | **Arthropoda** (*N* = 229) | **Other basibionts** | **Basibiont order (species) for obligate epizoic diatoms** | **Reference for obligate taxa** |
| 1 | *Achnanthes apiculata* (Greville) Riaux-Gobin, Compère, Hinz et Ector | ***X*** | ***–*** | ***X*** | ***–*** | ***–*** | ***X (Cnidaria)*** | **–** |  |
| 2 | *Achnanthes armillaris* (O.F.Müller) Guiry | ***X*** | ***–*** | ***–*** | ***X*** | ***–*** |  | ***–*** |  |
| 3 | *Achnanthes brevipes* C.Agardh | ***X*** | ***–*** | ***X*** | ***X*** | ***X*** | ***X (Cnidaria)*** | ***–*** |  |
| 4 | *Achnanthes brevipes var. angustata* (Greville) Cleve | ***X*** | ***–*** | ***–*** | ***–*** | ***X*** |  | ***–*** |  |
| 5 | *Achnanthes cf. adnata* Bory | ***X*** | ***–*** | ***–*** | ***X*** | ***X*** |  | ***–*** |  |
| 6 | *Achnanthes cf. groenlandica* (Cleve) Grunow | ***X*** | ***–*** | ***X*** | ***–*** | ***–*** |  | ***–*** |  |
| 7 | *Achnanthes cf. longipes* C.Agardh | ***X*** | ***–*** | ***–*** | ***–*** | ***–*** | ***X (Porifera)*** | ***–*** |  |
| 8 | *Achnanthes cf. pseudogroenlandica* Hendey | ***X*** | ***–*** | ***X*** | ***–*** | ***–*** |  | ***–*** |  |
| 9 | *Achnanthes elongata*^[1][2]^ Majewska et Van de Vijver | ***X*** | ***–*** | ***X*^[1]^** | ***–*** | ***–*** |  | **^[1][2]^Testudines (*Lepidochelys olivacea*)** | **Majewska *et al*. (2015*b*)** |
| 10 | *Achnanthes grunowii* Toyoda et D.M.Williams | ***X*** | ***–*** | ***–*** | ***X*** | ***–*** |  | ***–*** |  |
| 11 | *Achnanthes lemmermannii* Hustedt | ***X*** | ***–*** | ***–*** | ***X*** | ***–*** |  | ***–*** |  |
| 12 | *Achnanthes longipes* C.Agardh | ***X*** | ***–*** | ***X*** | ***X*** | ***–*** | ***X (Annelida)*** | ***–*** |  |
| 13 | *Achnanthes parvula* Kützing | ***X*** | ***–*** | ***–*** | ***X*** | ***–*** |  | ***–*** |  |
| 14 | *Achnanthes petersenii* Hustedt | ***X*** | ***–*** | ***–*** | ***–*** | ***–*** | ***X (Annelida)*** | ***–*** |  |
| 15 | *Achnanthes* spp*.* Bory | ***X*** | ***–*** | ***X*** | ***X*** | ***X*** | ***X (Bryozoa, Annelida)*** | ***–*** |  |
| 16 | *Achnanthes squaliformis*^[1][2]^ Majewska et Van de Vijver | ***X*** | ***–*** | ***X*^[1]^** | ***–*** | ***–*** |  | **^[1][2]^Testudines (*Lepidochelys olivacea*)** | **Majewska *et al*. (2015*b*)** |
| 17 | *Achnanthes yaquinensis* McIntire et Reimer | ***X*** | ***–*** | ***–*** | ***–*** | ***X*** |  | ***–*** |  |
| 18 | *Achnanthidium affine* (Grunow) Czarnecki | ***–*** | ***X*** | ***X*** | ***–*** | ***X*** |  | ***–*** |  |
| 19 | *Achnanthidium atomoides* Monnier, Lange-Bertalot et Ector | ***–*** | ***X*** | ***X*** | ***–*** | ***X*** |  | ***–*** |  |
| 20 | *Achnanthidium barbei* Le Cohu et Pérés | ***–*** | ***X*** | ***X*** | ***–*** | ***–*** |  | ***–*** |  |
| 21 | *Achnanthidium caledonicum* (Lange-Bertalot) Lange-Bertalot | ***–*** | ***X*** | ***X*** | ***–*** | ***–*** |  | ***–*** |  |
| 22 | *Achnanthidium delmontii* Pérés, le Cohu et Bartes | ***–*** | ***X*** | ***X*** | ***–*** | ***–*** |  | ***–*** |  |
| 23 | *Achnanthidium druartii* Rimet et Couté | ***–*** | ***X*** | ***X*** | ***–*** | ***–*** |  | ***–*** |  |
| 24 | *Achnanthidium eutrophilum* (Lange-Bertalot) Lange-Bertalot | ***–*** | ***X*** | ***X*** | ***–*** | ***X*** |  | ***–*** |  |
| 25 | *Achnanthidium gracillimum* (F.F.Meister) Lange-Bertalot | ***–*** | ***X*** | ***X*** | ***–*** | ***X*** |  | ***–*** |  |
| 26 | *Achnanthidium latecephalum* H.Kobayasi | ***–*** | ***X*** | ***–*** | ***–*** | ***X*** |  | ***–*** |  |
| 27 | *Achnanthidium lineare* W.Smith | ***–*** | ***X*** | ***X*** | ***–*** | ***X*** |  | ***–*** |  |
| 28 | *Achnanthidium minutissimum* (Kützing) Czarnecki | ***–*** | ***X*** | ***X*** | ***X*** | ***X*** |  | ***–*** |  |
| 29 | *Achnanthidium pyrenaicum* (Hustedt) H.Kobayasi | ***–*** | ***X*** | ***X*** | ***–*** | ***X*** |  | ***–*** |  |
| 30 | *Achnanthidium rivulare* Potapova et Ponader | ***–*** | ***X*** | ***X*** | ***–*** | ***–*** |  | ***–*** |  |
| 31 | *Achnanthidium* spp*.* Kützing | ***X*** | ***X*** | ***X*** | ***X*** | ***X*** |  | ***–*** |  |
| 32 | *Achnanthidium straubianum* (Lange-Bertalot) Lange-Bertalot | ***–*** | ***X*** | ***–*** | ***–*** | ***X*** |  | ***–*** |  |
| 33 | *Achnanthidium subatomus* (Hustedt) Lange-Bertalot | ***–*** | ***X*** | ***X*** | ***–*** | ***–*** |  | ***–*** |  |
| 34 | *Achnanthidium subhudsonis* (Hustedt) H.Kobayasi | ***–*** | ***X*** | ***X*** | ***–*** | ***–*** |  | ***–*** |  |
| 35 | *Actinella peronoides* Hustedt | ***–*** | ***X*** | ***X*** | ***–*** | ***–*** |  | ***–*** |  |
| 36 | *Actinella punctata* F.W.Lewis | ***–*** | ***X*** | ***X*** | ***–*** | ***–*** |  | ***–*** |  |
| 37 | *Actinella superperonoides* Metzeltin et Lange-Bertalot | ***–*** | ***X*** | ***X*** | ***–*** | ***–*** |  | ***–*** |  |
| 38 | *Actinoptychus senarius* (Ehrenberg) Ehrenberg | ***X*** | ***–*** | ***–*** | ***X*** | ***X*** |  | ***–*** |  |
| 39 | *Actinoptychus undulatus* (Kützing) Ralfs | ***X*** | ***–*** | ***–*** | ***–*** | ***–*** | ***X (Bryozoa)*** | ***–*** |  |
| 40 | *Adlafia minuscula* (Grunow) Lange-Bertalot | ***–*** | ***X*** | ***X*** | ***–*** | ***–*** |  | ***–*** |  |
| 41 | *Adlafia* spp*.* Moser, Lange-Bertalot et Metzeltin | ***–*** | ***X*** | ***X*** | ***–*** | ***–*** |  | ***–*** |  |
| 42 | *Amicula* spp*.* Witkowski et Lange-Bertalot | ***X*** | ***–*** | ***–*** | ***–*** | ***–*** | ***X (Echinodermata)*** | ***–*** |  |
| 43 | *Amphicocconeis* spp*.* De Stefano et Marino | ***X*** | ***–*** | ***–*** | ***–*** | ***–*** | ***X (Echinodermata)*** | ***–*** |  |
| 44 | *Amphipleura pellucida* Kützing | ***–*** | ***X*** | ***–*** | ***–*** | ***X*** |  | ***–*** |  |
| 45 | *Amphiprora hyalina* Greville | ***X*** | ***–*** | ***–*** | ***–*** | ***X*** |  | ***–*** |  |
| 46 | *Amphitetras subrotundata* Ehrenberg | ***X*** | ***–*** | ***X*** | ***–*** | ***–*** |  | ***–*** |  |
| 47 | *Amphora aff. crassa* W.Gregory | ***X*** | ***–*** | ***–*** | ***–*** | ***X*** |  | ***–*** |  |
| 48 | *Amphora aff. proteus* Ehrenberg | ***X*** | ***–*** | ***–*** | ***X*** | ***X*** |  | ***–*** |  |
| 49 | *Amphora aff. pusio* Cleve | ***X*** | ***–*** | ***–*** | ***X*** | ***X*** |  | ***–*** |  |
| 50 | *Amphora arcuata* A.W.F.Schmidt | ***X*** | ***–*** | ***X*** | ***–*** | ***–*** |  | ***–*** |  |
| 51 | *Amphora arcus* W.Gregory | ***X*** | ***–*** | ***X*** | ***–*** | ***–*** |  | ***–*** |  |
| 52 | *Amphora bigibba* Grunow | ***X*** | ***–*** | ***–*** | ***X*** | ***–*** |  | ***–*** |  |
| 53 | *Amphora castellata* Giffen | ***X*** | ***–*** | ***–*** | ***–*** | ***–*** | ***X (Annelida)*** | ***–*** |  |
| 54 | *Amphora cf. bigibba* Grunow | ***X*** | ***–*** | ***X*** | ***–*** | ***–*** |  | ***–*** |  |
| 55 | *Amphora cf. helenensis* Giffen | ***X*** | ***–*** | ***–*** | ***X*** | ***–*** |  | ***–*** |  |
| 56 | *Amphora cf. pediculus* (Kützing) Grunow | ***X*** | ***–*** | ***–*** | ***X*** | ***–*** |  | ***–*** |  |
| 57 | *Amphora cf. subacutiuscula* Schoeman | ***X*** | ***–*** | ***–*** | ***X*** | ***–*** |  | ***–*** |  |
| 58 | *Amphora cf. tenuissima* Giffen | ***X*** | ***–*** | ***–*** | ***X*** | ***–*** |  | ***–*** |  |
| 59 | *Amphora cingulata* Cleve | ***X*** | ***–*** | ***–*** | ***–*** | ***X*** |  | ***–*** |  |
| 60 | *Amphora coffeaeformis* (C.Agardh) Kützing | ***X*** | ***–*** | ***–*** | ***–*** | ***X*** | ***X (Cnidaria, Annelida, Bryozoa)*** | ***–*** |  |
| 61 | *Amphora commutata* Grunow | ***–*** | ***–*** | ***–*** | ***X*** | ***–*** |  | ***–*** |  |
| 62 | *Amphora compacta* Mann | ***X*** | ***–*** | ***X*** | ***–*** | ***–*** |  | ***–*** |  |
| 63 | *Amphora copulata* (Kützing) Schoeman et Archibald | ***–*** | ***X*** | ***X*** | ***–*** | ***X*** |  | ***–*** |  |
| 64 | *Amphora cymbamphora* Cholnoky | ***X*** | ***–*** | ***X*** | ***–*** | ***–*** |  | ***–*** |  |
| 65 | *Amphora formosa var. studeri* (Janisch) Peragallo et Peragallo | ***X*** | ***–*** | ***X*** | ***–*** | ***–*** |  | ***–*** |  |
| 66 | *Amphora granulata* W.Gregory | ***–*** | ***X*** | ***X*** | ***–*** | ***–*** |  | ***–*** |  |
| 67 | *Amphora helenensis* Giffen | ***X*** | ***–*** | ***X*** | ***–*** | ***X*** |  | ***–*** |  |
| 68 | *Amphora holsaticoides* Nagumo et H.Kobayasi | ***X*** | ***–*** | ***–*** | ***–*** | ***X*** |  | ***–*** |  |
| 69 | *Amphora hyalina* Kützing | ***X*** | ***–*** | ***–*** | ***–*** | ***X*** |  | ***–*** |  |
| 70 | *Amphora japonica* F.Meister | ***X*** | ***–*** | ***–*** | ***X*** | ***–*** |  | ***–*** |  |
| 71 | *Amphora lanceolata* Ehrenberg | ***X*** | ***–*** | ***–*** | ***X*** | ***–*** |  | ***–*** |  |
| 72 | *Amphora longiceps* Simonsen | ***X*** | ***–*** | ***–*** | ***X*** | ***–*** |  | ***–*** |  |
| 73 | *Amphora macedoniensis* Nagumo | ***–*** | ***X*** | ***X*** | ***–*** | ***–*** |  | ***–*** |  |
| 74 | *Amphora marina* Cleve | ***X*** | ***–*** | ***–*** | ***–*** | ***X*** | ***X (Bryozoa)*** | ***–*** |  |
| 75 | *Amphora meridionalis* Levkov | ***–*** | ***X*** | ***X*** | ***–*** | ***–*** |  | ***–*** |  |
| 76 | *Amphora normanii* Rabenhorst | ***–*** | ***X*** | ***X*** | ***–*** | ***–*** |  | ***–*** |  |
| 77 | *Amphora ovalis* (Kützing) Kützing | ***X*** | ***X*** | ***X*** | ***X*** | ***X*** |  | ***–*** |  |
| 78 | *Amphora pediculus* (Kützing) Grunow | ***X*** | ***X*** | ***X*** | ***–*** | ***X*** | ***X (Cnidaria)*** | ***–*** |  |
| 79 | *Amphora proteus var. contigua* (W.Gregory) Cleve | ***X*** | ***–*** | ***–*** | ***–*** | ***X*** |  | ***–*** |  |
| 80 | *Amphora proteus var. proteus* W.Gregory | ***X*** | ***–*** | ***–*** | ***–*** | ***X*** |  | ***–*** |  |
| 81 | *Amphora pusio* Cleve | ***X*** | ***–*** | ***–*** | ***–*** | ***–*** | ***X (Bryozoa)*** | ***–*** |  |
| 82 | *Amphora* spp*.* (Kützing) Ehrenberg | ***X*** | ***X*** | ***X*** | ***X*** | ***X*** | ***X (Bryozoa, Cnidaria)*** | ***–*** |  |
| 83 | *Amphora tenerrima* Aleem et Hustedt | ***X*** | ***–*** | ***–*** | ***–*** | ***–*** | ***X (Annelida, Bryozoa)*** | ***–*** |  |
| 84 | *Aneumastus stroesei* (Østrup) D.G. Mann et Stickle | ***–*** | ***X*** | ***X*** | ***–*** | ***X*** |  | ***–*** |  |
| 85 | *Anorthoneis* spp*.* Grunow | ***X*** | ***–*** | ***–*** | ***–*** | ***–*** | ***X (Echinodermata)*** | ***–*** |  |
| 86 | *Ardissonea crystallina* (C.Agardh) Grunow | ***X*** | ***–*** | ***–*** | ***X*** | ***–*** |  | ***–*** |  |
| 87 | *Ardissonea fulgens* (Greville) Grunow | ***X*** | ***–*** | ***–*** | ***–*** | ***–*** | ***X (Cnidaria)*** | ***–*** |  |
| 88 | *Ardissonea fulgens var. gigantea* (Lobarzewsky) De Toni | ***X*** | ***–*** | ***X*** | ***–*** | ***–*** |  | ***–*** |  |
| 89 | *Astartiella cf. bahusiensis* (Grunow) Witkowski, Lange-Bertalot et Metzeltin | ***X*** | ***–*** | ***–*** | ***X*** | ***–*** |  | ***–*** |  |
| 90 | *Astartiella* spp*.* Witkowski, Lange-Bertalot et Metzeltin | ***X*** | ***–*** | ***–*** | ***–*** | ***–*** | ***X (Echinodermata)*** | ***–*** |  |
| 91 | *Asterionella* spp*.* Hassall | ***X*** | ***X*** | ***–*** | ***X*** | ***X*** |  | ***–*** |  |
| 92 | *Aulacoseira granulata* (Ehrenberg) Simonsen | ***–*** | ***X*** | ***X*** | ***–*** | ***–*** |  | ***–*** |  |
| 93 | *Aulacoseira pusilla* (F.Meister) Tuji et Houki | ***–*** | ***X*** | ***X*** | ***–*** | ***–*** |  | ***–*** |  |
| 94 | *Aulacoseira* spp*.* Thwaites | ***–*** | ***X*** | ***X*** | ***X*** | ***–*** |  | ***–*** |  |
| 95 | *Azpeitia nodulifera* (A.W.F.Schmidt) G.A.Fryxell et P.A.Sims | ***X*** | ***–*** | ***X*** | ***X*** | ***–*** |  | ***–*** |  |
| 96 | *Azpeitia* spp*.* Peragallo | ***X*** | ***–*** | ***X*** | ***–*** | ***–*** |  | ***–*** |  |
| 97 | *Bacillaria paradoxa* J.F.Gmelin | ***X*** | ***–*** | ***–*** | ***–*** | ***X*** |  | ***–*** |  |
| 98 | *Bacillaria paxillifera* (O.F.Müller) Hendey | ***–*** | ***X*** | ***X*** | ***–*** | ***–*** |  | ***–*** |  |
| 99 | *Bacillaria socialis var. kariana* (Grunow) Grunow | ***X*** | ***–*** | ***–*** | ***X*** | ***X*** |  | ***–*** |  |
| 100 | *Bacillaria* spp*.* J.F.Gmelin | ***X*** | ***–*** | ***–*** | ***X*** | ***X*** |  | ***–*** |  |
| 101 | *Bennettella ceticola*^[1][2]^ (E.W.Nelson) R.W.Holmes | ***X*** | ***–*** | ***X*^[1]^** | ***–*** | ***–*** |  | **^[1][2]^Artiodactyla (*Balenoptera musculus*; *Balenoptera physalyus*)** | **Holmes (1985)** |
| 102 | *Bennettella ceticola f. berardiusii*^[1]^ (E.W.Nelson) R.W.Holmes | ***X*** | ***–*** | ***X*^[1]^** | ***–*** | ***–*** |  | **^[1][2]^Artiodactyla (*Balenoptera musculus*; *Balenoptera physalus*)** | **Holmes (1985)** |
| 103 | *Bennettella constricta*^[1][2]^ (Nemoto) R.W.Holmes | ***X*** | ***–*** | ***X*^[1]^** | ***–*** | ***–*** |  | **^[1][2]^Artiodactyla (*Balenoptera musculus*; *Balenoptera physalus*)** | **Holmes (1985) (*Cocconeis ceticola form. constricta* by Nemoto, 1956)** |
| 104 | *Berkeleya cf. fennica* Juhlin-Dannfelt | ***–*** | ***–*** | ***X*** | ***–*** | ***–*** |  | ***–*** |  |
| 105 | *Berkeleya rutilans* (Trentepohl) Grunow | ***X*** | ***–*** | ***–*** | ***X*** | ***–*** | ***X (Cnidaria)*** | ***–*** |  |
| 106 | *Berkeleya* spp*.* Greville | ***X*** | ***–*** | ***X*** | ***–*** | ***–*** |  | ***–*** |  |
| 107 | *Biddulphia alternans* (Bailey) Van Heurck | ***X*** | ***–*** | ***–*** | ***–*** | ***X*** | ***X (Bryozoa)*** | ***–*** |  |
| 108 | *Biddulphia aurita* (Lyngbye) Brébisson et Godey | ***X*** | ***–*** | ***X*** | ***–*** | ***–*** |  | ***–*** |  |
| 109 | *Biddulphia biddulphia* (J.E.Smith) Boyer | ***X*** | ***–*** | ***–*** | ***X*** | ***–*** |  | ***–*** |  |
| 110 | *Biddulphia californica* (A.W.F.Schmidt) Wolle | ***X*** | ***–*** | ***–*** | ***–*** | ***X*** |  | ***–*** |  |
| 111 | *Biddulphia juncta* (A.W.F.Schmidt) A.Mann | ***X*** | ***–*** | ***X*** | ***–*** | ***–*** |  | ***–*** |  |
| 112 | *Biddulphia mobiliensis* (Bailey) Grunow | ***X*** | ***–*** | ***X*** | ***–*** | ***–*** |  | ***–*** |  |
| 113 | *Biddulphia* spp*.* Gray | ***X*** | ***–*** | ***–*** | ***X*** | ***X*** | ***X (Porifera)*** | ***–*** |  |
| 114 | *Biddulphiopsis membranacea* Cleve | ***X*** | ***–*** | ***X*** | ***–*** | ***–*** |  | ***–*** |  |
| 115 | *Biremis lucens* (Hustedt) Sabbe, Witkowskiet Vyverman | ***X*** | ***–*** | ***–*** | ***X*** | ***–*** |  | ***–*** |  |
| 116 | *Biremis ridicula* (Giffen) D.G.Mann | ***X*** | ***–*** | ***–*** | ***–*** | ***X*** |  | ***–*** |  |
| 117 | *Brachysira aponina* Kützing | ***X*** | ***–*** | ***X*** | ***X*** | ***–*** |  | ***–*** |  |
| 118 | *Brachysira cf. estonarium* Witkowski, Lange-Bertalot et Metzeltin | ***X*** | ***–*** | ***–*** | ***–*** | ***X*** |  | ***–*** |  |
| 119 | *Brachysira estonarium* Witkowski, Lange-Bertalot et Metzeltin | ***X*** | ***–*** | ***X*** | ***–*** | ***–*** |  | ***–*** |  |
| 120 | *Brachysira neglectissima* Lange-Bertalot | ***–*** | ***X*** | ***X*** | ***–*** | ***–*** |  | ***–*** |  |
| 121 | *Brachysira neoexilis* Lange-Bertalot | ***–*** | ***X*** | ***X*** | ***–*** | ***X*** |  | ***–*** |  |
| 122 | *Brachysira serians* (Brébisson) Round et D.G.Mann | ***–*** | ***X*** | ***X*** | ***–*** | ***–*** |  | ***–*** |  |
| 123 | *Caloneis alpestris* (Grunow) Cleve | ***X*** | ***–*** | ***–*** | ***–*** | ***–*** | ***X (Cnidaria)*** | ***–*** |  |
| 124 | *Caloneis amphisbaena* (Bory) Cleve | ***–*** | ***X*** | ***X*** | ***–*** | ***–*** |  | ***–*** |  |
| 125 | *Caloneis cf. linearis* (Grunow) Boyer | ***X*** | ***–*** | ***–*** | ***X*** | ***–*** |  | ***–*** |  |
| 126 | *Caloneis egena* (A.W.F.Schmidt) Cleve | ***X*** | ***–*** | ***X*** | ***–*** | ***–*** |  | ***–*** |  |
| 127 | *Caloneis fontinalis* (Grunow) A.Cleve | ***–*** | ***X*** | ***X*** | ***–*** | ***X*** |  | ***–*** |  |
| 128 | *Caloneis lancettula* (P.Schulz) Lange-Bertalot et Witkowski | ***–*** | ***X*** | ***X*** | ***–*** | ***X*** |  | ***–*** |  |
| 129 | *Caloneis liber* (W.Smith) Cleve | ***X*** | ***–*** | ***X*** | ***–*** | ***–*** |  | ***–*** |  |
| 130 | *Caloneis liber var. umbilicata* (Grunow) Cleve | ***X*** | ***–*** | ***X*** | ***–*** | ***–*** |  | ***–*** |  |
| 131 | *Caloneis linearis* (Grunow) Boyer | ***X*** | ***–*** | ***–*** | ***X*** | ***X*** |  | ***–*** |  |
| 132 | *Caloneis* spp*.* Cleve | ***X*** | ***X*** | ***X*** | ***X*** | ***X*** |  | ***–*** |  |
| 133 | *Caloneis westii* (W.Smith) Hendey | ***X*** | ***–*** | ***–*** | ***X*** | ***–*** |  | ***–*** |  |
| 134 | *Campylodiscus cf. clevei* Peragallo et Peragallo | ***X*** | ***–*** | ***–*** | ***–*** | ***–*** | ***X (Cnidaria)*** | ***–*** |  |
| 135 | *Campylodiscus cf. clypeus*^[1]^ (Ehrenberg) Ehrenberg | ***X*** | ***–*** | ***X*^[1]^** | ***–*** | ***–*** |  | **^[1]^Sirenia (*Trichecus manatus*)** | **Violante-Huerta *et al*. (2018)** |
| 136 | *Campylodiscus cf. decorus* Brébisson | ***X*** | ***–*** | ***–*** | ***–*** | ***–*** | ***X (Cnidaria)*** | ***–*** |  |
| 137 | *Campylodiscus cf. thuretii* Brébisson | ***X*** | ***–*** | ***–*** | ***–*** | ***–*** | ***X (Cnidaria)*** | ***–*** |  |
| 138 | *Campylodiscus decorus var. pinnatus* (H.Peragallo) De Toni | ***X*** | ***–*** | ***–*** | ***–*** | ***–*** | ***X (Cnidaria)*** | ***–*** |  |
| 139 | *Campylodiscus fastuosus* Ehrenberg | ***X*** | ***–*** | ***–*** | ***–*** | ***–*** | ***X (Cnidaria)*** | ***–*** |  |
| 140 | *Campylodiscus subangularis* Cleve et Mӧller | ***X*** | ***–*** | ***–*** | ***–*** | ***X*** |  | ***–*** |  |
| 141 | *Campyloneis grevillei* (W.Smith) Grunow | ***X*** | ***–*** | ***–*** | ***X*** | ***–*** |  | ***–*** |  |
| 142 | *Campylopyxis garkeana* (Grunow) Medlin | ***X*** | ***–*** | ***–*** | ***X*** | ***–*** |  | ***–*** |  |
| 143 | *Catenula adhaerens* (Mereschkowsky) Mereschkowsky | ***X*** | ***–*** | ***–*** | ***X*** | ***–*** |  | ***–*** |  |
| 144 | *Cavinula variostriata* (Krasske) D.G.Mann et Stickle | ***–*** | ***X*** | ***–*** | ***X*** | ***–*** |  | ***–*** |  |
| 145 | *Ceratanaulus alaris* Gorecka, Witkowski, Dabek et Ashworth | ***X*** | ***–*** | ***–*** | ***X*** | ***–*** |  | ***–*** |  |
| 146 | *Cerataulina* spp*.* Peragallo | ***X*** | ***–*** | ***–*** | ***–*** | ***X*** |  | ***–*** |  |
| 147 | *Chaetoceros atlanticus* Cleve | ***X*** | ***–*** | ***X*** | ***–*** | ***–*** |  | ***–*** |  |
| 148 | *Chaetoceros concavicornis* Mangin | ***X*** | ***–*** | ***X*** | ***–*** | ***–*** |  | ***–*** |  |
| 149 | *Chaetoceros densus* (Cleve) Cleve | ***X*** | ***–*** | ***X*** | ***–*** | ***–*** |  | ***–*** |  |
| 150 | *Chaetoceros* spp*.* Ehrenberg | ***X*** | ***–*** | ***–*** | ***X*** | ***X*** |  | ***–*** |  |
| 151 | *Chamaepinnularia braisilianopsis* Metzeltin et Lange-Bertalot | ***–*** | ***X*** | ***X*** | ***–*** | ***–*** |  | ***–*** |  |
| 152 | *Chamaepinnularia muscicola* (J.B.Petersen) Kulikovskiy, Lange-Bertalot et A.Witkowski | ***–*** | ***X*** | ***X*** | ***–*** | ***–*** |  | ***–*** |  |
| 153 | *Chamaepinnularia submuscicola* (Krasske) Lange-Bertalot | ***–*** | ***X*** | ***X*** | ***–*** | ***–*** |  | ***–*** |  |
| 154 | *Chelonicola caribeana*^[1][2]^ Riaux-Gobin, Witkowski, Ector et Chevallier | ***X*** | ***–*** | ***X*^[1]^** | ***–*** | ***–*** |  | **^[1][2]^Testudines (*Chelonia mydas*)** | **Riaux-Gobin *et al*. (2017*b*)** |
| 155 | *Chelonicola costaricensis*^[1][2]^ Majewska, De Stefano et Van de Vijver | ***X*** | ***–*** | ***X*^[1]^** | ***–*** | ***–*** |  | **^[1][2]^Testudines (*Lepidochelys olivacea*)** | **Majewska *et al*. (2015*b*)** |
| 156 | *Chelonicola* spp.*^.^*^[1][2]^ Majewska, De Stefano et Van de Vijver | ***X*** | ***–*** | ***X*^[1]^** | ***–*** | ***–*** |  | **^[1][2]^Testudines** | **Majewska *et al*. (2015*b*)** |
| 157 | *Climacodium* sp. Grunow | ***X*** | ***–*** | ***–*** | ***–*** | ***X*** |  | ***–*** |  |
| 158 | *Climaconeis inflexa* (Brébisson) E.J.Cox | ***X*** | ***–*** | ***–*** | ***X*** | ***–*** |  | ***–*** |  |
| 159 | *Cocconeiopsis cf. orthoneioides* (Hustedt) Witkowski, Lange-Bertalot et Metzeltin | ***X*** | ***–*** | ***–*** | ***–*** | ***–*** | ***X (Echinodermata)*** | ***–*** |  |
| 160 | *Cocconeiopsis discoides* Riaux-Gobin et Witkowski | ***X*** | ***–*** | ***–*** | ***–*** | ***–*** | ***X (Echinodermata)*** | ***–*** |  |
| 161 | *Cocconeiopsis juandenovensis* Riaux-Gobin et Witkowski | ***X*** | ***–*** | ***–*** | ***–*** | ***–*** | ***X (Echinodermata)*** | ***–*** |  |
| 162 | *Cocconeiopsis* spp*.* Witkowski, Lange-Bertalot et Metzeltin | ***X*** | ***–*** | ***–*** | ***–*** | ***–*** | ***X (Echinodermata)*** | ***–*** |  |
| 163 | *Cocconeis cf. californica* Grunow | ***X*** | ***–*** | ***–*** | ***–*** | ***X*** |  | ***–*** |  |
| 164 | *Cocconeis cf. disculus* (Schumann) Cleve | ***X*** | ***–*** | ***–*** | ***–*** | ***X*** |  | ***–*** |  |
| 165 | *Cocconeis cf. euglypta* Ehrenberg | ***X*** | ***–*** | ***–*** | ***–*** | ***X*** |  | ***–*** |  |
| 166 | *Cocconeis cf. neothumensis* Krammer | ***X*** | ***–*** | ***X*** | ***–*** | ***–*** |  | ***–*** |  |
| 167 | *Cocconeis cf. neothumensis var. marina* M.De Stefano, D.Marino et L.Mazella | ***X*** | ***–*** | ***X*** | ***–*** | ***–*** |  | ***–*** |  |
| 168 | *Cocconeis cf. patagonica* Riaux-Gobin et Cefarelli | ***X*** | ***–*** | ***–*** | ***–*** | ***X*** |  | ***–*** |  |
| 169 | *Cocconeis cf. pseudolineata* Ehrenberg | ***X*** | ***–*** | ***–*** | ***–*** | ***X*** |  | ***–*** |  |
| 170 | *Cocconeis convexa* Giffen | ***X*** | ***–*** | ***–*** | ***–*** | ***X*** |  | ***–*** |  |
| 171 | *Cocconeis costata* W.Gregory | ***X*** | ***–*** | ***X*** | ***–*** | ***–*** |  | ***–*** |  |
| 172 | *Cocconeis danica* Flӧgel | ***X*** | ***–*** | ***–*** | ***–*** | ***X*** |  | ***–*** |  |
| 173 | *Cocconeis dirupta* W.Gregory | ***X*** | ***–*** | ***–*** | ***–*** | ***X*** | ***X (Cnidaria)*** | ***–*** |  |
| 174 | *Cocconeis distans* W.Gregory | ***X*** | ***–*** | ***–*** | ***X*** | ***–*** |  | ***–*** |  |
| 175 | *Cocconeis euglypta* Ehrenberg | ***–*** | ***X*** | ***X*** | ***X*** | ***X*** |  | ***–*** |  |
| 176 | *Cocconeis fluviatilis* J.H.Wallace | ***–*** | ***X*** | ***–*** | ***X*** | ***–*** |  | ***–*** |  |
| 177 | *Cocconeis guttata* Hustedt et Aleem | ***X*** | ***–*** | ***–*** | ***X*** | ***–*** |  | ***–*** |  |
| 178 | *Cocconeis latecostata* Hustedt | ***X*** | ***–*** | ***X*** | ***–*** | ***–*** |  | ***–*** |  |
| 179 | *Cocconeis lineata* Ehrenberg | ***X*** | ***X*** | ***X*** | ***X*** | ***X*** | ***X (Cnidaria)*** | ***–*** |  |
| 180 | *Cocconeis molesta* Kützing | ***X*** | ***–*** | ***–*** | ***–*** | ***–*** | ***X (Bryozoa)*** | ***–*** |  |
| 181 | *Cocconeis neothumensis* Ehrenberg | ***X*** | ***–*** | ***–*** | ***X*** | ***–*** | ***X (Cnidaria)*** | ***–*** |  |
| 182 | *Cocconeis neothumensis var. marina* M.De Stefano, D.Marino et L.Mazella | ***X*** | ***–*** | ***X*** | ***–*** | ***–*** | ***X (Cnidaria)*** | ***–*** |  |
| 183 | *Cocconeis notata* Petit | ***X*** | ***–*** | ***–*** | ***–*** | ***–*** | ***X (Cnidaria)*** | ***–*** |  |
| 184 | *Cocconeis patagonica* Riaux-Gobin et Cefarelli | ***X*** | ***–*** | ***–*** | ***–*** | ***X*** |  | ***–*** |  |
| 185 | *Cocconeis pediculus* Ehrenberg | ***X*** | ***X*** | ***X*** | ***X*** | ***X*** |  | ***–*** |  |
| 186 | *Cocconeis pelta* A.W.F.Schmidt | ***X*** | ***–*** | ***–*** | ***–*** | ***X*** |  | ***–*** |  |
| 187 | *Cocconeis peltoides* Hustedt | ***X*** | ***–*** | ***–*** | ***X*** | ***–*** |  | ***–*** |  |
| 188 | *Cocconeis placentula* Ehrenberg | ***X*** | ***X*** | ***X*** | ***X*** | ***X*** |  | ***–*** |  |
| 189 | *Cocconeis pseudodiruptoides* Foged | ***X*** | ***–*** | ***–*** | ***–*** | ***X*** |  | ***–*** |  |
| 190 | *Cocconeis pseudolineata* (Geitler) Lange-Bertalot | ***–*** | ***X*** | ***X*** | ***–*** | ***X*** |  | ***–*** |  |
| 191 | *Cocconeis pseudomarginata* W.Gregory | ***X*** | ***–*** | ***–*** | ***–*** | ***X*** |  | ***–*** |  |
| 192 | *Cocconeis pseudonotata* M.De Stefano et D.Marino | ***X*** | ***–*** | ***–*** | ***–*** | ***–*** | ***X (Cnidaria)*** | ***–*** |  |
| 193 | *Cocconeis scutellum* Ehrenberg | ***X*** | ***–*** | ***–*** | ***X*** | ***X*** | ***X (Bryozoa, Cnidaria)*** | ***–*** |  |
| 194 | *Cocconeis* spp*.* Ehrenberg | ***X*** | ***X*** | ***X*** | ***X*** | ***X*** | ***X (Bryozoa, Cnidaria)*** | ***–*** |  |
| 195 | *Cocconeis stauroneiformis* H.Okuno | ***X*** | ***–*** | ***–*** | ***–*** | ***X*** | ***X (Bryozoa, Cnidaria)*** | ***–*** |  |
| 196 | *Coronia ambigua* (Greville) Ruck et Guiry | ***X*** | ***–*** | ***–*** | ***–*** | ***X*** |  | ***–*** |  |
| 197 | *Coronia decora* (Brébisson) Ruck et Guiry | ***X*** | ***–*** | ***X*** | ***–*** | ***–*** |  | ***–*** |  |
| 198 | *Coscinodiscus angstii* H.H.Gran | ***X*** | ***–*** | ***X*** | ***–*** | ***–*** |  | ***–*** |  |
| 199 | *Coscinodiscus asteromphalus* Ehrenberg | ***X*** | ***–*** | ***X*** | ***–*** | ***–*** |  | ***–*** |  |
| 200 | *Coscinodiscus kuetzingii* A.W.F.Schmidt | ***X*** | ***–*** | ***X*** | ***–*** | ***–*** |  | ***–*** |  |
| 201 | *Coscinodiscus lineatus* Ehrenberg | ***X*** | ***–*** | ***X*** | ***–*** | ***–*** |  | ***–*** |  |
| 202 | *Coscinodiscus megalomma* A.W.F.Schmidt | ***X*** | ***–*** | ***–*** | ***X*** | ***–*** |  | ***–*** |  |
| 203 | *Coscinodiscus oculus-iridis* Ehrenberg | ***X*** | ***–*** | ***–*** | ***X*** | ***–*** |  | ***–*** | **–** |
| 204 | *Coscinodiscus radiatus* Ehrenberg | ***X*** | ***–*** | ***X*** | ***–*** | ***–*** | ***X (Bryozoa)*** | ***–*** |  |
| 205 | *Coscinodiscus* spp*.* Ehrenberg | ***X*** | ***–*** | ***–*** | ***X*** | ***X*** |  | ***–*** |  |
| 206 | *Coscinodiscus wailesii* Gran et Angst | ***X*** | ***–*** | ***X*** | ***–*** | ***–*** |  | ***–*** |  |
| 207 | *Cosmioneis pusilla* (W.Smith) D.G.Mann et Stickle | ***–*** | ***X*** | ***–*** | ***X*** | ***–*** |  | ***–*** |  |
| 208 | *Craspedostauros alatus*^[1][2]^ Majewska et M.A.Ashworth | ***X*** | ***–*** | ***X*^[1]^** | ***–*** | ***–*** |  | **^[2]^Testudines (*Caretta caretta)*** |  |
| 209 | *Craspedostauros danayanus*^[1][2]^ Majewska et M.A.Ashworth | ***X*** | ***–*** | ***X*^[1]^** | ***–*** | ***–*** |  | **^[2]^Testudines (*Dermochelys coriacea*)** | **Majewska *et al*. (2020)** |
| 210 | *Craspedostauros legouvelloanus*^[1][2]^ Majewska et Bosak | ***X*** | ***–*** | ***X*^[1]^** | ***–*** | ***–*** |  | **^[2]^Testudines (*Caretta caretta*)** | **Majewska *et al*. (2020)** |
| 211 | *Craspedostauros macewanii*^[1][2]^ Majewska et M.A.Ashworth | ***X*** | ***–*** | ***X*^[1]^** | ***–*** | ***–*** |  | **^[2]^Testudines (*Caretta caretta*)** | **Majewska *et al*. (2020)** |
| 212 | *Craspedostauros* spp*.* Cox | ***X*** | ***–*** | ***X*** | ***X*** | ***–*** |  | ***–*** |  |
| 213 | *Craticula cuspidata* (Kützing) D.G.Mann | ***–*** | ***X*** | ***X*** | ***–*** | ***–*** |  | ***–*** |  |
| 214 | *Craticula subminuscula* (Manguin) C.E.Wetzel et Ector | ***–*** | ***X*** | ***X*** | ***–*** | ***–*** |  | ***–*** |  |
| 215 | *Ctenophora pulchella* (Kützing) D.M.Williams et Round | ***–*** | ***X*** | ***X*** | ***–*** | ***–*** |  | ***–*** |  |
| 216 | *Cyclophora* spp*.* Castracane | ***X*** | ***–*** | ***X*** | ***X*** | ***–*** |  | ***–*** |  |
| 217 | *Cyclophora tenuis* Castracane | ***X*** | ***–*** | ***X*** | ***X*** | ***–*** | ***X (Cnidaria)*** | ***–*** |  |
| 218 | *Cyclostephanos dubius* (Fricke) Round | ***X*** | ***–*** | ***–*** | ***X*** | ***–*** |  | ***–*** |  |
| 219 | *Cyclotella atomus* Hustedt | ***–*** | ***X*** | ***X*** | ***–*** | ***–*** |  | ***–*** |  |
| 220 | *Cyclotella choctawhatcheeana* Prasad | ***X*** | ***–*** | ***–*** | ***X*** | ***–*** |  | ***–*** |  |
| 221 | *Cyclotella distinguenda* Hustedt | ***–*** | ***X*** | ***X*** | ***–*** | ***–*** |  | ***–*** |  |
| 222 | *Cyclotella glomerata* H.Bachmann | ***X*** | ***–*** | ***–*** | ***–*** | ***X*** |  | ***–*** |  |
| 223 | *Cyclotella litoralis* Lange et Syvertsen | ***X*** | ***–*** | ***–*** | ***–*** | ***–*** | ***X (Bryozoa)*** | ***–*** |  |
| 224 | *Cyclotella meneghiniana* Kützing | ***X*** | ***X*** | ***X*** | ***X*** | ***X*** |  | ***–*** |  |
| 225 | *Cyclotella ocellata* Pantocsek | ***–*** | ***X*** | ***–*** | ***–*** | ***X*** |  | ***–*** |  |
| 226 | *Cyclotella* spp*.* Kützing | ***X*** | ***X*** | ***–*** | ***X*** | ***X*** |  | ***–*** |  |
| 227 | *Cylindrotheca closterium* (Ehrenberg) Reimann et J.C.Lewin | ***X*** | ***–*** | ***–*** | ***X*** | ***X*** |  | ***–*** |  |
| 228 | *Cylindrotheca* spp*.* Rebenhorst | ***X*** | ***–*** | ***X*** | ***–*** | ***–*** | ***X (Cnidaria)*** | ***–*** |  |
| 229 | *Cymatopleura elliptica* (Brébisson) W.Smith | ***–*** | ***X*** | ***X*** | ***–*** | ***–*** |  | ***–*** |  |
| 230 | *Cymatopleura solea* (Brébisson) W.Smith | ***–*** | ***X*** | ***X*** | ***–*** | ***X*** |  | ***–*** |  |
| 231 | *Cymatopleura* spp*.* W.Smith | ***–*** | ***X*** | ***–*** | ***X*** | ***–*** |  | ***–*** |  |
| 232 | *Cymatosira belgica* Grunow | ***X*** | ***–*** | ***–*** | ***X*** | ***–*** |  | ***–*** |  |
| 233 | *Cymbella affinis* Kützing | ***–*** | ***X*** | ***X*** | ***–*** | ***X*** |  | ***–*** |  |
| 234 | *Cymbella cistula* (Hemprich) O.Kirchner | ***–*** | ***X*** | ***X*** | ***–*** | ***X*** |  | ***–*** |  |
| 235 | *Cymbella compacta* Østrup | ***–*** | ***X*** | ***–*** | ***–*** | ***X*** |  | ***–*** |  |
| 236 | *Cymbella cymbiformis* C.Agardh | ***–*** | ***X*** | ***–*** | ***–*** | ***X*** |  | ***–*** |  |
| 237 | *Cymbella excisa* Kützing | ***–*** | ***–*** | ***–*** | ***X*** | ***–*** |  | ***–*** |  |
| 238 | *Cymbella excisiformis* Krammer | ***–*** | ***X*** | ***X*** | ***–*** | ***X*** |  | ***–*** |  |
| 239 | *Cymbella hantzschiana* Krammer | ***–*** | ***–*** | ***–*** | ***X*** | ***–*** |  | ***–*** |  |
| 240 | *Cymbella helvetica* Kützing | ***–*** | ***–*** | ***–*** | ***X*** | ***–*** |  | ***–*** |  |
| 241 | *Cymbella hustedtii* Krasske | ***–*** | ***X*** | ***–*** | ***–*** | ***X*** |  | ***–*** |  |
| 242 | *Cymbella lanceolata* (C.Agardh) C.Agardh | ***–*** | ***X*** | ***X*** | ***–*** | ***X*** |  | ***–*** |  |
| 243 | *Cymbella minuta* Hilse | ***–*** | ***X*** | ***X*** | ***–*** | ***X*** |  | ***–*** |  |
| 244 | *Cymbella neolanceolata* W.Silva | ***–*** | ***X*** | ***X*** | ***–*** | ***–*** |  | ***–*** |  |
| 245 | *Cymbella percymbiformis* Krammer | ***–*** | ***X*** | ***–*** | ***–*** | ***X*** |  | ***–*** |  |
| 246 | *Cymbella perparva* Krammer | ***–*** | ***X*** | ***–*** | ***–*** | ***X*** |  | ***–*** |  |
| 247 | *Cymbella* spp*.* C.Agardh | ***–*** | ***X*** | ***X*** | ***X*** | ***–*** |  | ***–*** |  |
| 248 | *Cymbella stigmaphora* Østrup | ***–*** | ***X*** | ***–*** | ***–*** | ***X*** |  | ***–*** |  |
| 249 | *Cymbella subcistula* Krammer | ***–*** | ***X*** | ***X*** | ***–*** | ***–*** |  | ***–*** |  |
| 250 | *Cymbella subhelvetica* Krammer | ***–*** | ***X*** | ***–*** | ***–*** | ***X*** |  | ***–*** |  |
| 251 | *Cymbella subtruncata* Krammer | ***–*** | ***X*** | ***X*** | ***–*** | ***–*** |  | ***–*** |  |
| 252 | *Cymbella tropica* Krammer | ***–*** | ***X*** | ***X*** | ***–*** | ***X*** |  | ***–*** |  |
| 253 | *Cymbella tumida* (Brébisson) Van Heurck | ***–*** | ***X*** | ***X*** | ***–*** | ***X*** |  | ***–*** |  |
| 254 | *Cymbella ventricosa* C.Agardh | ***X*** | ***–*** | ***–*** | ***–*** | ***X*** |  | ***–*** |  |
| 255 | *Cymbella vulgata* Krammer | ***–*** | ***X*** | ***X*** | ***–*** | ***–*** |  | ***–*** |  |
| 256 | *Cymbopleura amphicephala* Krammer | ***–*** | ***X*** | ***X*** | ***–*** | ***X*** |  | ***–*** |  |
| 257 | *Delphineis australis* (P.Petit) Watanabe, Tanaka, Reid, Kumada et Nagumo | ***X*** | ***–*** | ***X*** | ***–*** | ***–*** |  | ***–*** |  |
| 258 | *Delphineis minutissima* (Hustedt) Simonsen | ***X*** | ***–*** | ***–*** | ***X*** | ***X*** |  | ***–*** |  |
| 259 | *Delphineis surirella* (Ehrenberg) G.W.Andrews | ***X*** | ***–*** | ***–*** | ***–*** | ***X*** |  | ***–*** |  |
| 260 | *Denticula kuetzingii* Grunow | ***–*** | ***X*** | ***–*** | ***–*** | ***X*** |  | ***–*** |  |
| 261 | *Denticula* spp*.* Kützing | ***X*** | ***X*** | ***X*** | ***–*** | ***–*** | ***X (Bryozoa, Annelida)*** | ***–*** |  |
| 262 | *Denticula tenuis* Kützing | ***–*** | ***X*** | ***X*** | ***–*** | ***X*** |  | ***–*** |  |
| 263 | *Detonula confervacea* (Cleve) Gran | ***X*** | ***–*** | ***X*** | ***–*** | ***–*** |  | ***–*** |  |
| 264 | *Diadesmis confervacea* Kützing | ***–*** | ***X*** | ***X*** | ***–*** | ***–*** |  | ***–*** |  |
| 265 | *Diadesmis contenta* (Grunow) D.G.Mann | ***–*** | ***X*** | ***X*** | ***–*** | ***X*** |  | ***–*** |  |
| 266 | *Diatoma ehrenbergii* Kützing | ***–*** | ***X*** | ***X*** | ***–*** | ***X*** |  | ***–*** |  |
| 267 | *Diatoma hyemalis* (Roth) Heiberg | ***–*** | ***X*** | ***–*** | ***–*** | ***X*** |  | ***–*** |  |
| 268 | *Diatoma mesodon* (Ehrenberg) Kützing | ***–*** | ***X*** | ***X*** | ***–*** | ***X*** |  | ***–*** |  |
| 269 | *Diatoma moniliformis* Kützing | ***–*** | ***X*** | ***X*** | ***–*** | ***X*** |  | ***–*** |  |
| 270 | *Diatoma polonica* M.Bak, Lange-Bertalot, J.Nosek, Z.Jakubowska et M.Kielbasa | ***–*** | ***X*** | ***X*** | ***–*** | ***–*** |  | ***–*** |  |
| 271 | *Diatoma* spp*.* Bory | ***X*** | ***–*** | ***–*** | ***X*** | ***–*** | ***X (Bryozoa)*** | ***–*** |  |
| 272 | *Diatoma tenuis* C.Agardh | ***–*** | ***X*** | ***X*** | ***–*** | ***–*** |  | ***–*** |  |
| 273 | *Diatoma vulgaris* Bory | ***X*** | ***X*** | ***X*** | ***X*** | ***X*** | ***X (Cnidaria)*** | ***–*** |  |
| 274 | *Dickieia* spp*.* Berkeley | ***X*** | ***–*** | ***X*** | ***–*** | ***–*** |  | ***–*** |  |
| 275 | *Dictyocha* spp*.* Ehrenberg | ***X*** | ***–*** | ***–*** | ***–*** | ***X*** |  | ***–*** |  |
| 276 | *Didymosphenia geminata* (Lyngbye) Mart.Schmidt | ***–*** | ***X*** | ***X*** | ***–*** | ***X*** |  | ***–*** |  |
| 277 | *Dimeregramma maculatum* (Cleve) Frenguelli | ***X*** | ***–*** | ***–*** | ***–*** | ***X*** |  | ***–*** |  |
| 278 | *Dimeregramma minor* (W.Gregory) Ralfs | ***X*** | ***–*** | ***–*** | ***X*** | ***–*** | ***X (Bryozoa)*** | ***–*** |  |
| 279 | *Dimeregramma minor var. nana* (W.Gregory) Van Heurck | ***X*** | ***–*** | ***X*** | ***–*** | ***–*** |  | ***–*** |  |
| 280 | *Dimeregramma* spp*.* Ralfs | ***X*** | ***–*** | ***X*** | ***–*** | ***–*** |  | ***–*** |  |
| 281 | *Diplomenora cocconeiformis* (A.W.F.Schmidt) Blazè | ***X*** | ***–*** | ***X*** | ***–*** | ***–*** |  | ***–*** |  |
| 282 | *Diploneis bombus* Ehrenberg | ***X*** | ***–*** | ***X*** | ***–*** | ***–*** |  | ***–*** |  |
| 283 | *Diploneis cf. stroemi* Hustedt | ***X*** | ***–*** | ***–*** | ***–*** | ***–*** | ***X (Cnidaria)*** | ***–*** |  |
| 284 | *Diploneis coffaeiformis* (A.W.F.Schmidt) Cleve | ***X*** | ***–*** | ***–*** | ***–*** | ***X*** |  | ***–*** |  |
| 285 | *Diploneis constricta* Cleve | ***X*** | ***–*** | ***–*** | ***–*** | ***X*** |  | ***–*** |  |
| 286 | *Diploneis crabro* Ehrenberg | ***X*** | ***–*** | ***–*** | ***–*** | ***X*** | ***X (Porifera)*** | ***–*** |  |
| 287 | *Diploneis didyma* (Ehrenberg) Ehrenberg | ***–*** | ***X*** | ***X*** | ***–*** | ***–*** |  | ***–*** |  |
| 288 | *Diploneis elliptica* (Kützing) Cleve | ***–*** | ***X*** | ***–*** | ***–*** | ***X*** |  | ***–*** |  |
| 289 | *Diploneis fusca* (W.Gregory) Cleve | ***X*** | ***–*** | ***–*** | ***X*** | ***–*** |  | ***–*** |  |
| 290 | *Diploneis gravelleana* Hagelstein | ***X*** | ***–*** | ***–*** | ***–*** | ***X*** |  | ***–*** |  |
| 291 | *Diploneis gruendleri* (A.W.F.Schmidt) Cleve | ***X*** | ***–*** | ***–*** | ***–*** | ***X*** |  | ***–*** |  |
| 292 | *Diploneis incurvata* (W.Gregory) Cleve | ***X*** | ***–*** | ***–*** | ***–*** | ***X*** |  | ***–*** |  |
| 293 | *Diploneis ingens* (Mann) VanLandigham | ***X*** | ***–*** | ***X*** | ***–*** | ***–*** |  | ***–*** |  |
| 294 | *Diploneis litoralis* (Donkin) Cleve | ***X*** | ***–*** | ***X*** | ***–*** | ***–*** | ***X (Bryozoa)*** | ***–*** |  |
| 295 | *Diploneis obliqua* (Brun) Hustedt | ***X*** | ***–*** | ***–*** | ***–*** | ***X*** |  | ***–*** |  |
| 296 | *Diploneis oblongella* (Nägeli) A.Cleve | ***–*** | ***X*** | ***–*** | ***–*** | ***X*** |  | ***–*** |  |
| 297 | *Diploneis oculata* (Brébisson) Cleve | ***X*** | ***X*** | ***–*** | ***–*** | ***X*** | ***X (Cnidaria)*** | ***–*** |  |
| 298 | *Diploneis papula* (A.W.F.Schmidt) Cleve | ***X*** | ***–*** | ***–*** | ***X*** | ***–*** |  | ***–*** |  |
| 299 | *Diploneis parma* Cleve | ***–*** | ***X*** | ***–*** | ***–*** | ***X*** |  | ***–*** |  |
| 300 | *Diploneis separanda* Lange-Bertalot | ***–*** | ***X*** | ***–*** | ***–*** | ***X*** |  | ***–*** |  |
| 301 | *Diploneis smithii* (Brébisson) Cleve | ***X*** | ***–*** | ***–*** | ***X*** | ***X*** |  | ***–*** |  |
| 302 | *Diploneis splendida* (W.Gregory) Cleve | ***X*** | ***–*** | ***–*** | ***X*** | ***–*** |  | ***–*** |  |
| 303 | *Diploneis* spp*.* Ehrenberg | ***X*** | ***X*** | ***X*** | ***–*** | ***X*** |  | ***–*** |  |
| 304 | *Diploneis vacillans* (A.W.F.Schmidt) Cleve | ***X*** | ***–*** | ***–*** | ***X*** | ***X*** |  | ***–*** |  |
| 305 | *Diploneis vacillans var. delicatula* Cleve | ***X*** | ***–*** | ***–*** | ***–*** | ***X*** |  | ***–*** |  |
| 306 | *Discostella pseudostelligera* (Hustedt) Houk et Klee | ***–*** | ***X*** | ***X*** | ***–*** | ***–*** |  | ***–*** |  |
| 307 | *Discostella stelligera* (Cleve et Grunow) Houk et Klee | ***–*** | ***X*** | ***X*** | ***–*** | ***–*** |  | ***–*** |  |
| 308 | *Distrionella incognita* (E.Reichardt) D.M.Williams | ***–*** | ***X*** | ***X*** | ***–*** | ***–*** |  | ***–*** |  |
| 309 | *Ditylum* spp*.* Bailey | ***X*** | ***–*** | ***–*** | ***–*** | ***X*** |  | ***–*** |  |
| 310 | *Druehlago lobbanii* Majewska, Mayombo, Smit et Goosen | ***X*** | ***–*** | ***–*** | ***–*** | ***–*** | ***X (Porifera)*** | ***–*** |  |
| 311 | *Encyonema brehmiforme* Krammer | ***–*** | ***X*** | ***–*** | ***–*** | ***X*** |  | ***–*** |  |
| 312 | *Encyonema caespitosum* Kützing | ***–*** | ***X*** | ***–*** | ***–*** | ***X*** |  | ***–*** |  |
| 313 | *Encyonema lancettulum* Krammer | ***–*** | ***X*** | ***–*** | ***–*** | ***X*** |  | ***–*** |  |
| 314 | *Encyonema minutum* (Hilse) D.G.Mann | ***X*** | ***X*** | ***X*** | ***–*** | ***X*** |  | ***–*** |  |
| 315 | *Encyonema prostratum* (Barkeley) Kützing | ***–*** | ***X*** | ***X*** | ***–*** | ***X*** |  | ***–*** |  |
| 316 | *Encyonema pseudocaespitosum* Levkov et Krstic | ***–*** | ***–*** | ***–*** | ***X*** | ***–*** |  | ***–*** |  |
| 317 | *Encyonema silesiacum* (Bleisch) D.G.Mann | ***–*** | ***X*** | ***X*** | ***X*** | ***X*** |  | ***–*** |  |
| 318 | *Encyonema* spp*.* Kützing | ***–*** | ***X*** | ***X*** | ***–*** | ***X*** |  | ***–*** |  |
| 319 | *Encyonema ventricosum* (C.Agardh) Grunow | ***–*** | ***X*** | ***X*** | ***–*** | ***X*** |  | ***–*** |  |
| 320 | *Encyonopsis cesatii* (Rabenhorst) Krammer | ***–*** | ***X*** | ***–*** | ***–*** | ***X*** |  | ***–*** |  |
| 321 | *Encyonopsis microcephala* (Grunow) Krammer | ***–*** | ***X*** | ***–*** | ***X*** | ***–*** |  | ***–*** |  |
| 322 | *Encyonopsis minuta* Krammer et E.Reichardt | ***–*** | ***X*** | ***X*** | ***–*** | ***X*** |  | ***–*** |  |
| 323 | *Encyonopsis subminuta* Krammer et E.Reichardt | ***–*** | ***X*** | ***X*** | ***–*** | ***X*** |  | ***–*** |  |
| 324 | *Entomoneis alata* Ehrenberg | ***X*** | ***–*** | ***–*** | ***X*** | ***–*** |  | ***–*** |  |
| 325 | *Entomoneis cf. paludosa* (W.Smith) Reimer | ***X*** | ***–*** | ***–*** | ***X*** | ***–*** |  | ***–*** |  |
| 326 | *Entomoneis paludosa* (W.Smith) Reimer | ***X*** | ***–*** | ***–*** | ***X*** | ***X*** | ***X (Porifera)*** | ***–*** |  |
| 327 | *Entomoneis paludosa var. hyperborea* (W.Smith) Reimer | ***X*** | ***–*** | ***–*** | ***X*** | ***–*** |  | ***–*** |  |
| 328 | *Entomoneis* spp*.* Ehrenberg | ***X*** | ***–*** | ***X*** | ***–*** | ***–*** |  | ***–*** |  |
| 329 | *Epipellis heptunei*^[1][2]^ Denys et Van Bonn | ***X*** | ***–*** | ***X*^[1]^** | ***–*** | ***–*** |  | **^[1][2]^Artiodactyla (*Tursiops truncatus*)** | **Denys & Van Bonn (2001)** |
| 330 | *Epipellis oiketis*^[1][2]^ R.W.Holmes | ***X*** | ***–*** | ***X*^[1]^** | ***–*** | ***X*** |  | **^[1][2]^Artiodactyla (*Orcinus orca; Phocoenoides dalli*)** | **Holmes (1985)** |
| 331 | *Epiphalaina aleutica*^[1]^ (Nemoto) R.W.Holmes, Nagasawa et Takano | ***X*** | ***–*** | ***X*^[1]^** | ***–*** | ***–*** |  | ***–*** |  |
| 332 | *Epiphalaina aleutica var. lineata*^[1]^ Denys | ***X*** | ***–*** | ***X*^[1]^** | ***–*** | ***–*** |  | ***–*** |  |
| 333 | *Epithemia adnata* (Kützing) Brébisson | ***–*** | ***X*** | ***X*** | ***X*** | ***X*** |  | ***–*** |  |
| 334 | *Epithemia goeppertiana* Hilse | ***–*** | ***X*** | ***X*** | ***–*** | ***–*** |  | ***–*** |  |
| 335 | *Epithemia sorex* Kützing | ***–*** | ***X*** | ***X*** | ***–*** | ***X*** |  | ***–*** |  |
| 336 | *Epithemia* spp*.* Kützing | ***–*** | ***X*** | ***–*** | ***X*** | ***X*** |  | ***–*** |  |
| 337 | *Epithemia turgida* (Ehrenberg) Kützing | ***–*** | ***X*** | ***–*** | ***X*** | ***–*** |  | ***–*** |  |
| 338 | *Eucocconeis flexella* (Kützing) F.Meister | ***–*** | ***X*** | ***–*** | ***–*** | ***X*** |  | ***–*** |  |
| 339 | *Eucocconeis laevis* (Østrup) Lange-Bertalot | ***–*** | ***X*** | ***–*** | ***–*** | ***X*** |  | ***–*** |  |
| 340 | *Eunotia baculus* Hustedt | ***–*** | ***X*** | ***X*** | ***–*** | ***–*** |  | ***–*** |  |
| 341 | *Eunotia bidens* Ehrenberg | ***–*** | ***X*** | ***–*** | ***X*** | ***–*** |  | ***–*** |  |
| 342 | *Eunotia bidentula* W.Smith | ***–*** | ***X*** | ***X*** | ***–*** | ***–*** |  | ***–*** |  |
| 343 | *Eunotia bilunaris* (Ehrenberg) Schaarschmidt | ***–*** | ***X*** | ***X*** | ***–*** | ***–*** |  | ***–*** |  |
| 344 | *Eunotia circumborealis* Lange-Bertalot et Nӧrpel | ***–*** | ***X*** | ***X*** | ***–*** | ***–*** |  | ***–*** |  |
| 345 | *Eunotia conversa* Hustedt | ***–*** | ***X*** | ***X*** | ***–*** | ***–*** |  | ***–*** |  |
| 346 | *Eunotia convexa* Hustedt | ***–*** | ***X*** | ***X*** | ***–*** | ***–*** |  | ***–*** |  |
| 347 | *Eunotia exigua* (Brébisson) Rabenhorst | ***–*** | ***X*** | ***X*** | ***–*** | ***–*** |  | ***–*** |  |
| 348 | *Eunotia glacialis* F.Meister | ***–*** | ***X*** | ***X*** | ***–*** | ***–*** |  | ***–*** |  |
| 349 | *Eunotia glacialispinosa* Lange-Bertalot et Cantonati | ***–*** | ***X*** | ***–*** | ***X*** | ***–*** |  | ***–*** |  |
| 350 | *Eunotia minor* (Kützing) Grunow | ***–*** | ***X*** | ***X*** | ***–*** | ***–*** |  | ***–*** |  |
| 351 | *Eunotia pseudosudetica* Metzeltin, Lange-Bertalot et García-Rodriguez | ***–*** | ***X*** | ***–*** | ***X*** | ***–*** |  | ***–*** |  |
| 352 | *Eunotia reflexa* Hustedt | ***–*** | ***X*** | ***X*** | ***–*** | ***–*** |  | ***–*** |  |
| 353 | *Eunotia sedina* Lange-Bertalot, Bąket Witkowski | ***–*** | ***X*** | ***–*** | ***X*** | ***–*** |  | ***–*** |  |
| 354 | *Eunotia seminulum* Nӧrpel-Schemp et Lange-Bertalot | ***–*** | ***X*** | ***–*** | ***X*** | ***–*** |  | ***–*** |  |
| 355 | *Eunotia soleirolii* (Kützing) Rabenhorst | ***–*** | ***X*** | ***X*** | ***–*** | ***–*** |  | ***–*** |  |
| 356 | *Eunotia* spp*.* Ehrenberg | ***–*** | ***X*** | ***X*** | ***–*** | ***X*** |  | ***–*** |  |
| 357 | *Eunotia sulcatoides* Metzeltin et Lange-Bertalot | ***–*** | ***X*** | ***X*** | ***–*** | ***–*** |  | ***–*** |  |
| 358 | *Eunotogramma dubium* Hustedt | ***X*** | ***–*** | ***–*** | ***X*** | ***–*** |  | ***–*** |  |
| 359 | *Eunotogramma litorale* Amspoker | ***X*** | ***–*** | ***–*** | ***–*** | ***X*** |  | ***–*** |  |
| 360 | *Eunotogramma wueissei* Ehrenberg | ***X*** | ***–*** | ***–*** | ***–*** | ***–*** | ***X (Bryozoa)*** | ***–*** |  |
| 361 | *Falcula hyalina*^[1][2]^ Takano | ***X*** | ***–*** | ***–*** | ***–*** | ***X*^[1]^** |  | **^[1][2]^Calanoida (*Acartia steueri*)** | **Takano (1983)** |
| 362 | *Fallacia cassubiae* Witkowski | ***X*** | ***–*** | ***X*** | ***–*** | ***–*** |  | ***–*** |  |
| 363 | *Fallacia florinae* (Møller) Witkowski | ***X*** | ***–*** | ***X*** | ***–*** | ***–*** |  | ***–*** |  |
| 364 | *Fallacia lenzii* (Hustedt) Lange-Bertalot | ***–*** | ***X*** | ***X*** | ***–*** | ***X*** |  | ***–*** |  |
| 365 | *Fallacia litoricola* (Hustedt) D.G.Mann | ***X*** | ***–*** | ***–*** | ***–*** | ***X*** |  | ***–*** |  |
| 366 | *Fallacia nummularia* (Greville) Mann | ***X*** | ***–*** | ***–*** | ***–*** | ***X*** |  | ***–*** |  |
| 367 | *Fallacia oculiformis* (Hustedt) D.G.Mann | ***X*** | ***–*** | ***X*** | ***–*** | ***–*** |  | ***–*** |  |
| 368 | *Fallacia pygmaea* (Kützing) Stickle et D.G.Mann | ***X*** | ***X*** | ***X*** | ***–*** | ***X*** |  | ***–*** |  |
| 369 | *Fallacia scaldensis* Sabbe et Muylaert | ***X*** | ***–*** | ***–*** | ***X*** | ***–*** |  | ***–*** |  |
| 370 | *Fallacia* spp*.* Stickle et Mann | ***X*** | ***–*** | ***X*** | ***–*** | ***–*** | ***X (Bryozoa)*** | ***–*** |  |
| 371 | *Fallacia subforcipata* (Hustedt) D.G.Mann | ***X*** | ***–*** | ***X*** | ***X*** | ***–*** |  | ***–*** |  |
| 372 | *Fallacia subhamulata* (Grunow) D.G.Mann | ***–*** | ***X*** | ***X*** | ***–*** | ***X*** |  | ***–*** |  |
| 373 | *Fallacia sublucidula* (Hustedt) D.G.Mann | ***–*** | ***X*** | ***X*** | ***–*** | ***–*** |  | ***–*** |  |
| 374 | *Fallacia tenera* (Hustedt) D.G.Mann | ***X*** | ***–*** | ***–*** | ***X*** | ***–*** |  | ***–*** |  |
| 375 | *Fistulifera pelliculosa* (Brébisson) Lange-Bertalot | ***–*** | ***X*** | ***X*** | ***–*** | ***–*** |  | ***–*** |  |
| 376 | *Fistulifera saprophila* (Lange-Bertalot et Bonik) Lange-Bertalot | ***–*** | ***X*** | ***X*** | ***–*** | ***–*** |  | ***–*** |  |
| 377 | *Fragilaria acus* (Ehrenberg) Cleve | ***–*** | ***X*** | ***–*** | ***–*** | ***X*** |  | ***–*** |  |
| 378 | *Fragilaria amphicephaloides* Lange-Bertalot | ***–*** | ***X*** | ***–*** | ***–*** | ***X*** |  | ***–*** |  |
| 379 | *Fragilaria anceps* Ehrenberg | ***X*** | ***–*** | ***–*** | ***–*** | ***–*** | ***X (Cnidaria)*** | ***–*** |  |
| 380 | *Fragilaria austriaca* (Grunow) Lange-Bertalot | ***–*** | ***X*** | ***X*** | ***–*** | ***–*** |  | ***–*** |  |
| 381 | *Fragilaria candidagilae* Almeida, C.Delgado, Novais et S.Blanco | ***–*** | ***X*** | ***–*** | ***–*** | ***X*** |  | ***–*** |  |
| 382 | *Fragilaria capucina* Desmazières | ***X*** | ***X*** | ***X*** | ***–*** | ***X*** |  | ***–*** |  |
| 383 | *Fragilaria constricta* Ehrenberg | ***–*** | ***X*** | ***–*** | ***–*** | ***X*** |  | ***–*** |  |
| 384 | *Fragilaria construens var. binodis* (Ehrenberg) Grunow | ***–*** | ***X*** | ***–*** | ***–*** | ***X*** |  | ***–*** |  |
| 385 | *Fragilaria deformis* (W.Smith) Van de Vijver et Ector | ***–*** | ***X*** | ***X*** | ***–*** | ***–*** |  | ***–*** |  |
| 386 | *Fragilaria delicatissima* (W.Smith) Lange-Bertalot | ***–*** | ***X*** | ***–*** | ***–*** | ***X*** |  | ***–*** |  |
| 387 | *Fragilaria geocollegarum* Witkowski et Lange-Bertalot | ***X*** | ***–*** | ***–*** | ***X*** | ***–*** |  | ***–*** |  |
| 388 | *Fragilaria gracilis* Østrup | ***–*** | ***X*** | ***X*** | ***–*** | ***X*** |  | ***–*** |  |
| 389 | *Fragilaria hyalina* (Kützing) Grunow | ***X*** | ***–*** | ***–*** | ***X*** | ***–*** |  | ***–*** |  |
| 390 | *Fragilaria investiens* (W.Smith) A.Cleve | ***X*** | ***–*** | ***X*** | ***–*** | ***–*** |  | ***–*** |  |
| 391 | *Fragilaria mesolepta* Rabenhorst | ***–*** | ***X*** | ***–*** | ***–*** | ***X*** |  | ***–*** |  |
| 392 | *Fragilaria neointermedia* Tuji et D.M.Williams | ***–*** | ***X*** | ***X*** | ***–*** | ***–*** |  | ***–*** |  |
| 393 | *Fragilaria oceanica* Cleve | ***X*** | ***–*** | ***–*** | ***X*** | ***–*** |  | ***–*** |  |
| 394 | *Fragilaria pararumpens* Lange-Bertalot | ***–*** | ***X*** | ***–*** | ***–*** | ***X*** |  | ***–*** |  |
| 395 | *Fragilaria perminuta* (Grunow) Lange-Bertalot | ***–*** | ***X*** | ***–*** | ***–*** | ***X*** |  | ***–*** |  |
| 396 | *Fragilaria radians* (Kützing) D.M.Williams et Round | ***–*** | ***X*** | ***X*** | ***–*** | ***X*** |  | ***–*** |  |
| 397 | *Fragilaria recapitellata* Lange-Bertalot et Metzeltin | ***–*** | ***X*** | ***X*** | ***–*** | ***–*** |  | ***–*** |  |
| 398 | *Fragilaria rumpens* (Kützing) Carlson | ***–*** | ***X*** | ***X*** | ***X*** | ***X*** |  | ***–*** |  |
| 399 | *Fragilaria* spp*.* Lyngbye | ***X*** | ***X*** | ***X*** | ***X*** | ***X*** | ***X (Bryozoa)*** | ***–*** |  |
| 400 | *Fragilaria subrecapitellata* Van de Vijver, Hurlimann, D.M.Williams, Wetzel et Ector | ***–*** | ***X*** | ***X*** | ***–*** | ***–*** |  | ***–*** |  |
| 401 | *Fragilaria tenera* (W.Smith) Lange-Bertalot | ***–*** | ***X*** | ***–*** | ***–*** | ***X*** |  | ***–*** |  |
| 402 | *Fragilaria vaucheriae* (Kützing) Petersen | ***–*** | ***X*** | ***X*** | ***–*** | ***X*** |  | ***–*** |  |
| 403 | *Fragilaria virescens* Ralfs | ***–*** | ***X*** | ***–*** | ***X*** | ***–*** |  | ***–*** |  |
| 404 | *Fragilariforma hamiltonii* Metzeltin et Lange-Bertalot | ***–*** | ***X*** | ***X*** | ***–*** | ***–*** |  | ***–*** |  |
| 405 | *Fragilariopsis curta* (Van Heurck) Hustedt | ***X*** | ***–*** | ***–*** | ***–*** | ***–*** | ***X (Porifera)*** | ***–*** |  |
| 406 | *Fragilariopsis kerguelensis* (O'Meara) Hustedt | ***X*** | ***–*** | ***X*** | ***–*** | ***–*** |  | ***–*** |  |
| 407 | *Fragilariopsis rhombica* (O'Meara) Hustedt | ***X*** | ***–*** | ***X*** | ***–*** | ***–*** |  | ***–*** |  |
| 408 | *Frustulia crassinerva* (Brébisson) Lange-Bertalot et Krammer | ***–*** | ***X*** | ***X*** | ***–*** | ***–*** |  | ***–*** |  |
| 409 | *Frustulia rhomboides* (Ehrenberg) De Toni | ***–*** | ***X*** | ***X*** | ***–*** | ***–*** |  | ***–*** |  |
| 410 | *Frustulia vulgaris* (Thwaites) De Toni | ***–*** | ***X*** | ***X*** | ***–*** | ***–*** |  | ***–*** |  |
| 411 | *Geissleria acceptata* (Hustedt) Lange-Bertalot et Metzeltin | ***–*** | ***X*** | ***X*** | ***–*** | ***X*** |  | ***–*** |  |
| 412 | *Geissleria decussis* (Østrup) Lange-Bertalot et Metzeltin | ***–*** | ***X*** | ***X*** | ***–*** | ***X*** |  | ***–*** |  |
| 413 | *Geissleria* spp*.* Lange-Bertalot et Metzeltin | ***–*** | ***X*** | ***X*** | ***–*** | ***–*** |  | ***–*** |  |
| 414 | *Glyphodesmis distans* (W.Gregory) Grunow | ***X*** | ***–*** | ***–*** | ***–*** | ***–*** | ***X (Bryozoa)*** | ***–*** |  |
| 415 | *Gommatophora oceanica* Ehrenberg | ***X*** | ***–*** | ***–*** | ***–*** | ***X*** |  | ***–*** |  |
| 416 | *Gomphoneis* spp*.* Cleve | ***X*** | ***–*** | ***–*** | ***X*** | ***–*** |  | ***–*** |  |
| 417 | *Gomphonella olivacea* (Hornemann) Rabenhorst | ***–*** | ***X*** | ***X*** | ***–*** | ***X*** |  | ***–*** |  |
| 418 | *Gomphonema aestuarii* Cleve | ***X*** | ***–*** | ***X*** | ***–*** | ***–*** |  | ***–*** |  |
| 419 | *Gomphonema auritum* A.Braun | ***–*** | ***X*** | ***X*** | ***–*** | ***–*** |  | ***–*** |  |
| 420 | *Gomphonema boreale* Nemoto | ***X*** | ***–*** | ***X*** | ***–*** | ***–*** |  | ***–*** |  |
| 421 | *Gomphonema calcifugum* Lange-Bertalot et E.Reichardt | ***–*** | ***X*** | ***X*** | ***–*** | ***–*** |  | ***–*** |  |
| 422 | *Gomphonema capitatum* Ehrenberg | ***–*** | ***X*** | ***X*** | ***–*** | ***X*** |  | ***–*** |  |
| 423 | *Gomphonema coronatum* Ehrenberg | ***–*** | ***X*** | ***–*** | ***–*** | ***X*** |  | ***–*** |  |
| 424 | *Gomphonema cuneolus* E.Reichardt | ***–*** | ***X*** | ***–*** | ***–*** | ***X*** |  | ***–*** |  |
| 425 | *Gomphonema cymbelliclinum* E.Reichardt et Lange-Bertalot | ***–*** | ***X*** | ***–*** | ***–*** | ***X*** |  | ***–*** |  |
| 426 | *Gomphonema elegantissimum* E.Reichardt et Lange-Bertalot | ***–*** | ***X*** | ***X*** | ***–*** | ***X*** |  | ***–*** |  |
| 427 | *Gomphonema exiguum* Kützing | ***X*** | ***–*** | ***–*** | ***X*** | ***–*** |  | ***–*** |  |
| 428 | *Gomphonema exilissimum* (Grunow) Lange-Bertalot et E.Reichardt | ***–*** | ***X*** | ***X*** | ***–*** | ***–*** |  | ***–*** |  |
| 429 | *Gomphonema gracile* Ehrenberg | ***–*** | ***X*** | ***X*** | ***X*** | ***–*** |  | ***–*** |  |
| 430 | *Gomphonema harti* Nemoto | ***X*** | ***–*** | ***X*** | ***–*** | ***–*** |  | ***–*** |  |
| 431 | *Gomphonema insigne* W.Gregory | ***–*** | ***X*** | ***X*** | ***–*** | ***–*** |  | ***–*** |  |
| 432 | *Gomphonema intricatum* Kützing | ***–*** | ***X*** | ***–*** | ***–*** | ***X*** |  | ***–*** |  |
| 433 | *Gomphonema intricatum v. pumila* Grunow | ***–*** | ***X*** | ***–*** | ***–*** | ***X*** |  | ***–*** |  |
| 434 | *Gomphonema italicum* Kützing | ***–*** | ***X*** | ***X*** | ***–*** | ***–*** |  | ***–*** |  |
| 435 | *Gomphonema kamtschaticum* Grunow | ***X*** | ***–*** | ***X*** | ***–*** | ***–*** |  | ***–*** |  |
| 436 | *Gomphonema kamtschaticum var. californicum* Grunow | ***X*** | ***–*** | ***X*** | ***–*** | ***–*** |  | ***–*** |  |
| 437 | *Gomphonema lagenula* Kützing | ***–*** | ***X*** | ***X*** | ***–*** | ***–*** |  | ***–*** |  |
| 438 | *Gomphonema lateripunctatum* E.Reichardt et Lange-Bertalot | ***–*** | ***X*** | ***–*** | ***–*** | ***X*** |  | ***–*** |  |
| 439 | *Gomphonema longiceps v. subclavata* Ehrenberg | ***–*** | ***X*** | ***–*** | ***–*** | ***X*** |  | ***–*** |  |
| 440 | *Gomphonema mexicanum* Grunow | ***–*** | ***X*** | ***–*** | ***X*** | ***–*** |  | ***–*** |  |
| 441 | *Gomphonema micropus* Kützing | ***–*** | ***X*** | ***X*** | ***–*** | ***X*** |  | ***–*** |  |
| 442 | *Gomphonema minutum* (C.Agardh) C.Agardh | ***–*** | ***X*** | ***X*** | ***–*** | ***X*** |  | ***–*** |  |
| 443 | *Gomphonema olivaceoides* Hustedt | ***–*** | ***X*** | ***X*** | ***–*** | ***X*** |  | ***–*** |  |
| 444 | *Gomphonema paludosum* E.Reichardt | ***–*** | ***X*** | ***–*** | ***X*** | ***–*** |  | ***–*** |  |
| 445 | *Gomphonema parvulum* (Kützing) Kützing | ***–*** | ***X*** | ***X*** | ***X*** | ***X*** |  | ***–*** |  |
| 446 | *Gomphonema parvulum f. anormale* (Kützing) Kützing | ***–*** | ***X*** | ***X*** | ***–*** | ***–*** |  | ***–*** |  |
| 447 | *Gomphonema pumilum* (Grunow) E.Reichardt et Lange-Bertalot | ***–*** | ***X*** | ***X*** | ***–*** | ***–*** |  | ***–*** |  |
| 448 | *Gomphonema pumilum var. rigidum* E.Reichardt et Lange-Bertalot | ***–*** | ***X*** | ***X*** | ***–*** | ***X*** |  | ***–*** |  |
| 449 | *Gomphonema rhombicum* A.W.F.Schmidt | ***–*** | ***X*** | ***X*** | ***–*** | ***–*** |  | ***–*** |  |
| 450 | *Gomphonema sphaerophorum* Ehrenberg | ***–*** | ***X*** | ***–*** | ***–*** | ***X*** |  | ***–*** |  |
| 451 | *Gomphonema* spp*.* Ehrenberg | ***X*** | ***X*** | ***X*** | ***X*** | ***X*** |  | ***–*** |  |
| 452 | *Gomphonema subclavatum* Grunow | ***–*** | ***X*** | ***–*** | ***–*** | ***X*** |  | ***–*** |  |
| 453 | *Gomphonema tergestinum* (Grunow) A.W.F.Schmidt | ***–*** | ***X*** | ***X*** | ***–*** | ***X*** |  | ***–*** |  |
| 454 | *Gomphonema truncatum* Ehrenberg | ***–*** | ***X*** | ***X*** | ***–*** | ***–*** |  | ***–*** |  |
| 455 | *Gomphonema truncatum var. capitatum* (Ehrenberg) Patrick | ***–*** | ***X*** | ***–*** | ***–*** | ***X*** |  | ***–*** |  |
| 456 | *Gomphonema varians* Reichardt et Levkov | ***–*** | ***X*** | ***–*** | ***–*** | ***X*** |  | ***–*** |  |
| 457 | *Gomphonema ventricosum* W.Gregory | ***–*** | ***X*** | ***X*** | ***–*** | ***–*** |  | ***–*** |  |
| 458 | *Gomphonemopsis* spp*.* Medlin | ***X*** | ***–*** | ***–*** | ***X*** | ***–*** |  | ***–*** |  |
| 459 | *Gomphoseptatum aestuarii* (Cleve) Medlin | ***X*** | ***–*** | ***–*** | ***–*** | ***X*** | ***X (Bryozoa)*** | ***–*** |  |
| 460 | *Gomphosphenia holmquistii* (Foged) Lange-Bertalot | ***–*** | ***X*** | ***–*** | ***–*** | ***X*** |  | ***–*** |  |
| 461 | *Gomphosphenia tackei* (Hustedt) Lange-Bertalot | ***–*** | ***X*** | ***–*** | ***X*** | ***–*** |  | ***–*** |  |
| 462 | *Grammatophora angulosa* Ehrenberg | ***X*** | ***–*** | ***X*** | ***–*** | ***X*** | ***X (Cnidaria)*** | ***–*** |  |
| 463 | *Grammatophora macilenta* W.Smith | ***X*** | ***–*** | ***–*** | ***–*** | ***X*** |  | ***–*** |  |
| 464 | *Grammatophora malleus* (Brightwell) Van Heurck | ***X*** | ***–*** | ***–*** | ***–*** | ***X*** |  | ***–*** |  |
| 465 | *Grammatophora marina* (Lyngbye) Kützing | ***X*** | ***–*** | ***–*** | ***X*** | ***X*** | ***X (Cnidaria, Bryozoa)*** | ***–*** |  |
| 466 | *Grammatophora* spp*.* Ehrenberg | ***X*** | ***–*** | ***–*** | ***–*** | ***X*** | ***X (Bryozoa)*** | ***–*** |  |
| 467 | *Grammatophora undulata* Ehrenberg | ***X*** | ***–*** | ***X*** | ***–*** | ***–*** |  | ***–*** |  |
| 468 | *Gyrosigma acuminatum* (Kützing) Rabenhorst | ***–*** | ***X*** | ***X*** | ***–*** | ***–*** |  | ***–*** |  |
| 469 | *Gyrosigma acuminatum var. gallica* (Grunow) Cleve | ***X*** | ***–*** | ***–*** | ***X*** | ***–*** |  | ***–*** |  |
| 470 | *Gyrosigma attenuatum* (Kützing) Rabenhorst | ***–*** | ***X*** | ***X*** | ***–*** | ***–*** |  | ***–*** |  |
| 471 | *Gyrosigma balticum* (Ehrenberg) Rabenhorst | ***X*** | ***X*** | ***X*** | ***–*** | ***X*** |  | ***–*** |  |
| 472 | *Gyrosigma cf. limosum* Sterrenburg et Underwood | ***X*** | ***–*** | ***–*** | ***X*** | ***–*** |  | ***–*** |  |
| 473 | *Gyrosigma distortum* (W.Smith) Griffith et Henfrey | ***X*** | ***–*** | ***–*** | ***X*** | ***–*** |  | ***–*** |  |
| 474 | *Gyrosigma eximium* (Thwaites) Boyer | ***X*** | ***–*** | ***–*** | ***–*** | ***X*** |  | ***–*** |  |
| 475 | *Gyrosigma hummii* Hustedt | ***X*** | ***–*** | ***X*** | ***–*** | ***–*** |  | ***–*** |  |
| 476 | *Gyrosigma kuetzingii* (Grunow) Cleve | ***–*** | ***X*** | ***X*** | ***–*** | ***X*** |  | ***–*** |  |
| 477 | *Gyrosigma littorale* (W.Smith) Griffith et Henfrey | ***X*** | ***–*** | ***–*** | ***X*** | ***–*** |  | ***–*** |  |
| 478 | *Gyrosigma peisonis* (Grunow) Hustedt | ***X*** | ***–*** | ***–*** | ***–*** | ***X*** |  | ***–*** |  |
| 479 | *Gyrosigma sciotoense* (W.S.Sullivan) Cleve | ***–*** | ***X*** | ***X*** | ***–*** | ***X*** |  | ***–*** |  |
| 480 | *Gyrosigma* spp*.* Hassall | ***X*** | ***X*** | ***X*** | ***X*** | ***X*** | ***X (Porifera)*** | ***–*** |  |
| 481 | *Gyrosigma variistriatum* Hagelstein | ***X*** | ***–*** | ***–*** | ***–*** | ***X*** |  | ***–*** |  |
| 482 | *Halamphora acutiuscula* (Kützing) Levkov | ***X*** | ***–*** | ***X*** | ***–*** | ***X*** |  | ***–*** |  |
| 483 | *Halamphora aff. granulata* W.Gregory | ***X*** | ***–*** | ***–*** | ***–*** | ***X*** |  | ***–*** |  |
| 484 | *Halamphora capitata* (R.Hagelstein) Álvarez-Blanco et S.Blanco | ***–*** | ***X*** | ***X*** | ***–*** | ***–*** |  | ***–*** |  |
| 485 | *Halamphora cf. abuensis* (Foged) Levkov | ***X*** | ***–*** | ***–*** | ***X*** | ***–*** |  | ***–*** |  |
| 486 | *Halamphora coffeaeformis* (C.Agardh) Levkov | ***X*** | ***–*** | ***–*** | ***X*** | ***X*** |  | ***–*** |  |
| 487 | *Halamphora holsatica* (Hustedt) Levkov | ***X*** | ***–*** | ***–*** | ***–*** | ***X*** |  | ***–*** |  |
| 488 | *Halamphora luciae* (Cholnoky) Levkov | ***X*** | ***–*** | ***X*** | ***–*** | ***–*** |  | ***–*** |  |
| 489 | *Halamphora montana* (Krasske) Levkov | ***–*** | ***X*** | ***X*** | ***–*** | ***–*** |  | ***–*** |  |
| 490 | *Halamphora* spp*.* (Cleve) Levkov | ***X*** | ***–*** | ***X*** | ***X*** | ***–*** |  | ***–*** |  |
| 491 | *Halamphora subangularis* (Hustedt) Levkov | ***X*** | ***–*** | ***X*** | ***–*** | ***–*** |  | ***–*** |  |
| 492 | *Halamphora tenerrima* (Aleem et Hustedt) Levkov | ***X*** | ***–*** | ***X*** | ***–*** | ***–*** |  | ***–*** |  |
| 493 | *Halamphora veneta* (Kützing) Levkov | ***–*** | ***X*** | ***X*** | ***–*** | ***–*** |  | ***–*** |  |
| 494 | *Hannaea arcus* (Ehrenberg) Patrick | ***–*** | ***X*** | ***X*** | ***–*** | ***–*** |  | ***–*** |  |
| 495 | *Hantzschia amphioxys* (Ehrenberg) Grunow | ***–*** | ***X*** | ***X*** | ***–*** | ***–*** |  | ***–*** |  |
| 496 | *Hantzschia* sp. Grunow | ***X*** | ***–*** | ***–*** | ***–*** | ***X*** |  | ***–*** |  |
| 497 | *Haslea amicorum* Herwig, Tiffany, Hargraves et Sterrenburg | ***X*** | ***–*** | ***X*** | ***–*** | ***–*** |  | ***–*** |  |
| 498 | *Haslea* spp*.* Simonsen | ***X*** | ***–*** | ***X*** | ***–*** | ***X*** |  | ***–*** |  |
| 499 | *Haslea wawrikae* (Hustedt) Simonsen | ***X*** | ***–*** | ***X*** | ***–*** | ***–*** |  | ***–*** |  |
| 500 | *Hemidiscus* sp. Wallich | ***X*** | ***–*** | ***–*** | ***–*** | ***X*** |  | ***–*** |  |
| 501 | *Hippodonta caotica* Witkowski, Lange-Bertalot et Metzeltin | ***X*** | ***–*** | ***–*** | ***X*** | ***–*** |  | ***–*** |  |
| 502 | *Hippodonta capitata* (Ehrenberg) Lange-Bertalot, Metzeltin et Witkowski | ***–*** | ***X*** | ***X*** | ***–*** | ***–*** |  | ***–*** |  |
| 503 | *Hippodonta cf. pseudacceptata* (H.Kobayasi) Lange-Bertalot, Metzeltin et Witkowski | ***X*** | ***–*** | ***–*** | ***X*** | ***–*** |  | ***–*** |  |
| 504 | *Hippodonta pseudacceptata* (H.Kobayasi) Lange-Bertalot, Metzeltin et Witkowski | ***–*** | ***X*** | ***X*** | ***–*** | ***–*** |  | ***–*** |  |
| 505 | *Hippodonta* spp*.* Lange-Bertalot, Metzeltin et Witkowski | ***X*** | ***–*** | ***X*** | ***–*** | ***–*** | ***X (Echinodermata)*** | ***–*** |  |
| 506 | *Homoeocladia angularis* (W.Smith) Kuntze | ***X*** | ***–*** | ***–*** | ***X*** | ***–*** |  | ***–*** |  |
| 507 | *Homoeocladia distans* (W.Gregory) Kuntze | ***X*** | ***–*** | ***–*** | ***–*** | ***X*** |  | ***–*** |  |
| 508 | *Humidophila contenta* (Grunow) R.L.Lowe, Kociolek, Johansen, Van de Vijver, Lange-Bertalot et Kopalova | ***–*** | ***X*** | ***X*** | ***–*** | ***–*** |  | ***–*** |  |
| 509 | *Hyalodiscus radiatus* (O'Meara) Grunow | ***X*** | ***–*** | ***–*** | ***X*** | ***X*** |  | ***–*** |  |
| 510 | *Hyalodiscus* spp*.* Ehrenberg | ***X*** | ***–*** | ***–*** | ***–*** | ***–*** | ***X (Bryozoa)*** | ***–*** |  |
| 511 | *Hyalosinedra* spp*.* D.M.Williams et Round | ***X*** | ***–*** | ***–*** | ***X*** | ***–*** |  | ***–*** |  |
| 512 | *Hyalosira faba* Majewska | ***X*** | ***–*** | ***X*** | ***–*** | ***–*** |  | ***–*** |  |
| 513 | *Hyalosira navarroana* Lobban et Majewska | ***X*** | ***–*** | ***X*** | ***–*** | ***–*** |  | ***–*** |  |
| 514 | *Hyalosira* spp*.* Kützing | ***X*** | ***–*** | ***X*** | ***–*** | ***–*** |  | ***–*** |  |
| 515 | *Hydrosera whampoensis* (Schwarz) Deby | ***–*** | ***X*** | ***–*** | ***X*** | ***–*** |  | ***–*** |  |
| 516 | *Iconella linearis* (W.Smith) Ruck et Nakov | ***–*** | ***X*** | ***X*** | ***–*** | ***–*** |  | ***–*** |  |
| 517 | *Isthmia enervis* Ehrenberg | ***X*** | ***–*** | ***X*** | ***–*** | ***–*** |  | ***–*** |  |
| 518 | *Karayevia amoena* (Hustedt) Bukhtiyarova | ***X*** | ***–*** | ***–*** | ***–*** | ***X*** |  | ***–*** |  |
| 519 | *Karayevia clevei* (Grunow) Bukhtiyarova | ***–*** | ***X*** | ***X*** | ***–*** | ***X*** |  | ***–*** |  |
| 520 | *Karayevia ploenensis* (Hustedt) Bukhtiyarova | ***–*** | ***X*** | ***X*** | ***–*** | ***–*** |  | ***–*** |  |
| 521 | *Karayevia submarina* (Hustedt) Bukhtiyarova | ***X*** | ***–*** | ***X*** | ***–*** | ***–*** |  | ***–*** |  |
| 522 | *Labellicula lecohuiana* Majewska et Van de Vijver | ***X*** | ***–*** | ***X*** | ***–*** | ***–*** |  | ***–*** |  |
| 523 | *Lauderia annulata* C.Agardh | ***X*** | ***–*** | ***–*** | ***X*** | ***–*** |  | ***–*** |  |
| 524 | *Lemnicola hungarica* (Grunow) Round et Basson | ***–*** | ***X*** | ***X*** | ***X*** | ***–*** |  | ***–*** |  |
| 525 | *Leptocylindrus minimus* Gran | ***X*** | ***–*** | ***X*** | ***–*** | ***–*** |  | ***–*** |  |
| 526 | *Licmophora abbreviata* C.Agardh | ***X*** | ***–*** | ***X*** | ***X*** | ***–*** | ***X (Cnidaria)*** | ***–*** |  |
| 527 | *Licmophora cf. abbreviata* C.Agardh | ***–*** | ***–*** | ***–*** | ***–*** | ***X*** |  | ***–*** |  |
| 528 | *Licmophora cf. communis* (Heiberg) Grunow | ***X*** | ***–*** | ***–*** | ***–*** | ***–*** | ***X (Cnidaria)*** | ***–*** |  |
| 529 | *Licmophora cf. dalmatica* (Kützing) Grunow | ***X*** | ***–*** | ***–*** | ***–*** | ***–*** | ***X (Cnidaria)*** | ***–*** |  |
| 530 | *Licmophora communis* (Heiberg) Grunow | ***X*** | ***–*** | ***–*** | ***–*** | ***–*** | ***X (Bryozoa)*** | ***–*** |  |
| 531 | *Licmophora ehrenbergii* (Kützing) Grunow | ***X*** | ***–*** | ***X*** | ***–*** | ***X*** |  | ***–*** |  |
| 532 | *Licmophora flabellata* (Carmichael) C.Agardh | ***X*** | ***–*** | ***X*** | ***–*** | ***X*** | ***X (Cnidaria)*** | ***–*** |  |
| 533 | *Licmophora gracilis* (Ehrenberg) Grunow | ***X*** | ***–*** | ***–*** | ***X*** | ***X*** |  | ***–*** |  |
| 534 | *Licmophora hyalina* (Kützing) Grunow | ***X*** | ***–*** | ***X*** | ***–*** | ***X*** |  | ***–*** |  |
| 535 | *Licmophora lyngbyei* (Kützing) Grunow | ***X*** | ***–*** | ***–*** | ***–*** | ***X*** |  | ***–*** |  |
| 536 | *Licmophora onassis* Hustedt | ***X*** | ***–*** | ***X*** | ***–*** | ***–*** |  | ***–*** |  |
| 537 | *Licmophora reichardtii* Grunow | ***X*** | ***–*** | ***–*** | ***–*** | ***–*** | ***X (Cnidaria)*** | ***–*** |  |
| 538 | *Licmophora remulus* Grunow | ***X*** | ***–*** | ***X*** | ***–*** | ***–*** |  | ***–*** |  |
| 539 | *Licmophora* spp*.* C.Agardh | ***X*** | ***–*** | ***X*** | ***X*** | ***X*** | ***X (Cnidaria, Bryozoa)*** | ***–*** |  |
| 540 | *Licmophora tenuis* (Kützing) Grunow | ***X*** | ***–*** | ***–*** | ***–*** | ***X*** |  | ***–*** |  |
| 541 | *Licmophora unidenticulata* Takano | ***X*** | ***–*** | ***–*** | ***–*** | ***X*** |  | ***–*** |  |
| 542 | *Licmphora oedipus* (Kützing) Grunow | ***X*** | ***–*** | ***–*** | ***–*** | ***–*** | ***X (Cnidaria)*** | ***–*** |  |
| 543 | *Lithodesmium undulatum* Ehrenberg | ***X*** | ***–*** | ***–*** | ***–*** | ***–*** | ***X (Bryozoa)*** | ***–*** |  |
| 544 | *Lucanicum ashworthianum*^[1][2]^  *Majewska, K.Robert et Van de Vijver* |  |  | ***X*^[1]^** |  |  |  | **^[1][2]^Testudines (*Caretta caretta*)** | **Majewska *et al*. (2019)** |
| 545 | *Lutica monita* (Hustedt) D.G.Mann | ***–*** | ***X*** | ***–*** | ***X*** | ***–*** |  | ***–*** |  |
| 546 | *Luticola cf. goeppertiana* (Bleisch) D.G.Mann | ***–*** | ***X*** | ***X*** | ***–*** | ***–*** |  | ***–*** |  |
| 547 | *Luticola cf. mutica* (Kützing) D.G.Mann | ***–*** | ***X*** | ***X*** | ***–*** | ***–*** |  | ***–*** |  |
| 548 | *Luticola dapalis* (Frenguelli) D.G.Mann | ***–*** | ***X*** | ***–*** | ***X*** | ***–*** |  | ***–*** |  |
| 549 | *Luticola dapaloides* (Fenguelli) Metzeltin et Lange-Bertalot | ***–*** | ***X*** | ***–*** | ***X*** | ***–*** |  | ***–*** |  |
| 550 | *Luticola goeppertiana* (Bleisch) D.G.Mann | ***–*** | ***X*** | ***X*** | ***X*** | ***–*** |  | ***–*** |  |
| 551 | *Luticola mutica* (Kützing) D.G.Mann | ***X*** | ***X*** | ***X*** | ***X*** | ***–*** |  | ***–*** |  |
| 552 | *Luticola nivalis* (Ehrenberg) D.G.Mann | ***–*** | ***X*** | ***X*** | ***–*** | ***–*** |  | ***–*** |  |
| 553 | *Luticola saprophila* Levkov, Metzeltin et Pavlov | ***–*** | ***X*** | ***X*** | ***–*** | ***–*** |  | ***–*** |  |
| 554 | *Luticola* spp*.* D.G.Mann | ***–*** | ***X*** | ***X*** | ***–*** | ***–*** |  | ***–*** |  |
| 555 | *Luticola ventriconfusa* Lange-Bertalot | ***–*** | ***X*** | ***X*** | ***–*** | ***–*** |  | ***–*** |  |
| 556 | *Lyrella exsul* (A.W.F.Schmidt) D.G.Mann | ***X*** | ***–*** | ***–*** | ***–*** | ***X*** |  | ***–*** |  |
| 557 | *Lyrella lyra* (Ehrenberg) Karayeva | ***X*** | ***–*** | ***–*** | ***–*** | ***–*** | ***X (Bryozoa)*** | ***–*** |  |
| 558 | *Mastogloia acutiuscula* Grunow | ***X*** | ***–*** | ***–*** | ***–*** | ***X*** |  | ***–*** |  |
| 559 | *Mastogloia angulata* F.W.Lewis | ***X*** | ***–*** | ***–*** | ***–*** | ***X*** |  | ***–*** |  |
| 560 | *Mastogloia apiculata* W.Smith | ***X*** | ***–*** | ***–*** | ***–*** | ***X*** |  | ***–*** |  |
| 561 | *Mastogloia binotata* (Grunow) Cleve | ***X*** | ***–*** | ***X*** | ***–*** | ***X*** |  | ***–*** |  |
| 562 | *Mastogloia braunii* Grunow | ***X*** | ***–*** | ***–*** | ***–*** | ***X*** |  | ***–*** |  |
| 563 | *Mastogloia crucicula* (Grunow) Cleve | ***X*** | ***–*** | ***X*** | ***–*** | ***–*** |  | ***–*** |  |
| 564 | *Mastogloia crucicula var. alternans* Zanon | ***X*** | ***–*** | ***X*** | ***–*** | ***–*** |  | ***–*** |  |
| 565 | *Mastogloia cuneata* (F.Meister) Simonsen | ***X*** | ***–*** | ***X*** | ***–*** | ***–*** |  | ***–*** |  |
| 566 | *Mastogloia exigua* F.W.Lewis | ***X*** | ***–*** | ***–*** | ***–*** | ***X*** |  | ***–*** |  |
| 567 | *Mastogloia gieskesii* Cholnoky | ***X*** | ***–*** | ***–*** | ***–*** | ***X*** |  | ***–*** |  |
| 568 | *Mastogloia hormuzensis*^[1][2]^  Pennesi, T.Romagnoli et De Stefano | ***X*** | ***–*** | ***X*** | ***–*** | ***–*** |  | **^[1][2]^Testudines (*Chelonia mydas*)** | **Pennesi *et al*. (2023)** |
| 569 | *Mastogloia minuta* Greville | ***X*** | ***–*** | ***–*** | ***X*** | ***–*** |  | ***–*** |  |
| 570 | *Mastogloia paradoxa*  Grunow | ***X*** | ***–*** | ***–*** | ***X*** | ***–*** |  | ***–*** |  |
| 571 | *Mastogloia pisciculus* Cleve | ***X*** | ***–*** | ***–*** | ***–*** | ***X*** |  | ***–*** |  |
| 572 | *Mastogloia pusilla* (Grunow) Cleve | ***X*** | ***–*** | ***–*** | ***X*** | ***–*** |  | ***–*** |  |
| 573 | *Mastogloia robusta* Hustedt | ***X*** | ***–*** | ***–*** | ***–*** | ***X*** |  | ***–*** |  |
| 574 | *Mastogloia schmidtii* Heiden | ***X*** | ***–*** | ***X*** | ***–*** | ***–*** |  | ***–*** |  |
| 575 | *Mastogloia sterijovskii*^[1][2]^ Pavlov, Jovanovska, Wetzel, Levkov et Ector | ***–*** | ***X*** | ***X*** | ***–*** | ***–*** |  | **^[1][2]^Testudines (*Emys orbicularis*)** | **Pavlov *et al*. (2016)** |
| 576 | *Mastogloia splendida* (W.Gregory) Cleve et Mӧller | ***X*** | ***–*** | ***X*** | ***–*** | ***–*** |  | ***–*** |  |
| 577 | *Mastogloia* spp*.* Thwaites | ***X*** | ***X*** | ***–*** | ***X*** | ***X*** |  | ***–*** |  |
| 578 | *Mastogloia undulata* Grunow | ***X*** | ***–*** | ***–*** | ***–*** | ***–*** | ***X (Cnidaria)*** | ***–*** |  |
| 579 | *Mayamaea atomus* (Kützing) Lange-Bertalot | ***–*** | ***X*** | ***X*** | ***–*** | ***–*** |  | ***–*** |  |
| 580 | *Mayamaea cahabaensis* E.A.Morales et K.M.Manoylov | ***–*** | ***X*** | ***–*** | ***–*** | ***X*** |  | ***–*** |  |
| 581 | *Mayamaea excelsa* (Krasske) Lange-Bertalot | ***–*** | ***X*** | ***X*** | ***–*** | ***–*** |  | ***–*** |  |
| 582 | *Mayamaea permitis* (Hustedt) Bruder et Medlin | ***–*** | ***X*** | ***X*** | ***–*** | ***X*** |  | ***–*** |  |
| 583 | *Medlinella amphoroidea*^[1][2]^ Frankovich, Ashworth et M.J.Sullivan | ***X*** | ***–*** | ***X*^[1]^** | ***–*** | ***–*** |  | **^[2]^Testudines (*Caretta caretta*)** | **Frankovich *et al*. (2016)** |
| 584 | *Medlinella* spp*^.^*^[1][2]^ Frankovich, Ashworth et M.J.Sullivan | ***–*** | ***–*** | ***X*^[1]^** | ***–*** | ***–*** |  | **^[2]^Testudines (*Caretta caretta*)** | **Frankovich *et al*. (2016)** |
| 585 | *Meloneis mimallis* Louvrou, Danielidis et Economou-Amili | ***X*** | ***–*** | ***X*** | ***–*** | ***–*** |  | ***–*** |  |
| 586 | *Melosira distans f. lirata* (Ehrenberg) O.F.Müller | ***X*** | ***–*** | ***–*** | ***–*** | ***X*** |  | ***–*** |  |
| 587 | *Melosira hyperborea* Grunow | ***X*** | ***–*** | ***–*** | ***X*** | ***–*** |  | ***–*** |  |
| 588 | *Melosira italica* (Ehrenberg) Kützing | ***–*** | ***X*** | ***X*** | ***–*** | ***–*** |  | ***–*** |  |
| 589 | *Melosira moniliformis* (O.F.Müller) C.Agardh | ***X*** | ***–*** | ***–*** | ***X*** | ***X*** | ***X (Bryozoa)*** | ***–*** |  |
| 590 | *Melosira nummuloides* C.Agardh | ***X*** | ***–*** | ***–*** | ***X*** | ***X*** |  | ***–*** |  |
| 591 | *Melosira sol* (Ehrenberg) Kützing | ***X*** | ***–*** | ***X*** | ***–*** | ***–*** |  | ***–*** |  |
| 592 | *Melosira* spp*.* C.Agardh | ***X*** | ***X*** | ***X*** | ***X*** | ***X*** | ***X (Bryozoa)*** | ***–*** |  |
| 593 | *Melosira varians* C.Agardh | ***–*** | ***X*** | ***X*** | ***X*** | ***X*** |  | ***–*** |  |
| 594 | *Melosira westii var. quadrata* W.Smith | ***X*** | ***–*** | ***–*** | ***–*** | ***X*** |  | ***–*** |  |
| 595 | *Meridion circulare* (Greville) C.Agardh | ***–*** | ***X*** | ***–*** | ***–*** | ***X*** |  | ***–*** |  |
| 596 | *Meridion circulare var. constrictum* (Ralfs) Van Heurck | ***–*** | ***X*** | ***–*** | ***–*** | ***X*** |  | ***–*** |  |
| 597 | *Meuniera membranacea* (Cleve) P.C.Silva | ***X*** | ***–*** | ***–*** | ***X*** | ***–*** |  | ***–*** |  |
| 598 | *Microtabella interrupta* (Ehrenberg) Round | ***X*** | ***–*** | ***X*** | ***–*** | ***–*** |  | ***–*** |  |
| 599 | *Microtabella* spp*.* Round | ***X*** | ***–*** | ***X*** | ***–*** | ***–*** |  | ***–*** |  |
| 600 | *Minidiscus chilensis* P.Rivera | ***X*** | ***–*** | ***–*** | ***X*** | ***–*** |  | ***–*** |  |
| 601 | *Nagumoea hydrophicola*^[2]^ Majewska | ***X*** | ***–*** | ***X*** | ***–*** | ***X*** |  | **^[2]^Squamata (*Hydrophis platarus*)** | **Majewska (2021)** |
| 602 | *Nagumoea* spp.*^.^* Kociolek et Witkowski | ***–*** | ***–*** | ***X*** | ***–*** | ***–*** |  | ***–*** |  |
| 603 | *Navicula abunda* Hustedt | ***X*** | ***–*** | ***–*** | ***–*** | ***X*** |  | ***–*** |  |
| 604 | *Navicula accomoda* Hustedt | ***–*** | ***X*** | ***–*** | ***–*** | ***X*** |  | ***–*** |  |
| 605 | *Navicula agnita* Hustedt | ***X*** | ***–*** | ***–*** | ***X*** | ***–*** |  | ***–*** |  |
| 606 | *Navicula aleksandrae* Lange-Bertalot, Bogaczewicz-Adamczak et Witkowski | ***X*** | ***–*** | ***–*** | ***X*** | ***–*** |  | ***–*** |  |
| 607 | *Navicula ammophila* Grunow | ***X*** | ***–*** | ***X*** | ***–*** | ***–*** |  | ***–*** |  |
| 608 | *Navicula amphiceropsis* Lange-Bertalot et U.Rumrich | ***–*** | ***X*** | ***X*** | ***–*** | ***–*** |  | ***–*** |  |
| 609 | *Navicula antonii* Lange-Bertalot | ***–*** | ***X*** | ***X*** | ***–*** | ***X*** |  | ***–*** |  |
| 610 | *Navicula arenaria* Donkin | ***X*** | ***–*** | ***X*** | ***X*** | ***–*** |  | ***–*** |  |
| 611 | *Navicula associata* Lange-Bertalot | ***–*** | ***X*** | ***X*** | ***–*** | ***–*** |  | ***–*** |  |
| 612 | *Navicula bacillum* Ehrenberg | ***–*** | ***X*** | ***X*** | ***–*** | ***–*** |  | ***–*** |  |
| 613 | *Navicula biskanterae* Hustedt | ***X*** | ***–*** | ***–*** | ***X*** | ***–*** |  | ***–*** |  |
| 614 | *Navicula cancellata* Donkin | ***X*** | ***–*** | ***X*** | ***–*** | ***X*** |  | ***–*** |  |
| 615 | *Navicula capitata var. hungarica* (Grunow) R.Ross | ***–*** | ***X*** | ***–*** | ***–*** | ***X*** |  | ***–*** |  |
| 616 | *Navicula capitatoradiata* H.Germain | ***–*** | ***X*** | ***X*** | ***–*** | ***X*** |  | ***–*** |  |
| 617 | *Navicula capitellata* Skvortzow | ***–*** | ***X*** | ***X*** | ***–*** | ***–*** |  | ***–*** |  |
| 618 | *Navicula caterva* Hohn et Hellerman | ***–*** | ***X*** | ***X*** | ***–*** | ***X*** |  | ***–*** |  |
| 619 | *Navicula cf. borowkae* Witkowski, Lange-Bertalot et Metzeltin | ***X*** | ***–*** | ***X*** | ***–*** | ***–*** |  | ***–*** |  |
| 620 | *Navicula cf. consentanea* Hustedt | ***X*** | ***–*** | ***–*** | ***–*** | ***–*** | ***X (Cnidaria)*** | ***–*** |  |
| 621 | *Navicula cf. directa* (W.Smith) Ralfs | ***X*** | ***–*** | ***–*** | ***X*** | ***X*** |  | ***–*** |  |
| 622 | *Navicula cf. phyllepta* Kützing | ***X*** | ***–*** | ***–*** | ***X*** | ***–*** |  | ***–*** |  |
| 623 | *Navicula cf. rusticensis* Lobban | ***X*** | ***–*** | ***X*** | ***–*** | ***–*** |  | ***–*** |  |
| 624 | *Navicula cf. salinicola* Hustedt | ***X*** | ***–*** | ***–*** | ***X*** | ***–*** |  | ***–*** |  |
| 625 | *Navicula cf. veneta* Kützing | ***X*** | ***–*** | ***–*** | ***X*** | ***–*** |  | ***–*** |  |
| 626 | *Navicula cincta* (Ehrenberg) Ralfs | ***X*** | ***X*** | ***–*** | ***X*** | ***X*** |  | ***–*** |  |
| 627 | *Navicula clementis* Grunow | ***–*** | ***X*** | ***X*** | ***–*** | ***–*** |  | ***–*** |  |
| 628 | *Navicula cryptocephala* Kützing | ***–*** | ***X*** | ***X*** | ***–*** | ***X*** |  | ***–*** |  |
| 629 | *Navicula cryptofallax* Lange-Bertalot et Hofmann | ***–*** | ***X*** | ***–*** | ***–*** | ***X*** |  | ***–*** |  |
| 630 | *Navicula cryptotenella* Lange-Bertalot | ***–*** | ***X*** | ***X*** | ***X*** | ***X*** |  | ***–*** |  |
| 631 | *Navicula cryptotenelloides* Lange-Bertalot | ***–*** | ***X*** | ***X*** | ***–*** | ***X*** |  | ***–*** |  |
| 632 | *Navicula cuspidata* Kützing | ***–*** | ***X*** | ***X*** | ***–*** | ***–*** |  | ***–*** |  |
| 633 | *Navicula cuspidata var. major* F.Meister | ***–*** | ***X*** | ***–*** | ***–*** | ***X*** |  | ***–*** |  |
| 634 | *Navicula dermochelycola*^[1][2]^ Witkowski, Kociolek et Riaux-Gobin | ***X*** | ***–*** | ***X*** | ***–*** | ***–*** |  | **^[1][2]^Testudines (*Lepidochelys olivacea*; *Dermochelys coriacea*)** | **Riaux-Gobin *et al*. (2020)** |
| 635 | *Navicula dicephala* Ehrenberg | ***X*** | ***–*** | ***–*** | ***–*** | ***X*** |  | ***–*** |  |
| 636 | *Navicula directa* (W.Smith) Ralfs | ***X*** | ***–*** | ***–*** | ***–*** | ***X*** |  | ***–*** |  |
| 637 | *Navicula diserta* Hustedt | ***X*** | ***–*** | ***–*** | ***X*** | ***–*** |  | ***–*** |  |
| 638 | *Navicula divergens* (W.Smith) Grunow | ***X*** | ***–*** | ***–*** | ***–*** | ***X*** |  | ***–*** |  |
| 639 | *Navicula elegans* W.Smith | ***–*** | ***X*** | ***X*** | ***–*** | ***–*** |  | ***–*** |  |
| 640 | *Navicula erifuga* Lange-Bertalot | ***–*** | ***X*** | ***X*** | ***–*** | ***–*** |  | ***–*** |  |
| 641 | *Navicula exigua* W.Gregory | ***–*** | ***X*** | ***X*** | ***–*** | ***–*** |  | ***–*** |  |
| 642 | *Navicula germainii* J.H.Wallace | ***–*** | ***X*** | ***X*** | ***–*** | ***–*** |  | ***–*** |  |
| 643 | *Navicula gibbula* Cleve | ***–*** | ***X*** | ***X*** | ***–*** | ***–*** |  | ***–*** |  |
| 644 | *Navicula gregaria* Donkin | ***–*** | ***X*** | ***X*** | ***–*** | ***X*** |  | ***–*** |  |
| 645 | *Navicula halinae* Witkowski, Lange-Bertalot et Metzeltin | ***X*** | ***–*** | ***X*** | ***–*** | ***–*** |  | ***–*** |  |
| 646 | *Navicula hamiltonii* Witkowski, Lange-Bertalot et Metzeltin | ***X*** | ***–*** | ***–*** | ***X*** | ***–*** |  | ***–*** |  |
| 647 | *Navicula heufleri* Grunow | ***–*** | ***X*** | ***–*** | ***–*** | ***X*** |  | ***–*** |  |
| 648 | *Navicula humerosa* Brébisson | ***X*** | ***–*** | ***–*** | ***–*** | ***X*** |  | ***–*** |  |
| 649 | *Navicula lanceolata* (C.Agardh) Ehrenberg | ***–*** | ***X*** | ***X*** | ***–*** | ***X*** |  | ***–*** |  |
| 650 | *Navicula longa* (W.Gregory) Ralfs | ***X*** | ***–*** | ***–*** | ***–*** | ***X*** |  | ***–*** |  |
| 651 | *Navicula lundii* E.Reichardt | ***–*** | ***X*** | ***X*** | ***–*** | ***–*** |  | ***–*** |  |
| 652 | *Navicula lyra* Ehrenberg | ***X*** | ***–*** | ***–*** | ***–*** | ***X*** |  | ***–*** |  |
| 653 | *Navicula menisculus* Schumann | ***–*** | ***X*** | ***X*** | ***–*** | ***–*** |  | ***–*** |  |
| 654 | *Navicula metareichardtiana* Lange-Bertalot et Krusber | ***–*** | ***X*** | ***X*** | ***–*** | ***–*** |  | ***–*** |  |
| 655 | *Navicula minuscula* Grunow | ***–*** | ***X*** | ***X*** | ***–*** | ***–*** |  | ***–*** |  |
| 656 | *Navicula normaloides* Cholnoky | ***X*** | ***–*** | ***X*** | ***–*** | ***X*** |  | ***–*** |  |
| 657 | *Navicula normaloides var. irregularis* Cholnoky | ***X*** | ***–*** | ***–*** | ***–*** | ***X*** |  | ***–*** |  |
| 658 | *Navicula notabilis* Greville | ***X*** | ***–*** | ***–*** | ***–*** | ***X*** |  | ***–*** |  |
| 659 | *Navicula novaesiberica* Lange-Bertalot | ***–*** | ***X*** | ***X*** | ***–*** | ***X*** |  | ***–*** |  |
| 660 | *Navicula oscitans var. curvipunctata* (Leuduger-Fortmorel) Frenguelli | ***X*** | ***–*** | ***–*** | ***–*** | ***X*** |  | ***–*** |  |
| 661 | *Navicula palpebralis var. angulosa* (W.Gregory) Van Heurck | ***X*** | ***–*** | ***X*** | ***–*** | ***–*** |  | ***–*** |  |
| 662 | *Navicula pennata* A.W.F.Schmidt | ***X*** | ***–*** | ***–*** | ***–*** | ***X*** |  | ***–*** |  |
| 663 | *Navicula perminuta* Grunow | ***X*** | ***–*** | ***X*** | ***–*** | ***–*** |  | ***–*** |  |
| 664 | *Navicula permulsa* Hustedt | ***X*** | ***–*** | ***X*** | ***–*** | ***–*** |  | ***–*** |  |
| 665 | *Navicula perrhombus* Hustedt | ***X*** | ***–*** | ***–*** | ***X*** | ***–*** |  | ***–*** |  |
| 666 | *Navicula phyllepta* Kützing | ***X*** | ***–*** | ***–*** | ***X*** | ***–*** |  | ***–*** |  |
| 667 | *Navicula placentula* (Ehrenberg) Kützing | ***X*** | ***–*** | ***–*** | ***–*** | ***X*** |  | ***–*** |  |
| 668 | *Navicula platyventris* F.Meister | ***X*** | ***–*** | ***–*** | ***–*** | ***X*** |  | ***–*** |  |
| 669 | *Navicula praeterita* Hustedt | ***–*** | ***X*** | ***–*** | ***–*** | ***X*** |  | ***–*** |  |
| 670 | *Navicula pungens* A.Cleve | ***X*** | ***–*** | ***–*** | ***X*** | ***–*** |  | ***–*** |  |
| 671 | *Navicula pupula var. eliptica* Hustedt | ***–*** | ***X*** | ***–*** | ***–*** | ***X*** |  | ***–*** |  |
| 672 | *Navicula radiosa* Kützing | ***–*** | ***X*** | ***X*** | ***–*** | ***X*** |  | ***–*** |  |
| 673 | *Navicula ramosissima* (C.Agardh) Cleve | ***X*** | ***–*** | ***–*** | ***X*** | ***–*** |  | ***–*** |  |
| 674 | *Navicula recurva* (F.Meister) Witkowski, Lange-Bertalot et Metzeltin | ***X*** | ***–*** | ***–*** | ***X*** | ***–*** |  | ***–*** |  |
| 675 | *Navicula reichardtiana* Lange-Bertalot | ***–*** | ***X*** | ***–*** | ***–*** | ***X*** |  | ***–*** |  |
| 676 | *Navicula retrocurvata* J.R.Carter | ***X*** | ***–*** | ***–*** | ***–*** | ***–*** | ***X (Annelida)*** | ***–*** |  |
| 677 | *Navicula rhynchocephala* Kützing | ***–*** | ***X*** | ***X*** | ***–*** | ***X*** |  | ***–*** |  |
| 678 | *Navicula rostellata* Kützing | ***X*** | ***X*** | ***X*** | ***–*** | ***X*** |  | ***–*** |  |
| 679 | *Navicula rusticensis* Lobban | ***X*** | ***–*** | ***X*** | ***–*** | ***–*** |  | ***–*** |  |
| 680 | *Navicula salinarum* Grunow | ***X*** | ***–*** | ***–*** | ***X*** | ***–*** |  | ***–*** |  |
| 681 | *Navicula simulata* Manguin | ***–*** | ***X*** | ***X*** | ***–*** | ***–*** |  | ***–*** |  |
| 682 | *Navicula spartinetensis* Sullivan et Reimer | ***X*** | ***–*** | ***–*** | ***X*** | ***–*** |  | ***–*** |  |
| 683 | *Navicula splendida* Van Landingham | ***–*** | ***X*** | ***–*** | ***–*** | ***X*** |  | ***–*** |  |
| 684 | *Navicula* spp*.* Bory | ***X*** | ***X*** | ***X*** | ***X*** | ***X*** | ***X (Bryozoa, Cnidaria, Annelida, Porifera)*** | ***–*** |  |
| 685 | *Navicula subagnita* Proschkinia-Lavrenko | ***X*** | ***–*** | ***–*** | ***X*** | ***–*** |  | ***–*** |  |
| 686 | *Navicula subrhynchocephala* Hustedt | ***–*** | ***–*** | ***–*** | ***X*** | ***–*** |  | ***–*** |  |
| 687 | *Navicula symmetrica* R.M.Patrick | ***–*** | ***X*** | ***–*** | ***X*** | ***–*** |  | ***–*** |  |
| 688 | *Navicula tenelloides* Hustedt | ***–*** | ***X*** | ***X*** | ***–*** | ***–*** |  | ***–*** |  |
| 689 | *Navicula transitans* Cleve | ***X*** | ***–*** | ***X*** | ***–*** | ***–*** |  | ***–*** |  |
| 690 | *Navicula tripunctata* (O.F.Müller) Bory | ***X*** | ***X*** | ***X*** | ***–*** | ***X*** |  | ***–*** |  |
| 691 | *Navicula trivialis* Lange-Bertalot | ***–*** | ***X*** | ***X*** | ***–*** | ***–*** |  | ***–*** |  |
| 692 | *Navicula tsukamotoi* (Sterrenburg et Hinz) Yuhang Li et Kuidong Xu | ***X*** | ***–*** | ***–*** | ***X*** | ***–*** |  | ***–*** |  |
| 693 | *Navicula tuscula* Ehrenberg | ***–*** | ***X*** | ***X*** | ***–*** | ***–*** |  | ***–*** |  |
| 694 | *Navicula upsaliensis* (Grunow) M.Peragallo | ***–*** | ***X*** | ***X*** | ***–*** | ***–*** |  | ***–*** |  |
| 695 | *Navicula vandamii* Schoeman et R.E.M.Archibald | ***–*** | ***X*** | ***X*** | ***–*** | ***–*** |  | ***–*** |  |
| 696 | *Navicula vekhovii* Lange-Bertalot et Genkal | ***–*** | ***–*** | ***–*** | ***X*** | ***–*** |  | ***–*** |  |
| 697 | *Navicula veneta* Kützing | ***–*** | ***X*** | ***X*** | ***X*** | ***X*** |  | ***–*** |  |
| 698 | *Navicula vilaplanii* (Lange-Bertalot et Sabater) Lange-Bertalot et Sabater | ***–*** | ***X*** | ***X*** | ***–*** | ***–*** |  | ***–*** |  |
| 699 | *Navicula viminoides* Giffen | ***X*** | ***–*** | ***X*** | ***X*** | ***–*** |  | ***–*** |  |
| 700 | *Navicula viridis* (Nitzsch) Ehrenberg | ***X*** | ***–*** | ***X*** | ***–*** | ***–*** |  | ***–*** |  |
| 701 | *Navicula viridula* (Kützing) Ehrenberg | ***–*** | ***X*** | ***X*** | ***–*** | ***–*** |  | ***–*** |  |
| 702 | *Navicymbula pusilla var. lata* Krammer | ***X*** | ***–*** | ***–*** | ***–*** | ***X*** |  | ***–*** |  |
| 703 | *Neidium dubium* (Ehrenberg) Cleve | ***–*** | ***X*** | ***X*** | ***–*** | ***–*** |  | ***–*** |  |
| 704 | *Neosynedra provincialis* (Grunow) D.M.Williams et Round | ***X*** | ***–*** | ***X*** | ***–*** | ***–*** |  | ***–*** |  |
| 705 | *Neosynedra* spp*.* D.M.Williams et Round | ***X*** | ***–*** | ***X*** | ***–*** | ***–*** |  | ***–*** |  |
| 706 | *Nitzschia acicularis* (Kützing) W.Smith | ***–*** | ***X*** | ***–*** | ***–*** | ***X*** |  | ***–*** |  |
| 707 | *Nitzschia aff. dissipata* (Kützing) Grunow | ***X*** | ***–*** | ***–*** | ***X*** | ***–*** |  | ***–*** |  |
| 78 | *Nitzschia aff. frustulum* (Kützing) Grunow | ***X*** | ***–*** | ***–*** | ***X*** | ***X*** |  | ***–*** |  |
| 709 | *Nitzschia aff. hybrida* Grunow | ***X*** | ***–*** | ***–*** | ***–*** | ***X*** |  | ***–*** |  |
| 710 | *Nitzschia aff. inconspicua* Grunow | ***X*** | ***–*** | ***–*** | ***X*** | ***X*** |  | ***–*** |  |
| 711 | *Nitzschia aff. media* Hantzsch | ***X*** | ***–*** | ***–*** | ***X*** | ***X*** |  | ***–*** |  |
| 712 | *Nitzschia aff. valida* Cleve et Grunow | ***X*** | ***–*** | ***–*** | ***–*** | ***X*** |  | ***–*** |  |
| 713 | *Nitzschia alicae* Hlubikova et Ector | ***–*** | ***X*** | ***–*** | ***–*** | ***X*** |  | ***–*** |  |
| 714 | *Nitzschia amphibia* Grunow | ***–*** | ***X*** | ***X*** | ***X*** | ***X*** |  | ***–*** |  |
| 715 | *Nitzschia amphibia* f. *anormale* Grunow | ***–*** | ***X*** | ***X*** | ***–*** | ***–*** |  | ***–*** |  |
| 716 | *Nitzschia amplectens* Hustedt | ***X*** | ***–*** | ***–*** | ***–*** | ***–*** | ***X (Annelida)*** | ***–*** |  |
| 717 | *Nitzschia angustata* (W.Smith) Grunow | ***–*** | ***X*** | ***–*** | ***–*** | ***X*** |  | ***–*** |  |
| 718 | *Nitzschia apiculata* (W.Gregory) Grunow | ***X*** | ***–*** | ***–*** | ***–*** | ***X*** |  | ***–*** |  |
| 719 | *Nitzschia archibaldii* Lange-Bertalot | ***–*** | ***X*** | ***X*** | ***–*** | ***X*** |  | ***–*** |  |
| 720 | *Nitzschia barkleyi* Hustedt | ***X*** | ***–*** | ***X*** | ***–*** | ***–*** |  | ***–*** |  |
| 721 | *Nitzschia brunoi* Lange-Bertalot | ***–*** | ***X*** | ***–*** | ***–*** | ***X*** |  | ***–*** |  |
| 722 | *Nitzschia capitellata* Hustedt | ***–*** | ***X*** | ***X*** | ***–*** | ***–*** |  | ***–*** |  |
| 723 | *Nitzschia carnicobarica* Desikachary et Prema | ***X*** | ***–*** | ***–*** | ***–*** | ***X*** |  | ***–*** |  |
| 724 | *Nitzschia cf. aequorea* Hustedt | ***X*** | ***–*** | ***–*** | ***X*** | ***–*** |  | ***–*** |  |
| 725 | *Nitzschia cf. amphibioides* Hustedt | ***–*** | ***X*** | ***–*** | ***X*** | ***–*** |  | ***–*** |  |
| 726 | *Nitzschia cf. dubia* W.Smith | ***X*** | ***–*** | ***–*** | ***X*** | ***–*** |  | ***–*** |  |
| 727 | *Nitzschia cf. inconspicua* Grunow | ***X*** | ***–*** | ***X*** | ***–*** | ***–*** |  | ***–*** |  |
| 728 | *Nitzschia cf. lanceolata* W.Smith | ***–*** | ***–*** | ***X*** | ***–*** | ***–*** |  | ***–*** |  |
| 729 | *Nitzschia cf. sigma* (Kützing) W.Smith | ***X*** | ***–*** | ***X*** | ***X*** | ***X*** | ***X (Echinodermata)*** | ***–*** |  |
| 730 | *Nitzschia closterium* (Ehrenberg) W.Smith | ***X*** | ***–*** | ***X*** | ***–*** | ***–*** |  | ***–*** |  |
| 731 | *Nitzschia constricta* (Kützing) Ralfs | ***X*** | ***–*** | ***–*** | ***–*** | ***–*** | ***X (Annelida)*** | ***–*** |  |
| 732 | *Nitzschia costei* Tudesque, Rimet et Ector | ***–*** | ***X*** | ***X*** | ***–*** | ***X*** |  | ***–*** |  |
| 733 | *Nitzschia denticula* Grunow | ***X*** | ***X*** | ***X*** | ***X*** | ***X*** |  | ***–*** |  |
| 734 | *Nitzschia dissipata* (Kützing) Grunow | ***X*** | ***X*** | ***X*** | ***X*** | ***X*** |  | ***–*** |  |
| 735 | *Nitzschia dissipata f. anormale* (Kützing) Grunow | ***–*** | ***X*** | ***X*** | ***–*** | ***–*** |  | ***–*** |  |
| 7367 | *Nitzschia distans* W.Gregory | ***X*** | ***–*** | ***–*** | ***X*** | ***–*** |  | ***–*** |  |
| 737 | *Nitzschia elegantula* Grunow | ***X*** | ***–*** | ***X*** | ***X*** | ***–*** |  | ***–*** |  |
| 738 | *Nitzschia fasciculata* (Grunow) Grunow | ***X*** | ***–*** | ***X*** | ***X*** | ***–*** | ***X (Annelida)*** | ***–*** |  |
| 739 | *Nitzschia flexa* Schumann | ***–*** | ***X*** | ***X*** | ***–*** | ***–*** |  | ***–*** |  |
| 740 | *Nitzschia fonticola* Grunow | ***–*** | ***X*** | ***X*** | ***–*** | ***X*** |  | ***–*** |  |
| 741 | *Nitzschia frustulum* (Kützing) Grunow | ***X*** | ***X*** | ***X*** | ***X*** | ***X*** | ***X (Bryozoa, Annelida)*** | ***–*** |  |
| 742 | *Nitzschia fusoides* Ehrlich | ***X*** | ***–*** | ***–*** | ***–*** | ***X*** |  | ***–*** |  |
| 743 | *Nitzschia gracilis* Hantzsch | ***–*** | ***X*** | ***–*** | ***–*** | ***X*** |  | ***–*** |  |
| 744 | *Nitzschia heufleriana* Grunow | ***–*** | ***X*** | ***X*** | ***–*** | ***X*** |  | ***–*** |  |
| 745 | *Nitzschia inconspicua* Grunow | ***X*** | ***X*** | ***X*** | ***X*** | ***X*** |  | ***–*** |  |
| 746 | *Nitzschia intermedia* Hantzsch | ***–*** | ***X*** | ***X*** | ***–*** | ***–*** |  | ***–*** |  |
| 747 | *Nitzschia kuetzingiana* Hilse | ***X*** | ***–*** | ***–*** | ***–*** | ***X*** | ***X (Annelida)*** | ***–*** |  |
| 748 | *Nitzschia lacuum* Lange-Bertalot | ***–*** | ***X*** | ***–*** | ***–*** | ***X*** |  | ***–*** |  |
| 749 | *Nitzschia liebetruthii* Rabenhorst | ***X*** | ***–*** | ***X*** | ***–*** | ***–*** |  | ***–*** |  |
| 750 | *Nitzschia ligowskii* Lange-Bertalot, Kociolek et Brzezinska | ***X*** | ***–*** | ***–*** | ***–*** | ***X*** |  | ***–*** |  |
| 751 | *Nitzschia linearis* (C.Agardh) W.Smith | ***X*** | ***X*** | ***X*** | ***X*** | ***X*** |  | ***–*** |  |
| 752 | *Nitzschia linearis var. subtilis* (Grunow) Hustedt | ***–*** | ***X*** | ***–*** | ***–*** | ***X*** |  | ***–*** |  |
| 753 | *Nitzschia longissima* (Brébisson) Grunow | ***X*** | ***–*** | ***–*** | ***X*** | ***X*** | ***X (Cnidaria)*** | ***–*** |  |
| 754 | *Nitzschia lorenziana* Grunow | ***X*** | ***–*** | ***–*** | ***X*** | ***–*** |  | ***–*** |  |
| 755 | *Nitzschia media* Hantzsch | ***–*** | ***X*** | ***X*** | ***–*** | ***–*** |  | ***–*** |  |
| 756 | *Nitzschia microcephala* Grunow | ***X*** | ***X*** | ***X*** | ***X*** | ***–*** |  | ***–*** |  |
| 757 | *Nitzschia navicularis* (Brébisson) Grunow | ***X*** | ***–*** | ***–*** | ***–*** | ***–*** | ***X (Bryozoa)*** | ***–*** |  |
| 758 | *Nitzschia obtusa* W.Smith | ***–*** | ***X*** | ***X*** | ***–*** | ***–*** |  | ***–*** |  |
| 759 | *Nitzschia oligotraphenta* (Lange-Bertalot) Lange-Bertalot | ***–*** | ***X*** | ***–*** | ***–*** | ***X*** |  | ***–*** |  |
| 760 | *Nitzschia palea* (Kützing) W.Smith | ***X*** | ***X*** | ***X*** | ***X*** | ***X*** |  | ***–*** |  |
| 761 | *Nitzschia palea var. debilis* (Kützing) Grunow | ***–*** | ***X*** | ***X*** | ***–*** | ***–*** |  | ***–*** |  |
| 762 | *Nitzschia palea var. tenuirostris* Grunow | ***–*** | ***X*** | ***X*** | ***–*** | ***–*** |  | ***–*** |  |
| 763 | *Nitzschia paleacea* (Grunow) Grunow | ***X*** | ***X*** | ***X*** | ***–*** | ***–*** | ***X (Annelida)*** | ***–*** |  |
| 764 | *Nitzschia pararostrata* (Lange-Bertalot) Lange-Bertalot | ***X*** | ***–*** | ***–*** | ***X*** | ***–*** |  | ***–*** |  |
| 765 | *Nitzschia pellucida* Grunow | ***X*** | ***–*** | ***–*** | ***X*** | ***–*** | ***X (Bryozoa)*** | ***–*** |  |
| 766 | *Nitzschia persuadens* Cholnoky | ***X*** | ***–*** | ***–*** | ***–*** | ***X*** |  | ***–*** |  |
| 767 | *Nitzschia pseudocommunis* Hustedt | ***X*** | ***–*** | ***–*** | ***X*** | ***–*** |  | ***–*** |  |
| 768 | *Nitzschia pubens* Cholnoky | ***X*** | ***–*** | ***–*** | ***X*** | ***–*** |  | ***–*** |  |
| 769 | *Nitzschia punctata* (W.Smith) Grunow | ***X*** | ***–*** | ***–*** | ***–*** | ***X*** |  | ***–*** |  |
| 770 | *Nitzschia puriformis* Hlubikova et Ector | ***–*** | ***X*** | ***–*** | ***–*** | ***X*** |  | ***–*** |  |
| 771 | *Nitzschia pusilla* (Kützing) Grunow | ***–*** | ***X*** | ***X*** | ***–*** | ***–*** |  | ***–*** |  |
| 772 | *Nitzschia recta* Hantzsch | ***–*** | ***X*** | ***X*** | ***–*** | ***X*** |  | ***–*** |  |
| 773 | *Nitzschia scalpelliformis* (Grunow) Grunow | ***X*** | ***–*** | ***–*** | ***–*** | ***X*** |  | ***–*** |  |
| 774 | *Nitzschia sigma* (Kützing) W.Smith | ***X*** | ***–*** | ***–*** | ***X*** | ***X*** |  | ***–*** |  |
| 775 | *Nitzschia sigma aff. var. sigmatella* (Kützing) W.Smith | ***X*** | ***–*** | ***–*** | ***X*** | ***X*** |  | ***–*** |  |
| 776 | *Nitzschia sigmoidea* (Nitzsch.) W.Smith | ***–*** | ***X*** | ***X*** | ***–*** | ***X*** |  | ***–*** |  |
| 777 | *Nitzschia sociabilis* Hustedt | ***–*** | ***X*** | ***X*** | ***–*** | ***X*** |  | ***–*** |  |
| 778 | *Nitzschia solgensis* A.Cleve | ***–*** | ***X*** | ***X*** | ***–*** | ***X*** |  | ***–*** |  |
| 779 | *Nitzschia soratensis* E.A.Morales et M.L.Vis | ***–*** | ***X*** | ***X*** | ***–*** | ***X*** |  | ***–*** |  |
| 780 | *Nitzschia species f. anormale* Hassall | ***–*** | ***X*** | ***X*** | ***–*** | ***–*** |  | ***–*** |  |
| 781 | *Nitzschia* spp*.* Hassall | ***X*** | ***X*** | ***X*** | ***X*** | ***X*** | ***X (Annelida)*** | ***–*** |  |
| 782 | *Nitzschia subconstricta* Desikachary et Prema | ***X*** | ***–*** | ***–*** | ***–*** | ***X*** |  | ***–*** |  |
| 783 | *Nitzschia sublinearis* Hustedt | ***–*** | ***X*** | ***X*** | ***–*** | ***–*** |  | ***–*** |  |
| 784 | *Nitzschia subtilis* (Kützing) Grunow | ***X*** | ***–*** | ***–*** | ***–*** | ***X*** |  | ***–*** |  |
| 786 | *Nitzschia supralitorea* Lange-Bertalot | ***–*** | ***X*** | ***X*** | ***–*** | ***–*** |  | ***–*** |  |
| 787 | *Nitzschia tabellaria* (Grunow) Grunow | ***–*** | ***X*** | ***X*** | ***–*** | ***X*** |  | ***–*** |  |
| 788 | *Nitzschia tenuis* W.Smith | ***–*** | ***X*** | ***X*** | ***–*** | ***–*** |  | ***–*** |  |
| 789 | *Nitzschia thermalis* (Kützing) Auerswald | ***X*** | ***–*** | ***–*** | ***–*** | ***X*** |  | ***–*** |  |
| 790 | *Nitzschia tryblionella* Hantzsch | ***X*** | ***–*** | ***–*** | ***–*** | ***X*** |  | ***–*** |  |
| 791 | *Nitzschia tubicola* Østrup | ***X*** | ***–*** | ***X*** | ***–*** | ***–*** |  | ***–*** |  |
| 792 | *Nitzschia valdestriata* Aleem et Hustedt | ***X*** | ***–*** | ***–*** | ***X*** | ***–*** | ***X (Bryozoa)*** | ***–*** |  |
| 793 | *Nitzschia vermicularis* (Kützing) Hantzsch | ***–*** | ***X*** | ***X*** | ***–*** | ***–*** |  | ***–*** |  |
| 794 | *Nitzschia vidovichii* (Grunow) Grunow | ***X*** | ***–*** | ***–*** | ***–*** | ***X*** |  | ***–*** |  |
| 795 | *Nitzschia vitrea* G.Norman | ***X*** | ***–*** | ***–*** | ***X*** | ***–*** |  | ***–*** |  |
| 796 | *Nitzschia volvendirostrata* Ashworth, Dabek et Witkowski | ***X*** | ***–*** | ***X*** | ***–*** | ***–*** |  | ***–*** |  |
| 797 | *Nupela lapidosa* (Krasske) Lange-Bertalot | ***–*** | ***X*** | ***X*** | ***–*** | ***–*** |  | ***–*** |  |
| 798 | *Nupela rumrichorum* Lange-Bertalot | ***–*** | ***X*** | ***X*** | ***–*** | ***–*** |  | ***–*** |  |
| 799 | *Odontella aff. aurita* (Lyngbye) C.Agardh | ***X*** | ***–*** | ***–*** | ***–*** | ***X*** |  | ***–*** |  |
| 800 | *Odontella aurita* (Lyngbye) C.Agardh | ***X*** | ***–*** | ***–*** | ***X*** | ***X*** |  | ***–*** |  |
| 801 | *Odontella obtusa* Kützing | ***X*** | ***–*** | ***–*** | ***X*** | ***X*** |  | ***–*** |  |
| 802 | *Odontella rhombus* (Ehrenberg) Kützing | ***X*** | ***–*** | ***–*** | ***X*** | ***–*** |  | ***–*** |  |
| 803 | *Odontella* spp*.* C.Agardh | ***X*** | ***–*** | ***–*** | ***X*** | ***–*** | ***X (Bryozoa)*** | ***–*** |  |
| 804 | *Odontidium neomaximum* Jüttner, D.M.Williams, Z.Levkov, E.Falasco, M.Battegazzore, Cantonati, Van de Vijver, C.Angele et Ector | ***–*** | ***X*** | ***X*** | ***–*** | ***–*** |  | ***–*** |  |
| 805 | *Odontidium* spp*.* Kützing | ***–*** | ***X*** | ***X*** | ***–*** | ***–*** |  | ***–*** |  |
| 806 | *Olifantiella gorandiana*^[1]^ Riaux-Gobin et Compére | ***X*** | ***–*** | ***X^[1]^*** | ***–*** | ***–*** |  | ***^[1]^Testudines* (*Caretta caretta*; *Lepidochelys olivacea* and *Chelonia mydas*)** |  |
| 807 | *Olifantiella seblae*^[1]^ Kaleli, Krzywda, Witkowski et Solak | ***X*** | ***–*** | ***X^[1]^*** | ***–*** | ***–*** |  | ***^[1]^Testudines* (*Caretta caretta*; *Lepidochelys olivacea* and *Chelonia mydas*)** |  |
| 808 | *Opephora guenter-grassii* (Witkowski et Lange-Bertalot) Sabbe et Vyverman | ***X*** | ***–*** | ***–*** | ***X*** | ***–*** |  | ***–*** |  |
| 809 | *Opephora horstiana* Witkowski | ***X*** | ***–*** | ***–*** | ***X*** | ***–*** |  | ***–*** |  |
| 810 | *Opephora mutabilis* (Grunow) Sabbe et Vyverman | ***X*** | ***–*** | ***–*** | ***–*** | ***X*** |  | ***–*** |  |
| 811 | *Opephora naveana* Le Cohu | ***X*** | ***–*** | ***–*** | ***X*** | ***–*** |  | ***–*** |  |
| 812 | *Opephora pacifica* (Grunow) P.Petit | ***X*** | ***–*** | ***–*** | ***–*** | ***X*** |  | ***–*** |  |
| 813 | *Paralia* spp*.* Heiberg | ***X*** | ***–*** | ***–*** | ***–*** | ***X*** |  | ***–*** |  |
| 814 | *Paralia sulcata* (Ehrenberg) Cleve | ***X*** | ***–*** | ***X*** | ***X*** | ***X*** | ***X (Echinodermata)*** | ***–*** |  |
| 815 | *Parlibellus berkeleyi* (Kützing) J.Cox | ***X*** | ***–*** | ***–*** | ***X*** | ***–*** |  | ***–*** |  |
| 816 | *Parlibellus rhombicula* (Hustedt) Witkowski, Lange-Bertalot et Metzeltin | ***X*** | ***–*** | ***–*** | ***–*** | ***X*** |  | ***–*** |  |
| 817 | *Parlibellus* spp*.* J.Cox | ***X*** | ***–*** | ***X*** | ***X*** | ***X*** |  | ***–*** |  |
| 818 | *Petroneis besarensis* (Giffen) Witkowski et Lange-Bertalot | ***X*** | ***–*** | ***–*** | ***–*** | ***X*** |  | ***–*** |  |
| 819 | *Petroneis granulata* (Bailey) D.G.Mann | ***X*** | ***–*** | ***–*** | ***–*** | ***X*** |  | ***–*** |  |
| 820 | *Pinnularia acrosphaeria* (Brébisson) W.Smith | ***–*** | ***X*** | ***–*** | ***X*** | ***–*** |  | ***–*** |  |
| 821 | *Pinnularia appendiculata* (C.Agardh) Cleve | ***X*** | ***–*** | ***–*** | ***–*** | ***X*** |  | ***–*** |  |
| 822 | *Pinnularia balfouriana* Grunow | ***–*** | ***X*** | ***X*** | ***–*** | ***–*** |  | ***–*** |  |
| 823 | *Pinnularia borealis* Ehrenberg | ***–*** | ***X*** | ***X*** | ***–*** | ***–*** |  | ***–*** |  |
| 824 | *Pinnularia divergens* W.Smith | ***–*** | ***X*** | ***X*** | ***–*** | ***–*** |  | ***–*** |  |
| 825 | *Pinnularia hemiptera* (Kützing) Rabenhorst | ***X*** | ***–*** | ***–*** | ***–*** | ***X*** |  | ***–*** |  |
| 826 | *Pinnularia latarea* Krammer | ***–*** | ***X*** | ***X*** | ***–*** | ***–*** |  | ***–*** |  |
| 827 | *Pinnularia lundii* Hustedt | ***–*** | ***X*** | ***X*** | ***–*** | ***–*** |  | ***–*** |  |
| 828 | *Pinnularia maior* (Kützing) Rabenhorst | ***X*** | ***X*** | ***–*** | ***–*** | ***X*** |  | ***–*** |  |
| 829 | *Pinnularia mesolepta* (Ehrenberg) W.Smith | ***–*** | ***X*** | ***X*** | ***–*** | ***–*** |  | ***–*** |  |
| 830 | *Pinnularia microstauron* (Ehrenberg) Cleve | ***–*** | ***X*** | ***X*** | ***–*** | ***–*** |  | ***–*** |  |
| 831 | *Pinnularia pseudogibba* Krammer | ***–*** | ***X*** | ***–*** | ***–*** | ***X*** |  | ***–*** |  |
| 832 | *Pinnularia quadratarea* (A.W.F.Schmidt) Cleve | ***X*** | ***–*** | ***X*** | ***–*** | ***–*** |  | ***–*** |  |
| 833 | *Pinnularia rectangulata* (W.Gregory) Cleve | ***X*** | ***–*** | ***–*** | ***–*** | ***X*** |  | ***–*** |  |
| 834 | *Pinnularia* spp*.* Ehrenberg | ***X*** | ***X*** | ***X*** | ***X*** | ***X*** |  | ***–*** |  |
| 835 | *Pinnularia stomatophora* (Grunow) Cleve | ***–*** | ***X*** | ***X*** | ***–*** | ***–*** |  | ***–*** |  |
| 836 | *Pinnularia subcapitata var. paucistriata* (Grunow) Cleve | ***–*** | ***X*** | ***–*** | ***–*** | ***X*** |  | ***–*** |  |
| 837 | *Pinnularia viridiformis* Krammer | ***–*** | ***X*** | ***–*** | ***–*** | ***X*** |  | ***–*** |  |
| 838 | *Pinnularia viridis* (Nitzsch) Ehrenberg | ***X*** | ***X*** | ***X*** | ***–*** | ***X*** |  | ***–*** |  |
| 839 | *Pinnularia viridis var. minor* (Nitzsch) Ehrenberg | ***–*** | ***X*** | ***–*** | ***–*** | ***X*** |  | ***–*** |  |
| 840 | *Pinnunavis elegans* (W.Smith) Okuno | ***X*** | ***–*** | ***–*** | ***X*** | ***–*** |  | ***–*** |  |
| 841 | *Pinnunavis yarrensis* (Grunow) Okuno | ***X*** | ***–*** | ***X*** | ***–*** | ***–*** |  | ***–*** |  |
| 842 | *Placoneis anglophila* (Lange-Bertalot) Lange-Bertalot | ***X*** | ***–*** | ***–*** | ***X*** | ***–*** |  | ***–*** |  |
| 843 | *Placoneis elginensis* (W.Gregory) J.Cox | ***–*** | ***X*** | ***X*** | ***–*** | ***–*** |  | ***–*** |  |
| 844 | *Placosira araneosa* Majewska, Ashworth, Shin Sato et Witkowski | ***X*** | ***–*** | ***X*** | ***–*** | ***–*** |  | ***–*** |  |
| 845 | *Plagiogramma minus* (W.Gregory) Chunlian Li, Ashworth et Witkowski | ***X*** | ***–*** | ***–*** | ***–*** | ***X*** |  | ***–*** |  |
| 846 | *Plagiogramma tenuistriatum* Cleve | ***X*** | ***–*** | ***–*** | ***–*** | ***X*** |  | ***–*** |  |
| 847 | *Plagiogrammopsis minima* (Salah) Sabbe et Witkowski | ***X*** | ***–*** | ***–*** | ***X*** | ***–*** |  | ***–*** |  |
| 848 | *Plagiogrammopsis* spp*.* Hasle, Von Stosch et Syvertsen | ***X*** | ***–*** | ***–*** | ***X*** | ***–*** |  | ***–*** |  |
| 849 | *Plagiogrammopsis vanheurckii* (Grunow) Hasle, Stosch et Syvertsen | ***X*** | ***–*** | ***–*** | ***X*** | ***–*** |  | ***–*** |  |
| 850 | *Plagiotropis lepidoptera* (W.Gregory) Kuntze | ***X*** | ***–*** | ***–*** | ***–*** | ***X*** |  | ***–*** |  |
| 851 | *Plagiotropis pusilla* (W.Gregory) Kuntze | ***X*** | ***–*** | ***–*** | ***–*** | ***X*** |  | ***–*** |  |
| 852 | *Plagiotropis* spp*.* Pfitzer | ***X*** | ***–*** | ***–*** | ***–*** | ***–*** | ***X (Porifera)*** | ***–*** |  |
| 853 | *Planothidium cf. delicatulum* (Kützing) Round et Bukhtiyarova | ***X*** | ***–*** | ***X*** | ***–*** | ***–*** |  | ***–*** |  |
| 854 | *Planothidium delicatulum* (Kützing) Round et Bukhtiyarova | ***X*** | ***–*** | ***–*** | ***X*** | ***X*** |  | ***–*** |  |
| 855 | *Planothidium engelbrechtii* (Cholnoky) Round et Bukhtiyarova | ***X*** | ***–*** | ***–*** | ***X*** | ***–*** |  | ***–*** |  |
| 856 | *Planothidium frequentissimum* (Lange-Bertalot) Lange-Bertalot | ***–*** | ***X*** | ***X*** | ***X*** | ***–*** |  | ***–*** |  |
| 857 | *Planothidium incuriatum* Wetzel, Va de Vijver et Ector | ***–*** | ***X*** | ***X*** | ***–*** | ***–*** |  | ***–*** |  |
| 858 | *Planothidium lanceolatum* (Brébisson) Lange-Bertalot | ***–*** | ***X*** | ***X*** | ***–*** | ***X*** |  | ***–*** |  |
| 859 | *Planothidium lilljeborgei* (Grunow) Witkowski et Lange-Bertalot | ***X*** | ***–*** | ***X*** | ***–*** | ***–*** |  | ***–*** |  |
| 860 | *Planothidium* spp*.* Round et Bukhtiyarova | ***X*** | ***–*** | ***X*** | ***X*** | ***X*** |  | ***–*** |  |
| 861 | *Platessa hustedtii* (Krasske) Lange-Bertalot | ***–*** | ***X*** | ***X*** | ***–*** | ***–*** |  | ***–*** |  |
| 862 | *Pleurosigma aff. affine* Grunow | ***X*** | ***–*** | ***X*** | ***–*** | ***X*** |  | ***–*** |  |
| 863 | *Pleurosigma affine* Grunow | ***X*** | ***–*** | ***–*** | ***X*** | ***–*** |  | ***–*** |  |
| 864 | *Pleurosigma angulatum* (J.T.Quekett) W.Smith | ***X*** | ***–*** | ***–*** | ***–*** | ***X*** |  | ***–*** |  |
| 865 | *Pleurosigma cf. angulatum* (J.T.Quekett) W.Smith | ***X*** | ***–*** | ***X*** | ***–*** | ***–*** |  | ***–*** |  |
| 866 | *Pleurosigma delicatulum* W.Smith | ***X*** | ***–*** | ***X*** | ***–*** | ***–*** |  | ***–*** |  |
| 867 | *Pleurosigma distortum* W.Smith | ***X*** | ***–*** | ***–*** | ***–*** | ***X*** |  | ***–*** |  |
| 868 | *Pleurosigma diversestriatum* F.Meister | ***X*** | ***–*** | ***–*** | ***–*** | ***X*** |  | ***–*** |  |
| 869 | *Pleurosigma elongatum* W.Smith | ***–*** | ***–*** | ***–*** | ***X*** | ***–*** |  | ***–*** |  |
| 870 | *Pleurosigma inflatum* Shadbolt | ***X*** | ***–*** | ***–*** | ***X*** | ***–*** |  | ***–*** |  |
| 871 | *Pleurosigma intermedium* W.Smith | ***X*** | ***–*** | ***–*** | ***–*** | ***–*** | ***X (Porifera)*** | ***–*** |  |
| 872 | *Pleurosigma normanii* Ralfs | ***X*** | ***–*** | ***–*** | ***X*** | ***–*** |  | ***–*** |  |
| 873 | *Pleurosigma obscurum* W.Smith | ***X*** | ***–*** | ***–*** | ***–*** | ***X*** |  | ***–*** |  |
| 874 | *Pleurosigma patagonicum var. paucistriatum* Sterrenburg et Sunesen | ***X*** | ***–*** | ***X*** | ***–*** | ***–*** |  | ***–*** |  |
| 875 | *Pleurosigma salinarum* (Grunow) Cleve et Grunow | ***X*** | ***–*** | ***–*** | ***–*** | ***X*** |  | ***–*** |  |
| 876 | *Pleurosigma* spp*.* W.Smith | ***X*** | ***–*** | ***–*** | ***X*** | ***X*** | ***X (Porifera)*** | ***–*** |  |
| 877 | *Pleurosira laevis* (Ehrenberg) Compère | ***–*** | ***X*** | ***–*** | ***X*** | ***–*** |  | ***–*** |  |
| 878 | *Plumosigma hustedtii*^[1][2]^ Nemoto | ***X*** | ***–*** | ***X*^[1]^** | ***–*** | ***–*** |  | **^[1][2]^Artiodactyla** | **Nemoto (1956)** |
| 889 | *Plumosigma rimosum*^[1][2]^ Nemoto | ***X*** | ***–*** | ***X*^[2]^** | ***–*** | ***–*** |  | **^[1][2]^Artiodactyla** | **Nemoto (1956)** |
| 880 | *Plumosigma* spp.*^.^*^[1][2]^ Nemoto | ***X*** | ***–*** | ***X*^[1]^** | ***–*** | ***–*** |  | **^[1][2]^Artiodactyla** | **Nemoto (1956)** |
| 881 | *Podosira stelligera* (Bailey) D.G.Mann | ***X*** | ***–*** | ***–*** | ***X*** | ***–*** |  | ***–*** |  |
| 882 | *Porannulus contentus* Hamilton, Kloser et Poulin | ***X*** | ***–*** | ***–*** | ***–*** | ***–*** | ***X (Porifera)*** | ***–*** |  |
| 883 | *Poulinea cf. lepidochelicola*^[1][2]^ Majewska, M.De Stefano et Van de Vijver | ***X*** | ***–*** | ***X*^[1]^** | ***–*** | ***–*** |  | **^[1][2]^Testudines (*Lepidochelys olivacea*)** | **Majewska *et al*. (2015*b*)** |
| 884 | *Poulinea lepidochelicola*^[1][2]^ Majewska, M.De Stefano et Van de Vijver | ***X*** | ***–*** | ***X*^[1]^** | ***–*** | ***–*** |  | **^[1][2]^Testudines (*Lepidochelys olivacea*)** | **Majewska *et al*. (2015*b*)** |
| 885 | *Poulinea* spp*^.^*^[1][2]^ Majewska, M.De Stefano et Van de Vijver | ***X*** | ***–*** | ***X*^[1]^** | ***–*** | ***–*** |  | **^[2]^Testudines (*Lepidochelys olivacea*)** | **Majewska *et al*. (2015*b*)** |
| 886 | *Prestauroneis protracta* (Grunow) Liu et Kociolek | ***–*** | ***X*** | ***X*** | ***–*** | ***–*** |  | ***–*** |  |
| 887 | *Proschkinia* spp*.* Karayeva | ***X*** | ***–*** | ***X*** | ***–*** | ***–*** |  | ***–*** |  |
| 888 | *Proschkinia sulcata* Majewska, Van de Vijver et Bosak | ***X*** | ***–*** | ***X*** | ***–*** | ***–*** |  | ***–*** |  |
| 889 | *Proschkinia vergostriata* Frankovich, Ashworth et Sullivan | ***X*** | ***–*** | ***X*** | ***–*** | ***–*** |  | ***–*** |  |
| 890 | *Protoraphis atlantica*^[1][2]^ Gibson | ***X*** | ***–*** | ***–*** | ***–*** | ***X*^[1]^** |  | **^[2]^Calanoida (genus *Candacia*)** | **Gibson (1979)** |
| 891 | *Protoraphis hustedtiana*^[1][2]^ Simonsen | ***X*** | ***–*** | ***–*** | ***–*** | ***X*^[1]^** |  | **^[2]^Calanoida (genus *Candacia*)** | **Simonsen (1970)** |
| 892 | *Protoraphis hustedtiana var. hustedtiana*^[1][2]^ Simonsen | ***–*** | ***–*** | ***–*** | ***–*** | ***X*^[1]^** |  | ***–*** | **Simonsen (1970)** |
| 893 | *Protoraphis* spp*.*^[1][2]^ Simonsen | ***X*** | ***–*** | ***–*** | ***–*** | ***X*^[1]^** |  | ***–*** | **Simonsen (1970)** |
| 894 | *Psammodictyon constrictum* (W.Gregory) D.G.Mann | ***X*** | ***–*** | ***X*** | ***–*** | ***X*** |  | ***–*** |  |
| 895 | *Psammodictyon mediterraneum* (Hustedt) D.G.Mann | ***X*** | ***–*** | ***–*** | ***–*** | ***–*** | ***X (Cnidaria)*** | ***–*** |  |
| 896 | *Psammodictyon panduriforme* (W.Gregory) D.G.Mann | ***X*** | ***–*** | ***X*** | ***X*** | ***X*** | ***X (Bryozoa)*** | ***–*** |  |
| 897 | *Psammodictyon panduriforme* var. *minor* (Grunow) Haworth et Kelly | ***X*** | ***–*** | ***–*** | ***X*** | ***–*** |  | ***–*** |  |
| 898 | *Psammodictyon rudum* (Cholnoky) D.G.Mann | ***X*** | ***–*** | ***X*** | ***–*** | ***–*** |  | ***–*** |  |
| 899 | *Psammodictyon* spp*.* D.G.Mann | ***X*** | ***–*** | ***X*** | ***X*** | ***X*** |  | ***–*** |  |
| 900 | *Psammodiscus nitidus* (W.Gregory) Round et D.G.Mann | ***X*** | ***–*** | ***–*** | ***X*** | ***–*** |  | ***–*** |  |
| 901 | *Psammothidium aff. marginulatum* (Grunow) Bukhtiyarova et Round | ***X*** | ***–*** | ***–*** | ***X*** | ***–*** |  | ***–*** |  |
| 902 | *Psammothidium bioretii* (H.Germain) Bukhtiyarova et Round | ***–*** | ***X*** | ***X*** | ***–*** | ***X*** |  | ***–*** |  |
| 903 | *Psammothidium oblongellum* (Østrup) Van de Vijver | ***–*** | ***X*** | ***–*** | ***–*** | ***X*** |  | ***–*** |  |
| 904 | *Psammothidium rechtense* (Leclercq) Lange-Bertalot | ***–*** | ***X*** | ***X*** | ***–*** | ***–*** |  | ***–*** |  |
| 905 | *Pseudachnanthidium* spp*.* Riaux-Gobin | ***X*** | ***–*** | ***–*** | ***X*** | ***–*** |  | ***–*** |  |
| 906 | *Pseudogomphonema kamtschaticum* (Grunow) Medlin | ***X*** | ***–*** | ***–*** | ***–*** | ***X*** |  | ***–*** |  |
| 907 | *Pseudohimantidium pacificum*^[1][2]^ Hustedt et Krasske | ***X*** | ***–*** | ***–*** | ***–*** | ***X*^[1][2]^** |  | **^[1][2]^Cyclopoida (*Corycaeus*)** | **Gibson (1978)** |
| 908 | *Pseudonitzschia fraudulenta* (Cleve) Hasle | ***X*** | ***–*** | ***–*** | ***X*** | ***–*** |  | ***–*** |  |
| 909 | *Pseudonitzschia* spp*.* Peragallo | ***X*** | ***–*** | ***–*** | ***–*** | ***X*** |  | ***–*** |  |
| 910 | *Pseudostaurosira brevistriata* (Grunow) D.M.Williams et Round | ***–*** | ***X*** | ***X*** | ***–*** | ***X*** |  | ***–*** |  |
| 911 | *Pseudostaurosira cf. perminuta* (Grunow) Sabbe et Vyverman | ***X*** | ***–*** | ***–*** | ***X*** | ***–*** |  | ***–*** |  |
| 912 | *Pseudostaurosira parasitica* (W.Smith) E.Morales | ***–*** | ***X*** | ***X*** | ***–*** | ***–*** |  | ***–*** |  |
| 913 | *Pseudostaurosira parasitica var. subconstricta* (Grunow) E.Morales | ***–*** | ***X*** | ***X*** | ***–*** | ***–*** |  | ***–*** |  |
| 914 | *Pseudostaurosira robusta* (Fusey) D.M.Williams et Round | ***–*** | ***X*** | ***X*** | ***–*** | ***–*** |  | ***–*** |  |
| 915 | *Pseudostaurosira trainorii* E.Morales | ***–*** | ***X*** | ***X*** | ***–*** | ***–*** |  | ***–*** |  |
| 916 | *Pteroncola inane* (Giffen) Round | ***X*** | ***–*** | ***X*** | ***–*** | ***–*** |  | ***–*** |  |
| 917 | *Pteroncola* spp*.* R.W.Holmes et Croll | ***X*** | ***–*** | ***–*** | ***–*** | ***–*** | ***X (Bryozoa)*** | ***–*** |  |
| 918 | *Ralfsiella W.Smithii* (Ralfs) P.A.Sims, D.M.Williams et Ashworth | ***X*** | ***–*** | ***–*** | ***–*** | ***X*** |  | ***–*** |  |
| 919 | *Reimeria sinuata* (W.Gregory) Kociolek et Stoermer | ***–*** | ***X*** | ***X*** | ***–*** | ***X*** |  | ***–*** |  |
| 920 | *Reimeria uniseriata* Sala, Guerrero et Ferrario | ***–*** | ***X*** | ***X*** | ***–*** | ***X*** |  | ***–*** |  |
| 921 | *Rhabdonema arcuatum* (Lyngbye) Kützing | ***X*** | ***–*** | ***–*** | ***X*** | ***–*** |  | ***–*** |  |
| 922 | *Rhabdonema* spp*.* Kützing | ***X*** | ***–*** | ***–*** | ***–*** | ***–*** | ***X (Bryozoa)*** | ***–*** |  |
| 923 | *Rhaphoneis amphiceros* (Ehrenberg) Ehrenberg | ***X*** | ***–*** | ***X*** | ***X*** | ***–*** | ***X (Bryozoa)*** | ***–*** |  |
| 924 | *Rhaphoneis castracanei* Grunow | ***X*** | ***–*** | ***–*** | ***–*** | ***X*** |  | ***–*** |  |
| 925 | *Rhaphoneis hungarica* Ehrenberg | ***X*** | ***–*** | ***X*** | ***–*** | ***–*** |  | ***–*** |  |
| 926 | *Rhaphoneis surirella* (Ehrenberg) Grunow | ***X*** | ***–*** | ***–*** | ***–*** | ***–*** | ***X (Bryozoa)*** | ***–*** |  |
| 927 | *Rhizosolenia arafurensis* Castracane | ***X*** | ***–*** | ***–*** | ***X*** | ***–*** |  | ***–*** |  |
| 928 | *Rhizosolenia* spp*.* T.Brightwell | ***X*** | ***–*** | ***–*** | ***X*** | ***X*** |  | ***–*** |  |
| 929 | *Rhizosolenia styliformis* T.Brightwell | ***X*** | ***–*** | ***X*** | ***X*** | ***–*** |  | ***–*** |  |
| 930 | *Rhoicosigma compactum* Greville | ***X*** | ***–*** | ***–*** | ***–*** | ***X*** |  | ***–*** |  |
| 931 | *Rhoicosphenia abbreviata* (C.Agardh) Lange-Bertalot | ***X*** | ***X*** | ***X*** | ***X*** | ***X*** |  | ***–*** |  |
| 932 | *Rhoicosphenia genuflexa* (Kützing) Medlin | ***X*** | ***–*** | ***–*** | ***X*** | ***–*** | ***X (Bryozoa)*** | ***–*** |  |
| 933 | *Rhoicosphenia marina* A.W.F.Schmidt | ***X*** | ***–*** | ***–*** | ***–*** | ***X*** | ***X (Bryozoa)*** | ***–*** |  |
| 934 | *Rhoicosphenia pullus* A.W.F.Schmidt | ***X*** | ***–*** | ***X*** | ***–*** | ***–*** |  | ***–*** |  |
| 935 | *Rhoicosphenia* spp*.* Grunow | ***X*** | ***–*** | ***–*** | ***–*** | ***X*** | ***X (Bryozoa, Annelida)*** | ***–*** |  |
| 936 | *Rhopalodia acuminata* Krammer | ***X*** | ***–*** | ***–*** | ***–*** | ***X*** |  | ***–*** |  |
| 937 | *Rhopalodia gibba* (Ehrenberg) O.F.Müller | ***–*** | ***X*** | ***–*** | ***X*** | ***–*** |  | ***–*** |  |
| 938 | *Rhopalodia gibba var. ventricosa* (Kützing) H.Peragallo et M.Peragallo | ***–*** | ***X*** | ***–*** | ***–*** | ***X*** |  | ***–*** |  |
| 939 | *Rhopalodia gibberula* (Ehrenberg) O.F.Müller | ***X*** | ***–*** | ***–*** | ***–*** | ***X*** |  | ***–*** |  |
| 940 | *Rhopalodia musculus* (Kützing) O.F.Müller | ***X*** | ***–*** | ***–*** | ***X*** | ***X*** |  | ***–*** |  |
| 941 | *Rhopalodia* spp*.* O.F.Müller | ***X*** | ***X*** | ***–*** | ***X*** | ***X*** |  | ***–*** |  |
| 942 | *Sameioneis carinaes* Russell et Norris | ***X*** | ***–*** | ***–*** | ***–*** | ***X*** |  | ***–*** |  |
| 943 | *Scalariella* spp*.* Riaux-Gobin | ***X*** | ***–*** | ***–*** | ***X*** | ***–*** |  | ***–*** |  |
| 944 | *Sceptronema orientale*^[1][2]^ Takano | ***X*** | ***–*** | ***–*** | ***–*** | ***X*^[1]^** |  | **^[1][2]^Harpacticoida (*Euterpina acutifrons*)** | **Takano (1983)** |
| 945 | *Schroederella* spp*.* Pavillard | ***X*** | ***–*** | ***–*** | ***–*** | ***X*** |  | ***–*** |  |
| 946 | *Scoliopleura* spp*.* Grunow | ***X*** | ***–*** | ***–*** | ***–*** | ***–*** | ***X (Annelida)*** | ***–*** |  |
| 947 | *Sellaphora atomoides* (Grunow) Wetzel et Van de Vijver | ***–*** | ***X*** | ***X*** | ***–*** | ***X*** |  | ***–*** |  |
| 948 | *Sellaphora bacillum* (Ehrenberg) D.G.Mann | ***–*** | ***X*** | ***–*** | ***–*** | ***X*** |  | ***–*** |  |
| 949 | *Sellaphora elliptica* Mereschkowsky | ***–*** | ***X*** | ***X*** | ***–*** | ***–*** |  | ***–*** |  |
| 950 | *Sellaphora nigri* (De Notaris) Wetzel et Ector | ***–*** | ***X*** | ***X*** | ***X*** | ***X*** |  | ***–*** |  |
| 951 | *Sellaphora pupula* (Kützing) Mereschkowsky | ***–*** | ***X*** | ***X*** | ***–*** | ***X*** |  | ***–*** |  |
| 952 | *Sellaphora saugerresii* (Desmazières) Wetzel et D.G.Mann | ***–*** | ***X*** | ***X*** | ***–*** | ***–*** |  | ***–*** |  |
| 953 | *Sellaphora seminulum* (Grunow) D.G.Mann | ***–*** | ***X*** | ***X*** | ***–*** | ***–*** |  | ***–*** |  |
| 954 | *Sellaphora* spp*.* Mereschkowsky | ***–*** | ***X*** | ***X*** | ***–*** | ***–*** |  | ***–*** |  |
| 955 | *Sellaphora stroemii* (Hustedt) H.Kobayasi | ***–*** | ***X*** | ***–*** | ***–*** | ***X*** |  | ***–*** |  |
| 956 | *Sellaphora ventraloides* (Hustedt) E.Falasco et Ector | ***–*** | ***X*** | ***–*** | ***–*** | ***X*** |  | ***–*** |  |
| 957 | *Seminavis robusta* Danielidis et D.G.Mann | ***X*** | ***–*** | ***–*** | ***–*** | ***X*** |  | ***–*** |  |
| 958 | *Seminavis* spp*.* D.G.Mann | ***X*** | ***–*** | ***X*** | ***–*** | ***X*** |  | ***–*** |  |
| 959 | *Seminavis strigosa* (Hustedt) Danielidis et Economou-Amilli | ***X*** | ***–*** | ***X*** | ***–*** | ***–*** |  | ***–*** |  |
| 960 | *Serratifera opephoroides* (Takano) Chunlian et Witkowski | ***X*** | ***–*** | ***–*** | ***X*** | ***–*** |  | ***–*** |  |
| 961 | *Shionodiscus cf. bioculatus* (Grunow) A.J.Alverson, S.H.Kang et E.C.Theriot | ***X*** | ***–*** | ***X*** | ***–*** | ***–*** |  | ***–*** |  |
| 962 | *Shionodiscus cf. oestrupii* (Ostenfeld) A.J.Alverson, S.H.Kang et E.C.Theriot | ***X*** | ***–*** | ***–*** | ***–*** | ***X*** |  | ***–*** |  |
| 963 | *Simonsenia delognei* Lange-Bertalot | ***–*** | ***X*** | ***–*** | ***–*** | ***X*** |  | ***–*** |  |
| 964 | *Skeletonema costatum* (Greville) Cleve | ***X*** | ***–*** | ***–*** | ***X*** | ***–*** |  | ***–*** |  |
| 945 | *Skeletonema* spp*.* Greville | ***X*** | ***–*** | ***X*** | ***–*** | ***X*** |  | ***–*** |  |
| 966 | *Stauroneis aleutica* Nemoto | ***X*** | ***–*** | ***X*** | ***–*** | ***–*** |  | ***–*** |  |
| 967 | *Stauroneis anceps* Ehrenberg | ***–*** | ***X*** | ***X*** | ***–*** | ***–*** |  | ***–*** |  |
| 968 | *Stauroneis olympica* Hustedt | ***X*** | ***–*** | ***X*** | ***–*** | ***–*** |  | ***–*** |  |
| 969 | *Stauroneis* spp*.* Ehrenberg | ***–*** | ***X*** | ***X*** | ***–*** | ***–*** |  | ***–*** |  |
| 970 | *Staurosira binodis* (Ehrenberg) Lange-Bertalot | ***–*** | ***X*** | ***X*** | ***–*** | ***X*** |  | ***–*** |  |
| 971 | *Staurosira mutabilis* (W.Smith) Grunow | ***–*** | ***X*** | ***–*** | ***–*** | ***X*** |  | ***–*** |  |
| 972 | *Staurosira* spp*.* (Ehrenberg)D.M.Williams et Round | ***–*** | ***X*** | ***X*** | ***–*** | ***–*** |  | ***–*** |  |
| 973 | *Staurosira sviridae* Kulikovskiy, Genkal et Mikheeva | ***–*** | ***X*** | ***X*** | ***–*** | ***–*** |  | ***–*** |  |
| 974 | *Staurosira venter* (Ehrenberg) Cleve et Mӧller | ***–*** | ***X*** | ***X*** | ***–*** | ***X*** |  | ***–*** |  |
| 975 | *Staurosirella leptostauron* (Ehrenberg) D.M.Williams et Round | ***–*** | ***X*** | ***X*** | ***–*** | ***–*** |  | ***–*** |  |
| 976 | *Staurosirella martyi* (Héribaud) Morales et Manoylov | ***X*** | ***–*** | ***–*** | ***–*** | ***X*** |  | ***–*** |  |
| 977 | *Staurosirella mutabilis* (W.Smith) Morales et Van de Vijver | ***–*** | ***X*** | ***X*** | ***–*** | ***–*** |  | ***–*** |  |
| 978 | *Staurosirella neopinnata* Morales, Wetzel, Haworth et Ector | ***–*** | ***X*** | ***X*** | ***–*** | ***–*** |  | ***–*** |  |
| 979 | *Stephanodiscus minutulus* Grunow | ***X*** | ***–*** | ***–*** | ***X*** | ***–*** |  | ***–*** |  |
| 9801 | *Stephanodiscus* spp*.* Ehrenberg | ***X*** | ***X*** | ***X*** | ***X*** | ***–*** |  | ***–*** |  |
| 981 | *Stephanopyxis nipponica* Gran et Yendo | ***X*** | ***–*** | ***–*** | ***X*** | ***–*** |  | ***–*** |  |
| 982 | *Stephanopyxis palmeriana* (Greville) Grunow | ***X*** | ***–*** | ***–*** | ***X*** | ***–*** |  | ***–*** |  |
| 983 | *Striatella* spp*.* C.Agardh | ***X*** | ***–*** | ***–*** | ***–*** | ***–*** | ***X (Bryozoa)*** | ***–*** |  |
| 984 | *Striatella unipunctata* (Lyngbye) C.Agardh | ***X*** | ***–*** | ***X*** | ***–*** | ***–*** |  | ***–*** |  |
| 985 | *Surirella angusta* Kützing | ***–*** | ***X*** | ***X*** | ***–*** | ***–*** |  | ***–*** |  |
| 986 | *Surirella atomus* Hustedt | ***X*** | ***–*** | ***–*** | ***X*** | ***–*** |  | ***–*** |  |
| 987 | *Surirella Brébissonii* Krammer et Lange-Bertalot | ***–*** | ***X*** | ***X*** | ***–*** | ***–*** |  | ***–*** |  |
| 988 | *Surirella Brébissonii var. kuetzingii* Krammer et Lange-Bertalot | ***–*** | ***X*** | ***X*** | ***–*** | ***–*** |  | ***–*** |  |
| 989 | *Surirella fastuosa* Ehrenberg | ***X*** | ***–*** | ***–*** | ***X*** | ***–*** |  | ***–*** |  |
| 990 | *Surirella lacrimula* J.D.English | ***–*** | ***X*** | ***X*** | ***–*** | ***–*** |  | ***–*** |  |
| 991 | *Surirella librile* (Ehrenberg) Ehrenberg | ***–*** | ***X*** | ***X*** | ***–*** | ***–*** |  | ***–*** |  |
| 992 | *Surirella minuta* Brébisson | ***–*** | ***X*** | ***–*** | ***–*** | ***X*** |  | ***–*** |  |
| 993 | *Surirella ovalis* Brébisson | ***X*** | ***–*** | ***–*** | ***–*** | ***X*** |  | ***–*** |  |
| 994 | *Surirella ovata* Kützing | ***X*** | ***X*** | ***–*** | ***–*** | ***X*** | ***X (Cnidaria)*** | ***–*** |  |
| 995 | *Surirella* spp*.* Turpin | ***X*** | ***X*** | ***X*** | ***X*** | ***X*** |  | ***–*** |  |
| 996 | *Surirella striatula* Turpin | ***X*** | ***–*** | ***–*** | ***–*** | ***X*** |  | ***–*** |  |
| 997 | *Surirella suecica* Grunow | ***–*** | ***X*** | ***X*** | ***–*** | ***–*** |  | ***–*** |  |
| 998 | *Synedra amphicephala* Kützing | ***X*** | ***–*** | ***–*** | ***–*** | ***X*** |  | ***–*** |  |
| 999 | *Synedra berolinensis* Lemmermann | ***X*** | ***–*** | ***–*** | ***–*** | ***X*** |  | ***–*** |  |
| 1000 | *Synedra camtschatica* Grunow | ***X*** | ***–*** | ***X*** | ***–*** | ***–*** |  | ***–*** |  |
| 1001 | *Synedra cf. fasciculata* Kützing | ***–*** | ***–*** | ***–*** | ***X*** | ***–*** |  | ***–*** |  |
| 1002 | *Synedra cyclopum*^[1][2]^ Brutschy | ***–*** | ***X*** | ***–*** | ***–*** | ***X*^[1]^** |  | **^[2]^Arthropoda (genus *Daphnia*)** | **Brutschy (1922)** |
| 1003 | *Synedra cyclopum var. 2*^[1][2]^ Schulz | ***–*** | ***X*** | ***–*** | ***–*** | ***X*^[1]^** |  | **^[2]^Arthropoda (genus *Daphnia*)** | **Brutschy (1922)** |
| 1004 | *Synedra cyclopum var. incisa*^[1][2]^ Cunningham | ***–*** | ***X*** | ***–*** | ***–*** | ***X*^[1]^** |  | **^[2]^Arthropoda (genus *Daphnia*)** | **Brutschy (1922)** |
| 1005 | *Synedra delicatissima* W.Smith | ***–*** | ***X*** | ***–*** | ***X*** | ***X*** |  | ***–*** |  |
| 1006 | *Synedra hennedyana* W.Gregory | ***X*** | ***–*** | ***X*** | ***–*** | ***–*** |  | ***–*** |  |
| 1007 | *Synedra karcheri***^[^**^1][2]^ Nemoto | ***X*** | ***–*** | ***X*^[1]^** | ***–*** | ***–*** |  | **^[1][2]^Artiodactyla** | **Nemoto (1956)** |
| 1008 | *Synedra pulchella* (Ralfs) Kützing | ***X*** | ***–*** | ***–*** | ***–*** | ***X*** |  | ***–*** |  |
| 1009 | *Synedra socia* J.H.Wallace | ***–*** | ***X*** | ***–*** | ***–*** | ***X*** |  | ***–*** |  |
| 1010 | *Synedra* spp*.* Ehrenberg | ***X*** | ***X*** | ***X*** | ***X*** | ***X*** | ***X (Annelida)*** | ***–*** |  |
| 1011 | *Synedra tabulata* (C.Agardh) Kützing | ***X*** | ***–*** | ***X*** | ***–*** | ***–*** |  | ***–*** |  |
| 1012 | *Synedra ulna var. oxyrhyncha f. mediocontracta* (Kützing) O'Meara | ***–*** | ***X*** | ***–*** | ***–*** | ***X*** |  | ***–*** |  |
| 1013 | *Tabellaria fenestrata* (Lyngbye) Kützing | ***–*** | ***X*** | ***X*** | ***–*** | ***–*** |  | ***–*** |  |
| 1014 | *Tabellaria* spp*.* Ehrenberg | ***X*** | ***X*** | ***X*** | ***X*** | ***–*** | ***X (Bryozoa)*** | ***–*** |  |
| 1015 | *Tabularia affinis* (Kützing) Snoeijs | ***X*** | ***–*** | ***X*** | ***–*** | ***X*** |  | ***–*** |  |
| 1016 | *Tabularia fasciculata* (C.Agardh) D.M.Williams et Round | ***X*** | ***X*** | ***X*** | ***X*** | ***X*** | ***X (Bryozoa)*** | ***–*** |  |
| 1017 | *Tabularia investiens* (W.Smith)D.M.Williams et Round | ***X*** | ***–*** | ***–*** | ***X*** | ***X*** |  | ***–*** |  |
| 1018 | *Tabularia parva* (Kützing) D.M.Williams et Round | ***X*** | ***–*** | ***–*** | ***X*** | ***X*** |  | ***–*** |  |
| 1019 | *Tabularia* spp*.* D.M.Williams et Round | ***X*** | ***–*** | ***–*** | ***X*** | ***X*** | ***X (Bryozoa)*** | ***–*** |  |
| 1020 | *Tabularia tabulata* (C.Agardh) Snoeijs | ***X*** | ***–*** | ***–*** | ***–*** | ***X*** | ***X (Cnidaria)*** | ***–*** |  |
| 1021 | *Thacyneis aspera* (Ehrenberg) Cleve | ***X*** | ***–*** | ***X*** | ***–*** | ***–*** |  | ***–*** |  |
| 1022 | *Thalassionema frauenfeldii* (Grunow) Tempére et Peragallo | ***X*** | ***–*** | ***–*** | ***X*** | ***–*** |  | ***–*** |  |
| 1023 | *Thalassionema nitzschioides* (Grunow) Mereschkowsky | ***X*** | ***–*** | ***X*** | ***–*** | ***X*** |  | ***–*** |  |
| 1024 | *Thalassionema* spp*.* Grunow | ***X*** | ***–*** | ***–*** | ***–*** | ***X*** |  | ***–*** |  |
| 1025 | *Thalassiosira angulata* (W.Gregory) Hasle | ***X*** | ***–*** | ***–*** | ***X*** | ***–*** |  | ***–*** |  |
| 1026 | *Thalassiosira decipiens* (Grunow) Jørgensen | ***X*** | ***–*** | ***–*** | ***–*** | ***–*** | ***X (Bryozoa)*** | ***–*** |  |
| 1027 | *Thalassiosira eccentrica* (Ehrenberg) Cleve | ***X*** | ***–*** | ***–*** | ***–*** | ***X*** |  | ***––*** |  |
| 1028 | *Thalassiosira grunowii* Akiba et Yanagisawa | ***X*** | ***–*** | ***–*** | ***X*** | ***–*** |  | ***–*** |  |
| 1029 | *Thalassiosira guillardii* Halse | ***X*** | ***–*** | ***–*** | ***X*** | ***–*** |  | ***–*** |  |
| 1030 | *Thalassiosira laevis* Gao et Cheng | ***X*** | ***–*** | ***–*** | ***–*** | ***X*** |  | ***–*** |  |
| 1031 | *Thalassiosira maculata* G.A.Fryxell et Johansen | ***X*** | ***–*** | ***X*** | ***–*** | ***–*** |  | ***–*** |  |
| 1032 | *Thalassiosira minima* Gaarder | ***X*** | ***–*** | ***–*** | ***X*** | ***–*** |  | ***–*** |  |
| 1033 | *Thalassiosira nordenskioeldii* Cleve | ***X*** | ***–*** | ***X*** | ***–*** | ***–*** |  | ***–*** |  |
| 1034 | *Thalassiosira proschkinae* Makarova | ***X*** | ***–*** | ***–*** | ***X*** | ***–*** |  | ***–*** |  |
| 1035 | *Thalassiosira pseudonana* Hasle et Heimdal | ***X*** | ***–*** | ***–*** | ***X*** | ***–*** |  | ***–*** |  |
| 1036 | *Thalassiosira* spp*.* Cleve | ***X*** | ***–*** | ***X*** | ***X*** | ***X*** | ***X (Bryozoa, Porifera)*** | ***–*** |  |
| 1037 | *Thalassiosira visurgis* Hustedt | ***X*** | ***–*** | ***–*** | ***X*** | ***–*** |  | ***–*** |  |
| 1038 | *Thalassiothrix frauenfeldii* (Grunow) Grunow | ***X*** | ***–*** | ***X*** | ***–*** | ***–*** |  | ***–*** |  |
| 1039 | *Thalassiothrix longissima* Cleve et Grunow | ***X*** | ***–*** | ***X*** | ***–*** | ***–*** |  | ***–*** |  |
| 1040 | *Thalassiothrix* spp*.* Cleve et Grunow | ***X*** | ***–*** | ***X*** | ***–*** | ***X*** |  | ***–*** |  |
| 1041 | *Toxarium undulatum* Bailey | ***X*** | ***–*** | ***–*** | ***–*** | ***–*** | ***X (Cnidaria)*** | ***–*** |  |
| 1042 | *Trachyneis aspera* (Ehrenberg) Cleve | ***X*** | ***–*** | ***–*** | ***X*** | ***X*** | ***X (Bryozoa, Porifera)*** | ***–*** |  |
| 1043 | *Trachyneis aspera var. oblonga* (Bailey) Cleve | ***X*** | ***–*** | ***X*** | ***–*** | ***–*** |  | ***–*** |  |
| 1044 | *Trachyneis aspera var. robusta* (P.Petit) Cleve | ***X*** | ***–*** | ***X*** | ***–*** | ***–*** |  | ***–*** |  |
| 1045 | *Trachyneis velata* A.W.F.Schmidt | ***X*** | ***–*** | ***–*** | ***–*** | ***X*** |  | ***–*** |  |
| 1046 | *Triblyionella marginulata var. subconstricta f. minta* (Grunow) Poulin | ***X*** | ***–*** | ***–*** | ***–*** | ***–*** | ***X (Cnidaria)*** | ***–*** |  |
| 1074 | *Trigonium arcticum* (Brightwell) Cleve | ***X*** | ***–*** | ***X*** | ***–*** | ***–*** |  | ***–*** |  |
| 1048 | *Tripterion kalamensis*^[1][2]^ R.W.Holmes, Nagasawa et Takano | ***X*** | ***–*** | ***X*^[1]^** | ***–*** | ***–*** |  | **^[1][2]^Artiodactyla** | **Holmes *et al*. (1993)** |
| 1049 | *Tripterion societatis*^[1][2]^ Riaux-Gobin, Witkowski et Ector | ***X*** | ***–*** | ***X*^[1]^** | ***–*** | ***–*** |  | **^[1][2]^Testudines (*Chelonia mydas*)** | **Riaux-Gobin *et al*. (2017*a*)** |
| 1050 | *Tripterion* spp*.*^[1]^ R.W.Holmes, Nagasawa et Takano | ***X*** | ***–*** | ***X^[1]^*** | ***–*** | ***–*** |  | ***–*** |  |
| 1051 | *Tropidoneis* spp*.* Cleve | ***X*** | ***–*** | ***–*** | ***–*** | ***–*** | ***X (Annelida)*** | ***–*** |  |
| 1052 | *Tryblionella angustata* W.Smith | ***–*** | ***X*** | ***X*** | ***–*** | ***–*** |  | ***–*** |  |
| 1053 | *Tryblionella apiculata* W.Gregory | ***X*** | ***X*** | ***X*** | ***X*** | ***–*** |  | ***–*** |  |
| 1054 | *Tryblionella brunoi* (Lange-Bertalot) Cantonati et Lange-Bertalot | ***–*** | ***X*** | ***X*** | ***–*** | ***–*** |  | ***–*** |  |
| 1055 | *Tryblionella coarctata* (Grunow) D.G.Mann | ***X*** | ***–*** | ***–*** | ***–*** | ***X*** |  | ***–*** |  |
| 1056 | *Tryblionella granulata* (Grunow) D.G.Mann | ***X*** | ***–*** | ***X*** | ***–*** | ***–*** |  | ***–*** |  |
| 1057 | *Tryblionella hungarica* (Grunow) D.G.Mann | ***X*** | ***X*** | ***X*** | ***–*** | ***X*** |  | ***–*** |  |
| 1058 | *Tryblionella hyalina* (Amossè) T.Ohtsuka | ***X*** | ***–*** | ***–*** | ***–*** | ***X*** |  | ***–*** |  |
| 1059 | *Tryblionella kuetzingii* Álvarez-Blanco et S.Blanco | ***–*** | ***X*** | ***X*** | ***–*** | ***–*** |  | ***–*** |  |
| 1060 | *Tryblionella littoralis* (Grunow) D.G.Mann | ***–*** | ***X*** | ***X*** | ***–*** | ***–*** |  | ***–*** |  |
| 1061 | *Tryblionella pararostrata* (Lange-Bertalot) Clavero et Hernández-Mariné | ***X*** | ***–*** | ***X*** | ***–*** | ***X*** |  | ***–*** |  |
| 1062 | *Tryblionella salinarum* (Grunow) Pelletan | ***–*** | ***X*** | ***X*** | ***–*** | ***–*** |  | ***–*** |  |
| 1063 | *Tryblionella* spp*.* W.Smith | ***X*** | ***X*** | ***X*** | ***–*** | ***X*** |  | ***–*** |  |
| 1064 | *Tursciocola costata*^[1][2]^ Frankovich et Sullivan | ***X*** | ***–*** | ***X*^[1]^** | ***–*** | ***–*** |  | **^[1][2]^Sirenia (*Trichecus manatus*)** | **Frankovich *et al*. (2015*a*)** |
| 1065 | *Tursiocola alata*^[1][2]^ Frankovich, Ashworth et Sullivan | ***X*** | ***–*** | ***X*^[1]^** | ***–*** | ***–*** |  | **^[2]^Sirenia (*Trichecus manatus latirostris*)** | **Frankovich *et al*. (2018)** |
| 1066 | *Tursiocola bondei*^[1][2]^ Frankovich, Ashworth et Sullivan | ***X*** | ***–*** | ***X*^[1]^** | ***–*** | ***–*** |  | **^[2]^Sirenia (*Trichecus manatus latirostris*)** | **Frankovich *et al*. (2018)** |
| 1067 | *Tursiocola denysii*^[1][2]^ Frankovich et Sullivan | ***X*** | ***–*** | ***X*^[1]^** | ***–*** | ***–*** |  | **^[2]^Testudines (*Caretta caretta*)** | **Frankovich *et al*. (2015*b*)** |
| 1068 | *Tursiocola gracilis*^[1][2]^ Frankovich, Ashworth et Sullivan | ***X*** | ***–*** | ***X*^[1]^** | ***–*** | ***–*** |  | **^[2]^Sirenia (*Trichecus manatus latirostris*)** | **Frankovich *et al*. (2018)** |
| 1069 | *Tursiocola guyanensis*^[1][2]^ Riaux-Gobin et Witkowski | ***X*** | ***–*** | ***X*^[1]^** | ***–*** | ***–*** |  | **^[2]^Testudines (*Chelonia mydas*)** | **Riaux-Gobin *et al*. (2017*a*)** |
| 1070 | *Tursiocola neliana*^[1][2]^ Majewska | ***X*** | ***–*** | ***X*^[1]^** | ***–*** | ***–*** |  |  | **Majewska *et al*. (2020)** |
| 1071 | *Tursiocola olympica* (Hustedt) R.W.Holmes, Nagasawa et Takano | ***X*** | ***–*** | ***–*** | ***–*** | ***X*** |  | ***–*** |  |
| 1072 | *Tursiocola omurai*^[1][2]^ (T.Nemoto) L.Denys | ***X*** | ***–*** | ***X*^[1]^** | ***–*** | ***–*** |  | **^[2]^Artiodactyla (*Physeter macrocephalus*)** | **Denys (1997)** |
| 1073 | *Tursiocola* spp*.*^[1][2]^ R.W.Holmes, Nagasawa et Takano | ***X*** | ***–*** | ***X*^[1]^** | ***–*** | ***–*** |  | **^[2]^Artiodactyla** | **Holmes *et al*. (1993)** |
| 1074 | *Tursiocola staurolineata*^[1][2]^ Denys | ***X*** | ***–*** | ***X*^[1]^** | ***–*** | ***–*** |  | **^[2]^Artiodactyla (*Physeter macrocephalus*)** | **Denys (1997)** |
| 1075 | *Tursiocola varicopulifera*^[1][2]^ Frankovich et Sullivan | ***X*** | ***–*** | ***X*^[1]^** | ***–*** | ***–*** |  | **^[2]^Testudines (*Caretta caretta*)** | **Frankovich *et al*. (2015*a*)** |
| 1076 | *Tursiocola yin-yangii*^[1][2]^ Riaux-Gobin et Witkowski | ***X*** | ***–*** | ***X*^[1]^** | ***–*** | ***–*** |  | **^[2]^Testudines (*Chelonia mydas*)** | **Riaux-Gobin *et al*. (2017*a*)** |
| 1077 | *Tursiocola ziemanii*^[1][2]^ Frankovich et Sullivan | ***X*** | ***–*** | ***X*^[1]^** | ***–*** | ***–*** |  | **^[2]^Testudines (*Caretta caretta*)** | **Frankovich *et al*. (2015*a*)** |
| 1078 | *Ulnaria acus* (Kützing) Aboal | ***–*** | ***X*** | ***X*** | ***–*** | ***X*** |  | ***–*** |  |
| 1079 | *Ulnaria biceps* (Kützing) Compère | ***–*** | ***X*** | ***X*** | ***–*** | ***X*** |  | ***–*** |  |
| 1080 | *Ulnaria ulna* (Nitzsch) Compère | ***–*** | ***X*** | ***X*** | ***X*** | ***X*** |  | ***–*** |  |
