# Supplementary material for: Partners or passengers? Revisiting the association between diatoms and aquatic animals
Source: Biol Rev Camb Philos Soc. 2026 Jun 5;101(6):2597–612. doi: 10.1002/brv.70191 (PMC13736808; doi:10.1002/brv.70191)
Supplement: Supplementary file 2 [file BRV-101-2597-s003.docx]

**Table S1.** Summary of studies on epizoic diatoms included in this review. For each year the table reports the total number of studies, the environments studied and the continents where the studies were conducted in each year, the diatom identification method, and the phylum of the basibiont. Note: studies conducted across more than one continent or on more than one basibiont, but reported within the same article, are shown as ‘intercontinental’ (for studies spanning multiple continents) and ‘several basibionts’ (for studies involving multiple phyla).

| Year | n° | Environment | Continent | Method | Phylum |
| --- | --- | --- | --- | --- | --- |
| 1920 | 1 | Marine (1) | Antarctica (1) | Morphological (1) | Chordata (1) |
| 1921 | 1 | Marine (1) | Europe (1) | Morphological (1) | Arthropoda (1) |
| 1950 | 1 | Marine (1) | Antarctica (1) | Direct observation (1) | Chordata (1) |
| 1952 | 1 | Marine (1) | Antarctica (1) | Morphological (1) | Chordata (1) |
| 1954 | 1 | Marine (1) | Antarctica (1) | Morphological (1) | Chordata (1) |
| 1956 | 1 | Marine (1) | Intercontinental (1) | Morphological (1) | Chordata (1) |
| 1958 | 1 | Marine (1) | Antarctica (1) | Morphological (1) | Chordata (1) |
| 1965 | 1 | Freshwater (1) | North America (1) | Morphological (1) | Arthropoda (1) |
| 1971 | 1 | Marine (1) | North America (1) | Morphological (1) | Arthropoda (1) |
| 1977 | 1 | Marine (1) | South America (1) | Morphological (1) | Chordata (1) |
| 1978 | 2 | Marine (2) | North America (1), Africa (1) | Morphological (2) | Arthropoda (1), Chordata (1) |
| 1980 | 2 | Freshwater (1), Marine (1) | Asia (1), Europe (1) | Morphological (2) | Chordata (1), Arthropoda (1) |
| 1983 | 1 | Marine (1) | Asia (1) | Morphological (1) | Arthropoda (1) |
| 1984 | 1 | Marine (1) | South America (1) | Morphological (1) | Arthropoda (1) |
| 1989 | 1 | Marine and Culture (1) | North America (1) | Morphological (1) | Arthropoda (1) |
| 1990 | 2 | Marine (1), Marine and Culture (1) | Intercontinental (1), Oceania (1) | Morphological (2) | Chordata (1), Arthropoda (1) |
| 1993 | 3 | Freshwater (2), Marine (1) | North America (2), Intercontinental (1) | Morphological (3) | Arthropoda (3) |
| 1995 | 1 | Marine (1) | Oceania (1) | Morphological (1) | Mollusca (1) |
| 1996 | 3 | Freshwater (1), Marine (2) | Oceania (1), Asia (1), North America (1) | Morphological (3) | Chordata (2), Arthropoda (1) |
| 1997 | 2 | Marine (2) | America (1), Europe (1) | Morphological (2) | Chordata (2) |
| 1998 | 3 | Freshwater (1), Marine (1), Marine and Culture (1) | North America (2), Europe (1) | Morphological (3) | Mollusca (1); Arthropoda (1), Chordata (1) |
| 2000 | 3 | Marine (3) | Asia (1), Europe (1), Oceania (1) | Morphological (3) | Arthropoda (2), Mollusca (1) |
| 2001 | 2 | Marine (2) | North America (1), South America (1) | Morphological (2) | Chordata (2) |
| 2002 | 3 | Freshwater (1), Marine (2) | Asia (2), America (1) | Morphological (2) | Mollusca (1), Annelida (1), Arthropoda (1) |
| 2003 | 1 | Marine (1) | Europe (1) | Morphological (1) | Bryozoa (1) |
| 2004 | 2 | Marine (2) | Asia (1), Antarctica (1) | Morphological (2) | Porifera (1), Bryozoa (1) |
| 2005 | 5 | Marine (4), Brackish (1) | South America (1), North America (1), Intercontinental (1), Europe (1), Antarctica (1) | Morphological (4), Morphological and DNA (1) | Chordata (2), Porifera (1), Cnidaria (1), Bryozoa (1) |
| 2006 | 1 | Marine (1) | Europe (1) | Morphological (1) | Mollusca (1) |
| 2007 | 2 | Marine (1), Freshwater (1) | Intercontinental (1), North America (1) | Morphological (1), Morphological and DNA (1) | Mollusca (1), Chordata (1) |
| 2008 | 1 | Marine (1) | Europe (1) | Morphological (1) | Cnidaria (1) |
| 2009 | 2 | Freshwater (1), Marine (1) | South America (2) | Morphological (2) | Mollusca (1), Arthropoda (1) |
| 2010 | 4 | Marine (2), Freshwater (1), Brackish (1) | Europe (3), America (1) | Morphological (4) | Chordata (2), Mollusca (2) |
| 2011 | 1 | Marine (1) | Europe (1) | DNA (1) | Chordata (1) |
| 2012 | 4 | Marine (3), Freshwater (1) | South America (2), Africa (2) | Morphological (4) | Chordata (1), Arthropoda (2), Echinodermata (1) |
| 2013 | 1 | Freshwater (1) | North America (1) | Morphological (1) | Arthropoda (1) |
| 2014 | 3 | Marine (2), Freshwater (1) | Europe (2), America (1) | Morphological (3) | Chordata (1), Arthropoda (1), Cnidaria (1) |
| 2015 | 6 | Marine (6) | South America (2), North America (2), Asia (2) | Morphological (6) | Chordata (4), Arthropoda (2) |
| 2016 | 6 | Marine (5), Freshwater (1) | North America (3), South America (1), Asia (1), Europe (1) | Morphological (6) | Chordata (3), Arthropoda (3) |
| 2017 | 7 | Marine (5), Freshwater (2) | North America (1), South America (2), Europe (2), Africa (2) | Morphological (7) | Chordata (5), Mollusca (2) |
| 2018 | 7 | Marine (4), Freshwater (2), Marine and Culture (1) | America (5), Europe (2) | Morphological (5), Morphological and DNA (2) | Chordata (5), Arthropoda (2) |
| 2019 | 4 | Marine (4) | North America (1), South America (1), Intercontinental (1), Africa (1) | Morphological (2), Morphological and DNA (1), DNA (1) | Chordata (4) |
| 2020 | 10 | Marine (7), Museum (1), Captivity (1), Marine and Culture (1) | Africa (2), Europe (4), Asia (3), Inrtercontinental (1) | Morphological (10) | Chordata (7), Arthropoda (3) |
| 2021 | 10 | Marine (5), Freshwater (3), Marine and Culture (1), Brackish (1), Captivity (1) | Africa (2), Intercontinental (3), Europe (4), South America (1) | Morphological (6), Morphological and DNA (4) | Chordata (5), Arthropoda (3), Mollusca (1), Several basibionts (1) |
| 2022 | 7 | Marine (7) | South America (1), Intercontinental (2), Africa (2), Europe (1), Asia (1) | Morphological (4), Morphological and DNA (1), DNA (2) | Chordata (4), Mollusca (1), Porifera (1), Arthropoda (1) |
| 2023 | 2 | Marine (2) | South America (1), Asia (1) | Morphological (2) | Chordata (1), Echinodermata (1) |
| 2024 | 4 | Marine (4) | Africa (2), Asia (1), South America (1) | Morphological (4) | Arthropoda (2), Mollusca (1), Several basibionts (1) |
| 2025 | 4 | Marine (2), Freshwater (1), Marine and Culture (1) | Europe (1), Asia (2), South America (1) | Morphological (4) | Chordata (1), Arthropoda (1), Mollusca (1), Several basibionts (1) |
| TOTAL | ***133*** |  |  |  |  |
