# Supplementary material for: Partners or passengers? Revisiting the association between diatoms and aquatic animals
Source: Biol Rev Camb Philos Soc. 2026 Jun 5;101(6):2597–612. doi: 10.1002/brv.70191 (PMC13736808; doi:10.1002/brv.70191)
Supplement: Supplementary file 3 [file BRV-101-2597-s004.docx]

**SUPPORTING INFORMATION**

**Fig. S1.** Graphical representation of the temporal distribution of studies conducted on epizoic diatoms (from 1920 to 2025) in all studied environments. Note: ‘Captivity’ refers to studies conducted on large animals kept in captivity (e.g. turtles). By contrast, ‘Culture’ refers to microscopic organisms maintained or grown in the laboratory.

**Fig. S2.** Spatial distribution of the literature published on epizoic diatoms. Note: when available, we used coordinates corresponding to a more detailed geographic level (e.g. for the Americas and Europe). Geographical coordinates were downloaded from Google-Earth Pro (7.3.6.10201) and the map was plotted using the RStudio packages ‘sf’ and ‘maps’ (Becker *et al*., 2018; South *et al*., 2017; R Core Team, 2022; Pebesma & Bivand, 2023).

**Fig. S3.** Graphical representation of the number of studies conducted on the different phyla of basibiont (left), the epibiont diatom identification method employed (middle) and the host environment considered (right).

**Fig. S4.** Number of diatom species found on different basibionts in (A) freshwater and (B) marine environments.

**Fig. S5.** Proportions of diatom size classes observed on the three main basibionts. We include only the taxa for which size information was available (Chordata: 443 classified taxa out of 563; Arthropoda: 327 classified taxa out of 461; Mollusca: 182 classified taxa out of 289). Each species was classified into a size class according to Rimet & Bouchez (2012) or using morphometric data provided by the OMNIDIA software.

**Fig. S6.** Number of epibiont diatom genera found on different basibiont taxa, across all habitats.
